# Machine Learning Identifies Pan-Cancer Landscape of Nrf2 Oxidative Stress Response Pathway-Related Genes

**DOI:** 10.1155/2022/8450087

**Published:** 2022-02-17

**Authors:** Na Li, Xianquan Zhan

**Affiliations:** ^1^Shandong Key Laboratory of Radiation Oncology, Shandong Cancer Hospital and Institute, Shandong First Medical University, 440 Jiyan Road, Jinan, Shandong 250117, China; ^2^Medical Science and Technology Innovation Center, Shandong First Medical University, Jinan, 6699 Qingdao Road, Jinan, Shandong 250117, China; ^3^Gastroenterology Research Institute and Clinical Center, Shandong First Medical University, 38 Wuying Shan Road, Jinan, Shandong 250031, China

## Abstract

**Background:**

Oxidative stress produced a large amount of reactive oxygen species (ROS), which played a pivotal role in balanced ability and determining cell fate. The activated Nrf2 signaling pathway that responds to the excessive ROS regulated the expressions of antiapoptotic proteins, antioxidative enzymes, drug transporters, and detoxifying factors.

**Methods:**

The Nrf2 signaling pathway-related genes that had a direct relationship with Nrf2, including ATF4, BACH1, CREBBP, CUL3, EIF2AK3, EP300, FOS, FOSL1, GSK3B, JUN, KEAP1, MAF, MAFF, MAFG, MAFK, MAPK1, MAPK3, MAPK7, MAPK8, MAPK9, PIK3CA, PRRT2, and RIT1, were selected to do a systematic pan-cancer analysis. The relationship of Nrf2 signaling pathway-related gene expressions with tumor mutation burden, microsatellite status, clinical characteristics, immune system, cancer stemness index, and drug sensitivity was calculated by the Spearson correlation analysis across 11,057 subjects representing 33 cancer types. The prognosis models in lung squamous carcinoma, breast cancer, and stomach cancer were constructed with the Cox multivariate regression analysis and least absolute shrinkage and selection operator (Lasso) regression.

**Results:**

Many Nrf2 signaling pathway-related genes were differently expressed between tumor and normal tissues. PIK3CA showed high mutation rate in pan-cancer. The expressions of Nrf2 signaling pathway-related genes were significantly related to tumor mutation burden, copy number variant, microsatellite instability score, survival rate, pathological stage, immune phenotype, immune score, immune cell, cancer stemness index, and drug sensitivity. The prognosis models were significantly associated with survival rate in lung squamous carcinoma, breast cancer, and stomach cancer; and the prognosis model-based riskscore was significantly associated with clinicopathological characteristics of each cancer.

**Conclusions:**

The study provided a comprehensive pan-cancer landscape of Nrf2 pathway-related genes. Based on the same Nrf2 pathway-related genes, the different prognosis models were constructed for different types of cancers.

## 1. Introduction

In response to oxidative stress and oxidative damage, the accumulation of reactive oxygen species (ROS) can result in initiating tumorigenesis, supporting transformation, and inducing proliferation and apoptosis of cancer cells [1]. Endogenous ROS could be produced by peroxisomes, mitochondria, and inflammatory cell activation. Exogenous ROS could be produced from ionizing radiation and xenobiotics [2]. While ROS formation was necessary to signal transduction in normal cell, excess ROS could directly affect modification of cellular macromolecules, specially causing genomic DNA mutations [3]. For example, 8-hydroxy deoxyguanosine can be formed with ROS increasing, which can help transform GC base pairs (guanine and cytosine) to TA base pairs (thymine and adenine) and directly link to mutagenesis [4]. Considerable studies focused on nongenotoxic and epigenetic effects of ROS in carcinogenesis. The effects of ROS on modulation of cell growth depended on an important factor concentration of ROS. At higher doses or exposure to ROS, the cells trend to necrosis or apoptosis. At lower doses or exposure to ROS, the cells trend to proliferation [5]. The targeted molecules that responded to excess ROS signaling messenger played critical roles in gene transcription pathways [6]. Nrf2 was one of the activated transcription factors at the high concentration of ROS and regulated downstream targeted genes to encode detoxifying factors, antiapoptotic proteins, antioxidative enzymes, and drug efflux transporters [7]. The Nrf2-keap1 complex acted as a cellular defense mechanism in cytoplasm. When cellular stress from endogenous and exogenous agents induced the increase of ROS and activation of Nrf2, keap1 could release from the Nrf2-keap1 complex. Thus, the free Nrf2 was transported from cytoplasm to nucleus to bind with antioxidant response element (ARE), which results in the translation of multiple antioxidant response genes [8]. In the process of Nrf2 responding to the increased ROS, various pathways and genes participated in activation of tyrosine kinases to dissociate Nrf2-keap1 complex, such as MAPK signaling pathway, PKC signaling pathway, and PI3K signaling pathway [9]. Some genes in those signaling pathways directly affected Nrf2 status, including ERK1/2, ERK5, JNK1/2, p38 MAPK, PKC, PERK, and GSK3*β* [10–12]. After Nrf2 was transported into the nucleus, multiple genes were involved in regulating Nrf2 to recognize the AREs, including small MAF, ATF4, JUN, CBP/P300, ERK1/2, c-FOS, FRA1, c-MAF, and BACH1 [13, 14]. After Nrf2 recognized the corresponding AREs, the downstream genes were initiated to produce some significant biological functions, including reducing oxidative damage, promoting tumorigenesis, regulating cell survival, transporting xenobiotics and metabolites, repairing the damaged proteins, and removing the damaged proteins [15].

The implication of NRF2 signaling in various cancers was emerging as research hotpot. NRF2 had the multifaceted roles and multistage processes in cancer progression, which indicated both tumor-suppressing and tumor-promoting effects [16]. NRF2 has direct and indirect association with the hallmarks of cancer. For example, NRF2 regulated the sustained proliferation signaling, and the proliferation rates of cell lines were significantly associated with NRF2 status. When NRF2 gene was knocked out with CRISPR/Cas9, neurosphere cells showed more differentiated cells, less self-renewal, and less proliferation capacity after irradiation [17]. The expression of NRF2 was associated with ferroptosis and resistance to apoptosis. For example, quiescin sulfhydryl oxidase 1 induced ferroptosis by suppressing NRF2 activity in EGFR-dependent tumor types [18]. Angiogenesis involved various hot molecules, such as HIF-1*α*, cytokines, VEGF, and extracellular matrix (ECM) remodelers [19]. The expression of NRF2 was associated with sustained angiogenesis. For example, NRF2 deficiency reduced protein levels of PDGF, HIF-1*α*, VEGF, angiogenin, and angiopoietin, which could result in significantly impaired survival and angiogenic capacity of endothelial cells [20]. However, NRF2 also has direct and indirect roles in suppressing cancers. Many studies demonstrated that Nrf2−/− mice had persistent inflammation and were able to avoid immune destruction [21].

NK cell recruitment was regulated by IL-17D in response to anticancer immune. NRF2 was reported to promote tumor rejection by initiating ARE at the promoter of IL-17D [22]. Inflammatory microenvironment in cancer contained immune suppression, such as Tregs and myeloid-derived suppressor cells (MDSCs), which promoted tumor inflammation and metastasis. MDSCs were higher in Nrf2−/− mice compared to their wild-type counterparts [23]. Since NRF2 can regulate lots of downstream genes to promote or inhibit cancers, NRF2 might be an oncogene or a tumor suppressor gene [16]. Beyond redox-regulating capacities of NRF2, more new functions were identified for NRF2, including proliferation, autophagy, energetic metabolism, cell stemness, amino acid metabolism, immune microenvironment, DNA repair, iron metabolism, proteasomal degradation, mitochondrial physiology, and drug metabolism [24].

In terms of prognostic biomarker or therapeutic target, NRF2 showed optimistic scenario into the cancer clinic. This study aimed to systematically characterize the molecular alterations, clinical relevance, and biological processes of NRF2 pathway-related genes across 33 cancer types. The widespread genetic alterations of NRF2 pathway-related genes, including expressions, mutations, and copy number variations (CNVs), demonstrated the complex mechanisms regulating tumorigenesis and development. The significant correlations between the expression levels of NRF2 pathway-related genes and tumor mutation burden (TMB), microsatellite instability score (MSI), immune phenotype, immune score, immune cell, cancer stemness index (RNAss), and drug sensitivity demonstrated that NRF2 had crosstalk with other molecules. The clinical relevance analysis showed that the NRF2 pathway-related genes could be potential biomarkers of cancers. Furthermore, the prognosis models were constructed for representative cancers, including lung squamous carcinoma (LUSC), breast cancer (BRAC), and stomach cancer (STAD).

## 2. Materials and Methods

The methods used in this section were mainly referred to our previous publication [25].

### 2.1. Collection of NRF2 Pathway-Related Genes

A total of 24 NRF2 pathway-related genes were collected with Ingenuity Pathway Analysis (IPA) (https://digitalinsights.qiagen.com/products-overview/discovery-insights-portfolio/analysis-and-visualization/qiagen-ipa/), including ATF4, BACH1, CREBBP, CUL3, EIF2AK3, EP300, FOS, FOSL1, GSK3B, JUN, KEAP1, MAF, MAFF, MAFG, MAFK, MAPK1, MAPK3, MAPK7, MAPK8, MAPK9, NRF2, PIK3CA, PRRT2, and RIT1 (Supplementary Table 1).

### 2.2. Genome-Wide Omics Data across 33 Cancer Types

Genome-wide omics data were based on UCSC Xena datasets (https://xenabrowser.net/datapages/). A total of 33 different cancer types were analyzed, including adrenocortical carcinoma (ACC), bladder urothelial carcinoma (BLCA), breast cancer (BRCA), cervical squamous cell carcinoma and endocervical adenocarcinoma (CESC), cholangiocarcinoma (CHOL), colon adenocarcinoma (COAD), lymphoid neoplasm diffuse large B cell lymphoma (DLBC), esophageal carcinoma (ESCA), glioblastoma multiforme (GBM), head and neck squamous carcinoma (HNSC), kidney chromophobe (KICH), kidney renal clear cell carcinoma (KIRC), kidney renal papillary cell carcinoma (KIRP), acute myeloid leukemia (LAML), brain lower grade glioma (LGG), liver hepatocellular carcinoma (LIHC), lung adenocarcinoma (LUAD), lung squamous cell carcinoma (LUSC), mesothelioma (MESO), ovarian serous cystadenocarcinoma (OV), pancreatic adenocarcinoma (PAAD), pheochromocytoma and paraganglioma (PCPG), prostate adenocarcinoma (PRAD), rectum adenocarcinoma (READ), sarcoma (SARC), skin cutaneous melanoma (SKCM), stomach adenocarcinoma (STAD), testicular germ cell tumors (TGCT), thyroid carcinoma (THCA), thymoma (THYM), uterine corpus endometrial carcinoma (UCEC), uterine carcinosarcoma (UCS), and uveal melanoma (UVM). Genome-wide omics data of NRF2 pathway-related genes included gene expression RNAseq (HTSeq-FPKM GDC Hub), somatic mutation (VarScan2 Variant Aggregation and Masking, Supplementary Table 2), copy number variant (CNV) (GISTIC focal score by gene GDC Hub), clinical characteristics (curated clinical data by Pan-Cancer Atlas Hub), immune phenotype (immune subtype by Pan-Cancer Atlas Hub), and cancer stemness index (stemness score-RNA based on Pan-Cancer Atlas Hub). The Maftools R package (https://www.bioconductor.org/packages/release/bioc/html/maftools.html) was used to calculate the distribution of tumor mutation burden (TMB) according to somatic mutation data (Supplementary Table 3), which also generated waterfall plots of mutation genes. Microsatellite instability (MIS) scores were obtained from published data (PMID: 30211344 and Supplementary Table 4) based on TCGA Research Network (http://cancergenome.nih.gov/).

### 2.3. Differential Expression Analysis of Nrf2 Pathway-Related Genes between Tumor and Normal Tissues across 33 Cancer Types

The ggpubr R package (https://rpkgs.datanovia.com/ggpubr/) was used to determine differentially expressed genes (DEGs) of NRF2 pathway-related genes between tumor and normal tissues across 33 cancer types, with statistical significance (adjusted *p* value < 0.05) (Supplementary Table 5 and Supplementary Figure 1). The Wilcoxon test was used to estimate the significance of gene expression alterations. The *p* value was adjusted with the Benjamini-Hochberg multiple testing correction. The heatmap of DEGs was plotted by pheatmap R packages (https://www.rdocumentation.org/packages/pheatmap/versions/1.0.12/topics/pheatmap).

### 2.4. The Correlations between Expression of NRF2 Pathway-Related Genes and TBM or MIS or CNV

The Corrplot R package (https://cran.r-project.org/web/packages/corrplot/vignettes/corrplot-intro.html) was used to perform the correlation analysis between the NRF2 pathway-related gene expression and TBM or MIS with method of spearman (*p* < 0.05). The fmsb R package (https://cran.rproject.org/web/packages/fmsb/index.html) was used to plot the correlation between the NRF2 pathway-related gene expression and TBM or MIS by radar chart. The correlation between the NRF2 pathway-related gene expression and CNV was calculated by the Kruskal test (*p* < 0.05), and boxplots were plotted by the barplot R package (https://www.rdocumentation.org/packages/graphics/versions/3.6.2/topics/barplot).

### 2.5. The Associations between the NRF2 Pathway-Related Gene Expressions and Clinical Features

The samples were divided into high- and low-expression groups of NRF2 pathway-related genes by median value of each gene across 33 cancer types. The Kaplan-Meier method based on the survminer R package (https://cran.r-project.org/web/packages/survminer/index.html) was used for overall survival analysis, which was compared to the log-rank test, with statistical significance of *p* < 0.05. The Cox regression analysis was also performed with the survival R package (https://www.rdocumentation.org/packages/survival/versions/3.2-3) to select survival-associated NRF2 pathway-related genes (Supplementary Table 6). The hazard ratio was calculated for the Cox proportional hazard regression models. Further, the associations between clinical characteristics (pathologic stage, including stages I, II, III, and IV) and NRF2 pathway-related gene expressions were analyzed across 33 cancer types.

### 2.6. The Expressions of NRF2 Pathway-Related Genes among Different Immune Phenotypes across 33 Cancer Types

Samples of TCGA Pan-Cancer data were divided into six clusters, including wound healing (Immune C1), IFN-gamma dominant (Immune C2), inflammatory (Immune C3), lymphocyte depleted (Immune C4), immunologically quiet (Immune C5), and TGF-beta dominant (Immune C6) based on immune model subtypes. The different expressions of NRF2 pathway-related genes among different immune model subtypes were analyzed with the Kruskal test (*p* < 0.05) and plotted with the ggplot2 R package (https://cran.r-project.org/web/packages/ggplot2/index.html).

### 2.7. Estimation of Immune-Related Scores and Infiltrating Cells across 33 Cancer Types

The presence of infiltrating stromal and immune cells in tumor tissues was predicted with ESTIMATE R package (https://bioinformatics.mdanderson.org/estimate/rpackage.html) that estimated stromal and immune cells in malignant tumor tissues with gene expression data. The ESTIMATE algorithm was based on ssGSEA analysis, which generated ImmuneScore representing the infiltration of immune cells in tumor tissue, StromalScore capturing the presence of stroma in tumor tissue, and ESTIMATEScore (Supplementary Table 7). These three scores were positively correlated with the corresponding ratio of immune cells, stromal cells, and the sum of both, respectively, which further mean that the higher score reflects the larger ratio of the corresponding component in tumor microenvironment. The Corrplot R package (https://cran.r-project.org/web/packages/corrplot/vignettes/corrplot-intro.html) was used to perform the correlation analysis between the NRF2 pathway-related gene expressions and ImmuneScore, StromalScore, or ESTIMATEScore with method of Spearman (*p* < 0.05) (Supplementary Figure 2).

### 2.8. The Proportion of Immune Cells across 33 Cancer Types Based on CIBERSORT Method

To quantify the proportion of immune cells across 33 cancer types, the CIBERSORT algorithm and the LM22 gene signature were used, which allows for highly sensitive and specific discrimination of 22 human immune cell phenotypes. Gene expression profiles were prepared with standard annotation files, and data were uploaded to the CIBERSORT web portal (http://cibersort.stanford.edu/), with the algorithm based on the LM22 signature and 1,000 permutations (Supplementary Table 8). The Corrplot R package (https://cran.r-project.org/web/packages/corrplot/vignettes/corrplot-intro.html) was used to perform the correlation analysis between the NRF2 pathway-related gene expressions and different immune cells with the Spearman method (*p* < 0.05) (Supplementary Figure 2), including naïve B cells, memory B cells, plasma cells, CD8^+^ T cells, naïve CD4^+^ T cells, resting memory CD4^+^ T cells, activated memory CD4^+^ T cells, follicular helper T cells, regulatory T cells (Tregs), gamma delta T cells, resting NK cells, activated NK cells, monocytes, macrophages M0, macrophages M1, macrophages M2, resting dendritic cells, activated dendritic cells, resting mast cells, activated mast cells, eosinophils, and neutrophils.

### 2.9. The Associations between the NRF2 Pathway-Related Gene Expressions and Cancer Stemness or Drug Sensitivity

RNA expression-based (all set of available genes) stemness scores (RNAss) were derived from the stemness group based on epigenetically regulated RNA expressions of 103 stemness-related genes. The Corrplot R package (https://cran.r-project.org/web/packages/corrplot/vignettes/corrplot-intro.html) was used to perform the correlation analysis between the NRF2 pathway-related gene expressions and RNAss with the Spearman method (*p* < 0.05). The NCI-60 cell line panel was developed as an anticancer drug efficacy screen by the developmental therapeutics program (DTP) of the US National Cancer Institute (NCI). Many thousands of compounds have been applied to the NCI-60. CellMiner (https://discover.nci.nih.gov/cellminer/) was a web-based suite of genomic and pharmacologic tools to explore transcript and drug patterns in the NCI-60 cell line set. The associations between the NRF2 pathway-related gene expressions and drug sensitivity were performed by the Corrplot R package with the Spearman method (*p* < 0.05) based on the corresponding data from CellMiner (Supplementary Table 9).

### 2.10. Construction of Prognostic Models and Their Associations with Clinical Characteristics

To compare similarities and differences of prognostic models among different tumors, the cancers of the respiratory system (LUSC), the gynecological system (BRAC), and the digestive system (STAD) were selected to construct prognostic models as representative examples (Supplementary Table 10). The total dataset of each cancer was randomly divided into training and testing sets using R package of caret (classification and regression training) with proportionate-stratified random sampling (https://cran.r-project.org/web/packages/caret/index.html). The samples in the training set were analyzed with the multivariate Cox regression analysis (steps forward) to calculate riskscores and construct prognostic models. The equation of riskscore was Riskscore =  _∑_^*n*^_*k*−*l*_Exp_*k*_∗*e*^HR^_*k*_, where *n* was the number of prognostic genes, Exp_*k*_ was the expression value of the prognostic genes, and *e*^HR^_*k*_ was the estimated regression coefficient of genes in the multivariate Cox regression analysis [26]. The samples of testing dataset were divided into high- and low-risk groups according to the formula of riskscores derived from the training set. After removing patients whose survival time was NA, the Kaplan-Meier (KM) survival analysis was used to plot survival curves, and compared with the log-rank test, respectively. In additional, receiver operating characteristic (ROC) curve was used to test classification measurement based on riskscore in the total dataset (including training and testing sets). The corresponding clinical characteristics of LUCS, BRAC, and STAD were obtained from TCGA (https://portal.gdc.cancer.gov/). The multivariate Cox regression model was used to analyze whether riskscore could be independent risk factor in cancer. Clinic correlation between high- and low-risk score groups was performed with pheatmap R package (https://www.rdocumentation.org/packages/pheatmap/versions/1.0.12).

## 3. Results

### 3.1. Widespread Genetic Alterations of NRF2 Pathway-Related Genes between Tumor and Normal Groups across Different Cancer Types

**Figure 1:**
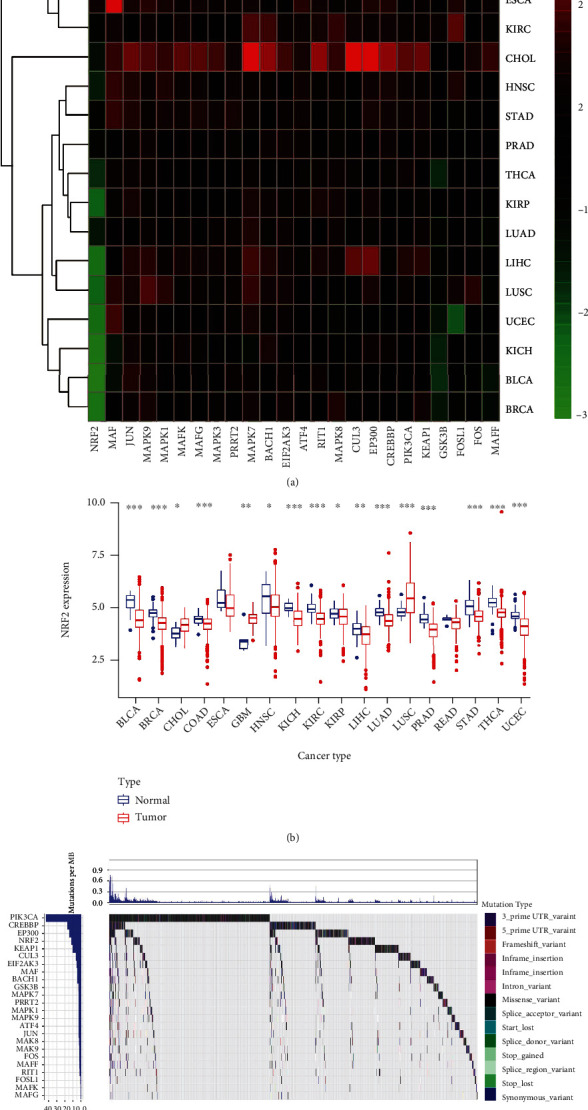
Pan-cancer analysis of expression and mutation alterations of NRF2 pathway-related genes. (a) The NRF2 pathway-related gene expression alterations across 18 cancer types. (b) The expression of NRF2 between tumor and normal tissues across 18 cancer types. (c) The mutation frequency of NRF2 pathway-related genes across 33 cancer types. ^∗^*p* < 0.05, ^∗∗^*p* < 0.01, and ^∗∗∗^*p* < 0.001.

The heatmap of differently expressed NRF2 pathway-related genes showed that widespread genetic alterations generated between tumor and normal groups across 18 cancer types, including BLCA, BRCA, CHOL, COAD, ESCA, GBM, HNSC, KICH, KIRC, KIRP, LIHC, LUAD, LUSC, PRAD, READ, STAD, THCA, and UCEC (Figure 1(a), Supplementary Figure 1, and Supplementary Table 5). The heatmap showed that most of the NRF2 pathway-related genes were significantly upregulated between tumor and normal groups across different cancer types. For example, KEAP1 was significantly upregulated in cancers BLCA, BRCA, CHOL, COAD, ESCA, GBM, HNSC, KIRC, LIHC, LUAD, LUSC, PRAD, READ, and THCA (Supplementary Figure 1). However, FOS and JUN were significantly downregulated in most of cancer compared to corresponding normal tissues. For example, FOS was significantly downregulated in cancers BLCA, BRCA, CHOL, COAD, HNSC, KICH, KIRC, KIRP, LIHC, LUAD, LUSC, PRAD, READ, STAD, THCA, and UCEC. JUN was significantly downregulated in cancers BLCA, BRCA, KICH, KIRP, LIHC, LUAD, LUSC, PRAD, STAD, THCA, and UCEC (Supplementary Figure 1). Here, the NRF2 expression was taken as an example to show the differential expressions between tumor and normal groups across 18 cancer types (Figure 1(b)). NRF2 was significantly upregulated in cancers GBM and LUSC and downregulated in cancers BLCA, BRCA, CHOL, COAD, HNSC, KICH, KIRC, KIRP, LIHC, LUAD, PRAD, STAD, THCA, and UCEC.

### 3.2. The Overall Average Mutation Frequency of NRF2 Pathway-Related Genes across 33 Cancer Types

The overall average mutation frequencies of NRF2 pathway-related genes ranged from 0.01 to 50%. The types of mutations included 3′ prime UTR variant, 5′ prime UTR variant, coding sequence variant, downstream gene variant, frame shift variant, inframe deletion, inframe insertion, intron variant, missense variant, splice acceptor variant, splice donor variant, splice region variant, start lost, stop gained, stop lost, stop retained variant, synonymous variant, and upstream gene variant. The waterfall map of mutation distribution showed the detailed mutation status of NRF2 pathway-related genes from high to low percentage, PIK3CA, CREBBP, EP300, NRF2, KEAP1, CUL3, EIF2AK3, MAF, BACH1, MAPK1, MAPK7, PRRT2, MAPK9, GSK3B, MAPK8, JUN, ATF4, FOS, MAPK3, MAFF, RIT1, FOSL1, MAFK, and MAFG (Figure 1(c) and Supplementary Table 2).

### 3.3. The Significant Associations between the NRF2 Pathway-Related Gene Expressions and TMB or MSI or CNV

**Figure 2:**
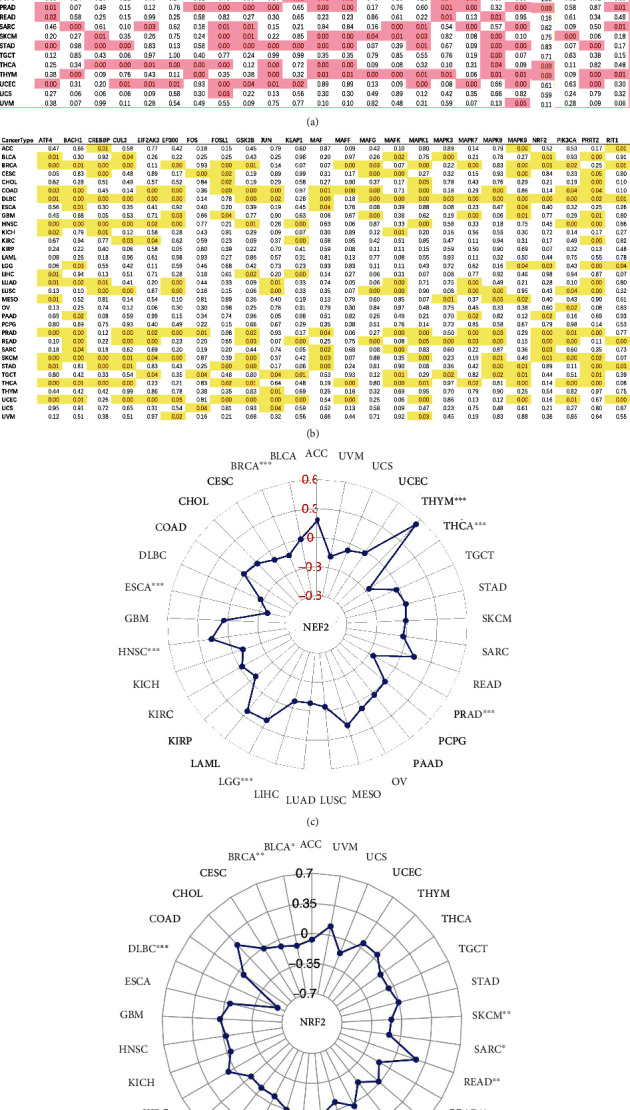
Pan-cancer analysis of associations of the NRF2 pathway-related genes with TMB and MSI. (a) The associations of the NRF2 pathway-related gene expressions with TMB across 33 cancer types. (b) The association of the NRF2 expression with TMB across 33 cancer types. (c) The associations of the NRF2 pathway-related gene expressions with MSI across 33 cancer types. (d) The association of the NRF2 expression with MSI across 33 cancer types. ^∗^*p* < 0.05, ^∗∗^*p* < 0.01, and ^∗∗∗^*p* < 0.001.

TMB as a new biomarker in cancers has received increasing attention in recent years. The correlations between the NRF2 pathway-related gene expressions and TMB score (Supplementary Table 3) were evaluated across 33 cancer types (Figure 2(a)). Multiple genes were significantly correlated with TMB score in different cancer types, such as ATF4, FOSL1, GSK3B, JUN, KEAP1, MAF, MAFF, MAPK7, MAPK8, and RIT1. Here, NRF2 was taken as an example to show the radar plot of NRF2 expression and TMB score across different cancers. The expression of NRF2 showed negative correlation in BRCA, ESCA, THCA, and PRAD and positive correlation in HNSC, LGG, and THYM (Figure 2(c)).

MSI as a new biomarker in cancers has also received increasing attention in recent years. The correlations between the NRF2 pathway-related gene expressions and MSI score (Supplementary Table 4) were evaluated across 33 cancer types (Figure 2(b)). Multiple genes were significantly correlated with MSI score in different cancer types, such as ATF4, BACH1, CREBBP, CUL3, EP300, GSK3B, MAPK9, NRF2, PIK3CA, and PRRT2. Here, NRF2 was taken as an example to show the radar plot of NRF2 expression and MSI score across different cancers. The expression of NRF2 showed negative correlation in DLBC, BLCA, LGG, PAAD, SARC, SKCM, BRCA, and PRAD and positive correlation in READ (Figure 2(d)).

**Figure 3:**
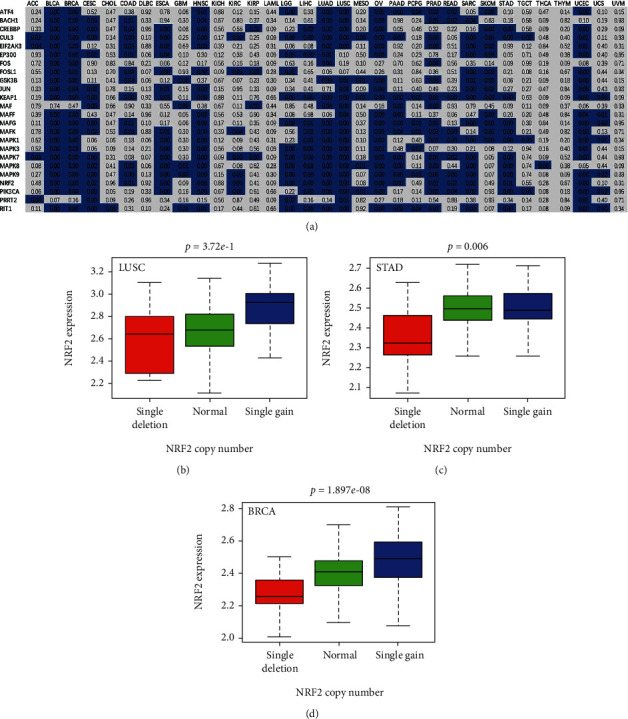
Pan-cancer analysis of associations of the NRF2 pathway-related gene expression with CNV. (a) The association of the NRF2 pathway-related gene expressions with CNV across 33 cancer types. (b) The correlation analysis between the NRF2 expression and CNV in LUSC. (c) The correlation analysis between the NRF2 expression and CNV in STAD. (d) The correlation analysis between the NRF2 expression and CNV in BRCA.

The associations between the NRF2 pathway-related gene expressions and CNV status (including single deletion, normal, and single gain) showed that multiple genes were significantly correlated with CNVs in many cancer types, such as BLCA, BRCA, CESC, HNSC, LGG, LUAD, LUSC, OV, PRAD, SARC, SKCM, and UCEC (Figure 3(a)). Here, NRF2 was taken as an example to show the boxplots of NRF2 expression and its CNV status in LUSC, STAD, and BRCA (Figures 3(b)–3(d)).

### 3.4. Clinical Relevance of NRF2 Pathway-Related Genes across Different Cancer Types

**Figure 4:**
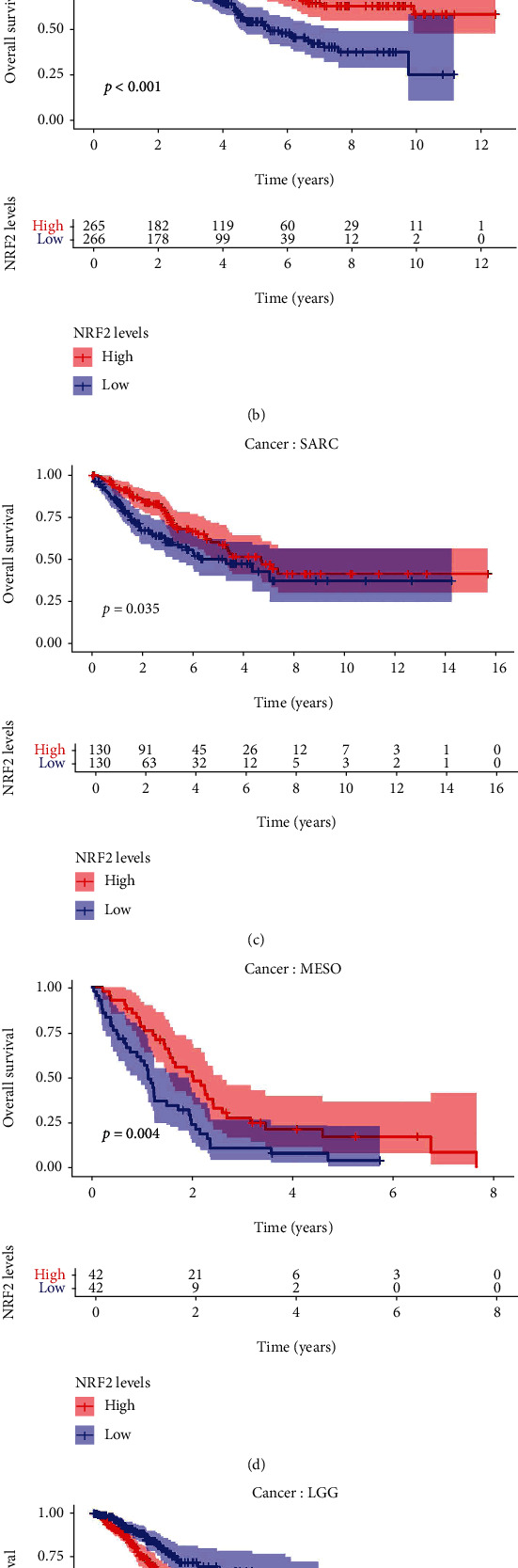
Overall survival of the NRF2 pathway-related genes across 33 cancer types. (a) Summary of the correlation between NRF2 pathway-related gene expressions and patient survival. Red represents a higher expression of the NRF2 pathway-related genes associated with worse survival, and green represents an association with better survival. Only *p* value < 0.05 was shown. (b) Kaplan-Meier survival curve of the NRF2 expression in KIRC. (c) Kaplan-Meier survival curve of the NRF2 expression in SARC. (d) Kaplan-Meier survival curve of the NRF2 expression in MESO. (e) Kaplan-Meier survival curve of the NRF2 expression in LGG. The patients grouped by global expression pattern of the NRF2 pathway-related genes based on median value.

The overall survival of the NRF2 pathway-related genes across 33 cancer types was analyzed, and many of them were significantly related to patient survival rates (Figure 4(a)). ACC, KIRC, and LGG showed a lot of significant results; for example, the high expressions of ATF4, FOSL1, EAP1, AFF, APK1, APK7, APK8, and RIT1 were significantly related to poor survival rate in ACC, but the high expressions of MAPK9 were significantly related to better survival rate in ACC. The high expressions of ATF4, FOSL1, AFK, and PRRT2 were significantly related to poor survival rate in KIRC, but the high expressions of CREBBP, CUL3, EP300, GSK3B, MAF, MAFG, MAPK1, MAPK3, MAPK8, MAPK9, NRF2, PIK3CA, and RIT1 were significantly related to better survival rate in KIRC. The high expressions of BACH1, EIF2AK3, FOSL1, JUN, MAF, MAFF, MAFK, NRF2, and RIT1 were significantly related to poor survival rate in LGG, but the high expressions of EP300, MAPK1, MAPK8, MAPK9, and PRRT2 were significantly related to better survival rate in LGG. Here, the Kaplan-Meier survival analysis curves of NRF2 were provided in KIRC (Figure 4(b)), SARC (Figure 4(c)), MESO (Figure 4(d)), and LGG (Figure 4(e)) as an example. The function of the same genes in different kinds of cancers might be different.

**Figure 5:**
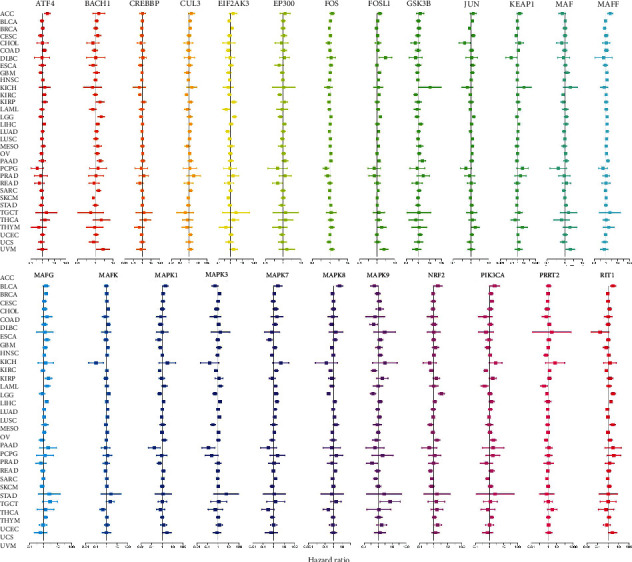
The distribution of hazard ratios of NRF2 pathway-related genes across different cancer types with the Cox regression survival analysis.

Furtherly, the expressions of the NRF2 pathway-related genes were acted as continuous variables to perform the Cox regression analysis for obtaining hazard ratio (HR) across 33 cancer types (Figure 5 and Supplementary Table 6). The results furtherly verified OS analysis, which indicated that the same gene in different cancer types would be risky factor (HR > 1) or protective factor (HR < 1). Meanwhile, a series of genes with similar function would also be identified as risky factor or protective factor in the same cancer. For example, NRF2 acted as risky factor in PAAD (HR = 1.710, *p* = 0.019) and LGG (HR = 3.510, *p* = 5.20E − 07) but acted as protective factor in SARC (HR = 0.586, *p* = 0.0001), MESO (HR = 0.558, *p* = 0.002), KIRC (HR = 0.663, *p* = 0.002), and SKCM (HR = 0.739, *p* = 0.004). For another example, FOSL1 (HR = 1.326, *p* = 6.81E − 06), GSK3B (HR = 1.326, *p* = 6.81E − 06), PRRT2 (HR = 1.520, *p* = 5.25E − 05), MAPK7 (HR = 1.722, *p* = 0.003), and ATF4 (HR = 1.458, *p* = 0.011) acted as risky factor in KIRC, but MAPK8 (HR = 0.663, *p* = 9.23E − 05), CUL3 (HR = 0.690, *p* = 2.64E − 05), EIF2AK3 (HR = 0.765, *p* = 2.64E − 05), MAPK9 (HR = 0.732, *p* = 0.001), CREBBP (HR = 0.729, *p* = 9.30E − 05), PIK3CA (HR = 0.728, *p* = 0.004), MAPK1 (HR = 0.873, *p* = 0.0001), MAPK3 (HR = 0.812, *p* = 0.0001), EP300 (HR = 0.834, *p* = 0.044), RIT1 (HR = 0.991, *p* = 0.012), and MAF (HR = 1.458, *p* = 0.012) acted as protective factor in KIRC (Supplementary Table 6).

**Figure 6:**
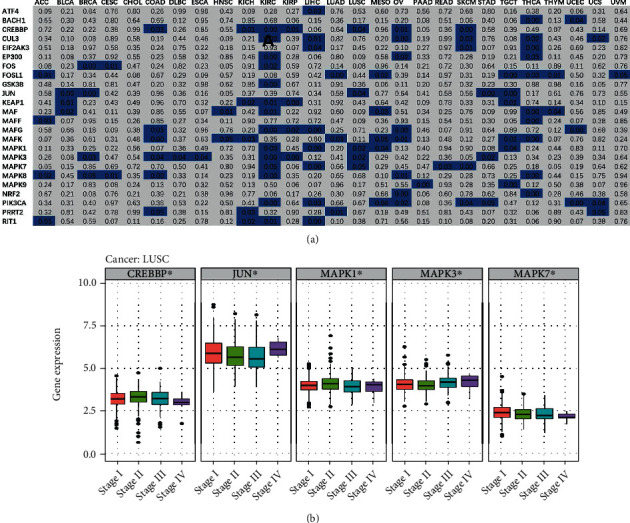
The clinical stage relevance of the NRF2 pathway-related genes across 33 cancer types. (a) Summary of the correlation between the NRF2 pathway-related gene expressions and clinical stages. Only *p* value < 0.05 was shown in blue. (b) Box plots showed the expression distribution of the NRF2 pathway-related genes among different clinical stages in LUSC. ^∗^*p* < 0.05, ^∗∗^*p* < 0.01, and ^∗∗∗^*p* < 0.001.

Moreover, the associations between pathologic stage (stages I, II, III, and IV) and NRF2 pathway-related genes were analyzed across 33 cancer types. The main associations are shown in KIRC and THCA (Figure 6(a)). For example, the expressions of MAPK8, MAPK3, CREBBP, MAFG, EP300, CUL3, GSK3B, PIK3CA, MAPK1, MAF, RIT1, KEAP1, FOS, and MAPK7 were significantly different among different stages in KIRC. The expressions of MAPK8, MAF, NRF2, MAFF, EIF2AK3, MAFK, BACH1, CUL3, FOSL1, and EP300 were significantly different among different stages in THCA. The expression of MAPK8 was significantly different among different stages in cancers KIRC, THCA, COAD, OV, CESC, and ACC. The expression of PIK3CA was significantly different among different stages in cancers UCEC, KIRC, OV, LIHC, SKCM, MESO, and UCS. Here, an example was taken to present the association between pathologic stages and expression of NRF2 pathway-related genes in LUSC, which showed the significant difference of MAPK1, MAPK3, JUN, CREBBP, and MAPK7 among different pathological stages (Figure 6(b)).

### 3.5. Association of NRF2 Pathway-Related Gene Expressions with Immune Microenvironment

**Figure 7:**
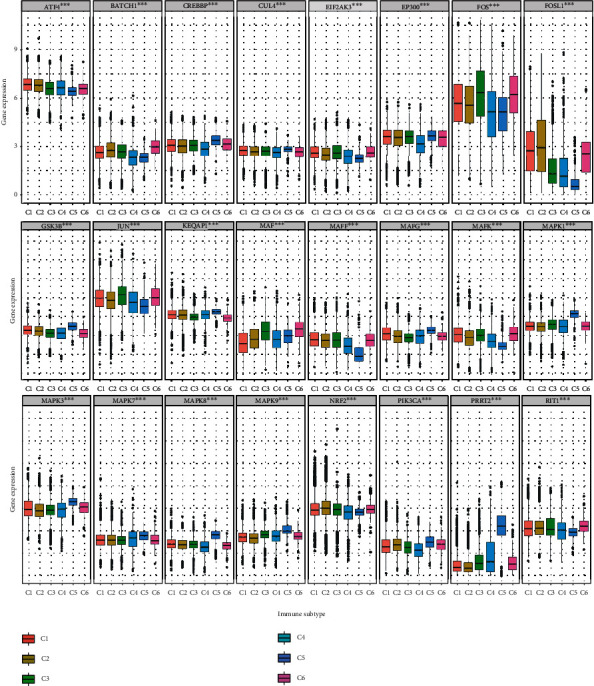
The expression difference of the NRF2 pathway-related genes among different immune subtypes across 33 cancer types.

**Figure 8:**
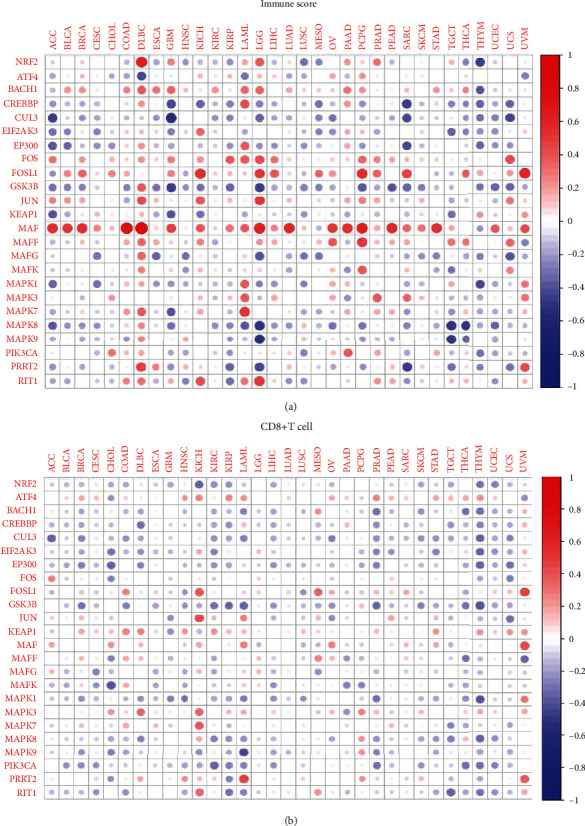
The relevance of the NRF2 pathway-related gene expressions and immune cells across 33 cancer types. (a) The correlation between NRF2 pathway-related gene expressions and ImmuneScore across 33 cancer types. (b) The correlation between the NRF2 pathway-related gene expressions and CD8^+^ T cells across 33 cancer types. Red dots represent positive correlation, and blue dots represent negative correlation. ^∗^*p* < 0.05, ^∗∗^*p* < 0.01, and ^∗∗∗^*p* < 0.001.

Immune microenvironment was an important part in the field of cancer evolution. To explore association of NRF2 pathway-related gene expressions with immune microenvironment, we analyzed the immune subtypes (C1, C2, C3, C4, and C5), immune-related scores (ImmuneScore, StromalScore, and ESTIMATEScore that are listed in Supplementary Table 7), and immune cells (naive B cells, memory B cells, plasma cells, CD8^+^ T cells, naive CD4^+^ T cells, resting memory CD4^+^ T cells, activated memory CD4^+^ T cells, follicular helper T cells, Tregs cells, gamma delta T cells, resting NK cells, activated NK cells, monocytes, macrophages M0, macrophages M1, macrophages M2, resting dendritic cells, activated dendritic cells, resting mast cells, activated mast cells, eosinophils, and neutrophils that are listed in Supplementary Table 8). The expressions of NRF2 pathway-related genes were significantly different among immune subtypes (Figure 7), which indicated that the NRF2 pathway might have crosstalk with immune system. Further studies showed that the same NRF2 pathway-related gene was significantly associated with immune-related scores in various cancers; especially, FOS, FOSL1 and MAF were positively associated with immune-related scores in various cancers, and CUL3, GSK3B, KEAP1, and MAPK8 were negatively associated with immune-related scores in various cancers. It was also clear that a series of NRF2 pathway-related genes were significantly related to immune-related scores in the same cancer; for example, BACH1, GSK3B, JUN, MAF, MAFF, MAFG, MAPK7, NRF2, PRRT2, and RIT1 were positively associated with immune-related scores in DLBC. BACH1, CREBBP, CUL3, EP300, MAPK1, NRF2, and PIK3CA were negatively associated with immune-related scores in THYM (Figure 8(a) and Supplementary Figure 2). The relationship between the NRF2 pathway-related gene expressions and CD8^+^ T cells generally showed negative relationship; for example, CUL3, EP300, GSK3B, MAPK1, MAPK9, and PIK3CA were significantly related to CD8^+^ T cell percentage in the multiple cancers (Figure 8(b)). The data of other immune cells are shown in Supplementary Figure 2, and the percentage of naive B cells were significantly related to many NRF2 pathway-related genes, such as ATF4, BACH1, CUL3, EP300, FOS, FOSL1, JUN, KEAP1, MAF, MAFG, MAFK, MAPF1, MAPK3, and MAPK9 in TGCT. In terms of the activated NK cells, they were generally negatively related to the NRF2 pathway-related gene expressions in multiple cancers, except for KEAP1 gene and THYM cancer, while the resting memory CD4^+^ T cells were generally positively related to the NRF2 pathway-related gene expressions in multiple cancers, except for ATF4, KEAP1, and MAPK3.

### 3.6. Association of NRF2 Pathway-Related Gene Expressions with RNAss and Drug Sensibility

**Figure 9:**
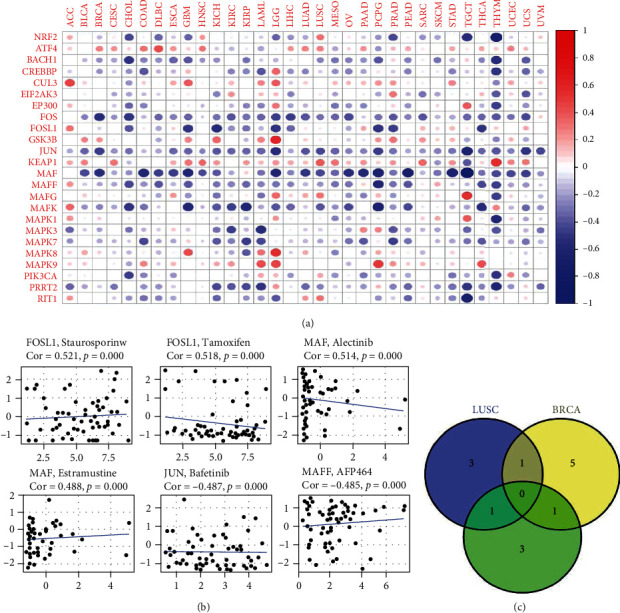
Pan-cancer analysis of associations of the NRF2 pathway-related genes with RNAss and drug sensitivity. (a) The correlation between the NRF2 pathway-related gene expressions and RNAss across 33 cancer types. Red dots represent positive correlation, and blue dots represent negative correlation. (b) The relevance of the NRF2 pathway-related gene expressions and drug sensitivity.

Therapy-induced stemness and nongenetic cancer cell plasticity in tumor strengthened cancer cells, and RNAss reflected stemness index. Most of the NRF2 pathway-related genes showed extensively negative relationships with RNAss; for example, the expressions of FOS, JUN, MAF, MAFK, and PRRT2 in various cancers were negatively related to RNAss. However, the expressions of CUL3, GSK3B, KEAP1, and MAPK9 showed positive relationship with RNAss in various cancers (Figure 9(a)). Moreover, the associations of NRF2 pathway-related genes with drug sensibility were explored. The drugs included tamoxifen, bafetinib, AFP464, raloxifene, SR16157, hypothemycin, dexrazoxane, fulvestrant, vemurafenib, vorinostat, dabrafenib, CUDC-305, cobimetinib (isomer 1), XK-469, dasatinib, selumetinib, dromostanolone propionate, oxaliplatin, LDK-378, denileukin diftitox ontak, triciribine phosphate, isotretinoin, amonafide, simvastatin, chelerythrine, XL-147, nelfinavir, mitomycin, lenvatinib, staurosporine, pyrazoloacridine, estramustine, 5-fluorodeoxyuridine 10mer, and alectinib (Figure 9(b) and Supplementary Table 9). Some NRF2 pathway-related genes showed negative association between drug sensibility, such as FOSL1 and tamoxifen, JUN and bafetinib, MAFF and AFP464, FOSL1 and raloxifene, FOSL1 and SR16157, JUN and hypothemycin, MAFF and dexrazoxane, and FOSL1 and fulvestrant (correlation coefficient < −0.45). Some NRF2 pathway-related genes showed positive association between drug sensibility, such as JUN and staurosporine, MAPK8 and pyrazoloacridine, FOSL1 and dasatinib, MAF and denileukin diftitox ontak, MAF and estramustine, MAPK3 and 5-fluoro deoxy uridine 10mer, MAF and alectinib, and FOSL1 and staurosporine (correlation coefficient > 0.45).

### 3.7. The Construction of NRF2 Pathway-Related Gene Signatures in Heterogeneous Tumors

To construct the NRF2 pathway-related gene signatures, TCGA LUSC dataset (*n* = 501), BRCA dataset (*n* = 1098), and STAD dataset (*n* = 375) were included in the multivariate Cox regression analysis. Finally, a prognostic model containing five genes (CUL3, EIF2AK3, JUN, MAFF, and MAPK3) was established to assess the prognosis of each patient in LUSC. A prognostic model containing seven genes (CREBBP, EP300, FOS, GSK3B, MAFF, MAFK, and PIK3CA) was established to assess the prognosis of each patient in BRCA. A prognostic model containing five genes (BACH1, KEAP1, MAFG, MAPK3, and PIK3CA) was established to assess the prognosis of each patient in LUSC. The detailed information of the multivariate Cox regressions in LUSD, BRCA, and STAD was shown (Table 1). The construction of NRF2-related gene signatures was different in heterogeneous tumors. The optimized signatures in LUSC, BRCA, and STAD contained different NRF2 pathway-related genes. Somehow, they share some same genes, and the weight was different (Figure 9(c)). For example, the correlation coefficient of MAFF was 0.35 in LUSC, while it was -0.30 in BRCA. The correlation coefficient of PIK3CA was 0.60 in BRCA, while it was 0.99 in STAD.

**Figure 10:**
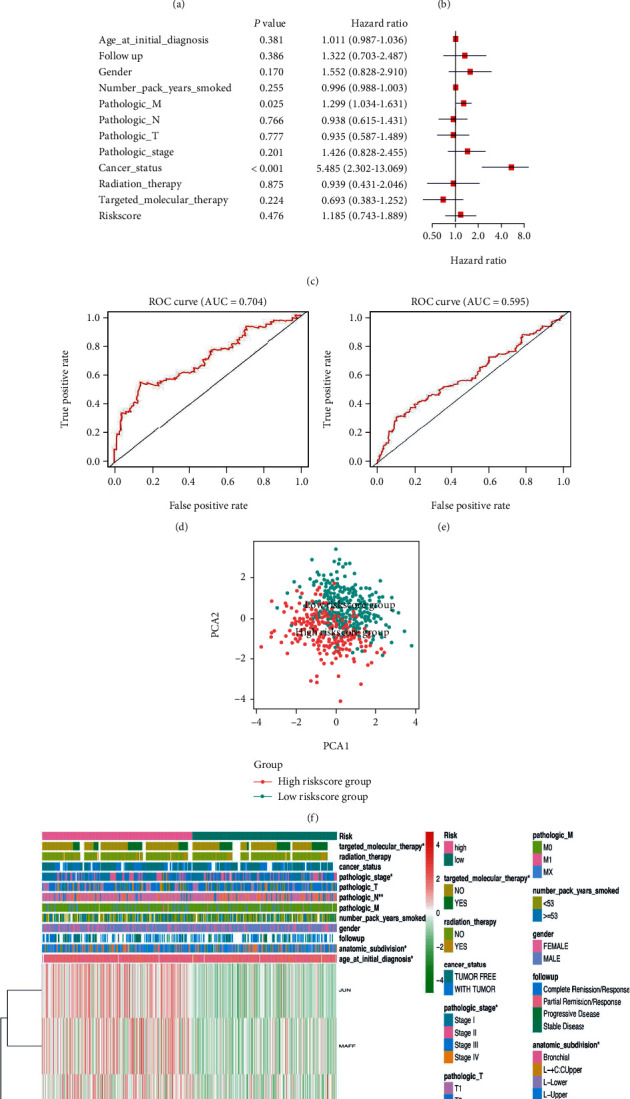
The Cox regression identified the prognostic model in LUSC. (a) Survival curve between high- and low-risk score groups in training set in LUSC. (b) Survival curve between high- and low-risk score groups in testing set in LUSC. (c) The multivariate Cox regression analysis of risk factors in LUSC. (d) ROC curve in training set based on risk score in LUSC. (e) ROC curve in the validation set based on risk score in LUSC. (f) PCA plot based on risk score in LUSC. (g) The heatmap of clinical correlation between high- and low-risk score groups in LUSC. ^∗^*p* < 0.05, ^∗∗^*p* < 0.01, and ^∗∗∗^*p* < 0.001.

**Figure 11:**
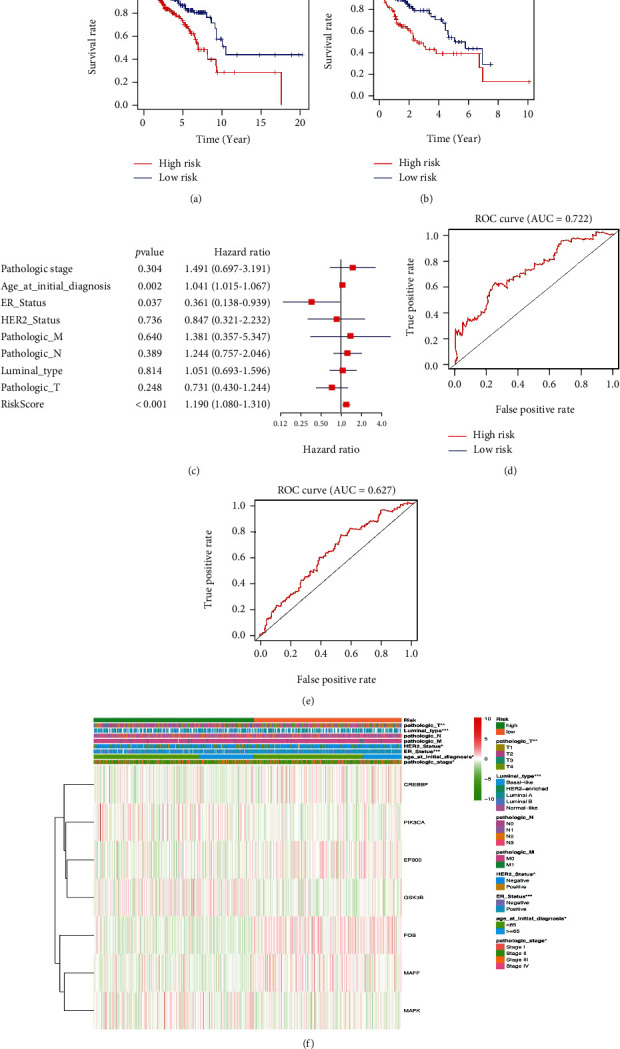
The Cox regression identified the prognostic model in BRCA. (a) Survival curve between high- and low-risk score groups in training set in BRCA. (b) Survival curve between high- and low-risk score groups in testing set in BRCA. (c) The multivariate Cox regression analysis of risk factors in BRCA. (d) ROC curve based on risk score in BRCA. (e) ROC curve in the validation set based on risk score in BRCA. (f) The heatmap of clinical correlation between high- and low-risk score groups in BRCA. ^∗^*p* < 0.05, ^∗∗^*p* < 0.01, and ^∗∗∗^*p* < 0.001.

**Figure 12:**
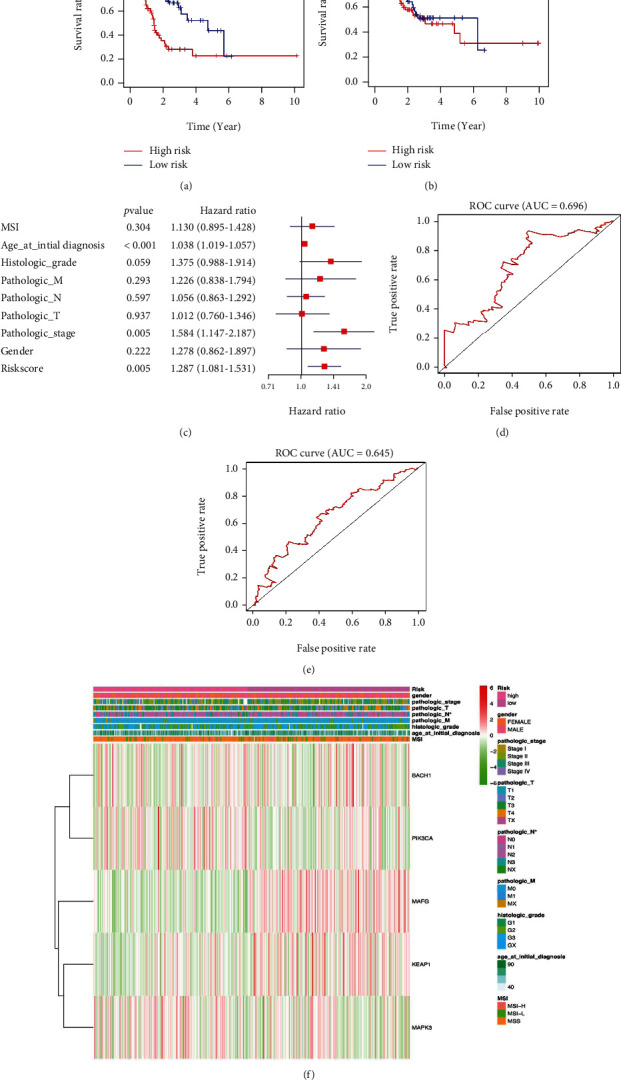
The Cox regression identified the prognostic model in STAD. (a) Survival curve between high- and low-risk score groups in training set in STAD. (b) Survival curve between high- and low-risk score groups in testing set in STAD. (c) The multivariate Cox regression analysis of risk factors in STAD. (d) ROC curve based on risk score in STAD. (e) ROC curve in the validation set based on risk score in STAD. (f) The heatmap of clinical correlation between high- and low-risk score groups in STAD. ^∗^*p* < 0.05, ^∗∗^*p* < 0.01, and ^∗∗∗^*p* < 0.001.

The total datasets were divided into training sets and validation sets in LUSC, BRCA, and STAD, respectively (Supplementary Table 10). The KM plots were used to evaluate the performance of NRF2 pathway-related gene signatures in predicting the outcome of cancer patients. For both training sets and validation sets, the OS between low- and high-risk groups classified by risk score of the constructed prognostic model was significantly different in LUSC (Figures 10(a) and 10(b)), BRCA (Figures 11(a) and 11(b)), and STAD (Figures 12(a) and 12(b)). Then, the performances of prognostic models were further assessed with other common prognostic factors by the multivariate Cox regression analysis. Five-gene signature cannot be used as independent prognostic factor in LUCS (*p* = 0.476, Figure 10(c)). Seven-gene signature can be used as independent prognostic factor in BRCA (*p* < 0.001, Figure 11(c)). Five-gene signature can be used as independent prognostic factor in STAD (*p* = 0.005, Figure 12(c)). ROC curves were used to evaluate the performance of gene signatures, and the value of AUC was more than 0.50 in the training set in LUSC (Figure 10(d)), BRCA (Figure 11(d)), and STAD (Figure 12(d)), respectively. The value of AUC in the validation set was also more than 0.50 in LUSC (Figure 10(e)), BRCA (Figure 11(e)), and STAD (Figure 12(e)). Additionally, all LUSC samples can be well divided into two groups (high risk and low risk) according to risk score based on verification of PCA (Figure 10(f)). These results indicated that the constructed prognostic models were robust in predicting the outcome of patients.

The heatmap showed that the risk groups had a significant association with clinical features in LUSC, including anatomic subdivision, age at initial diagnosis, pathologic N stage, targeted molecular therapy, and pathologic stage (Figure 10(g) and Supplementary Table 11). The heatmap showed that the risk groups had a significant association with clinical features in BRCA, including age at initial diagnosis, pathologic stage, ER status, HER2 status, luminal type, and pathologic T stage (Figure 11(f) and Supplementary Table 11). The heatmap showed that the risk groups had a significant association with clinical features in STAD, including pathologic N stage (Figure 12(f) and Supplementary Table 11).

## 4. Discussion

Oxidative stress and the multifaceted results of oxidative damage were important contributors to the initiation, promotion, and progression in various cancers. The impacts of oxidative stress on the body were widespread, including DNA, RNA, protein, and lipid. Oxidative DNA might be more vulnerable to be modified (methylation) or developed mutation (point mutations, insertions, deletions, or chromosomal translocations), which may lead to the activation of tumor suppressor gene or oncogene [27]. Oxidative RNAs, including protein-coding RNAs and noncoding RNAs, also induced modifications of bases and ribose, strand break, and base excision. Oxidative RNA adducts (8-hydroxyadenine, 5-hydroxycitosine, and 8-oxoguanosine) could potentially result in incorrect modification of gene expression, inhibition of DNA repair enzymes, polymorphisms of antioxidant enzymes, and errors in protein synthesis [28]. Oxidative proteins mainly showed damaged amino acids, changed enzyme activities, errors in protein structure, and aberrant spectra of posttranslational modifications. The development and application of new techniques have been used to identify and quantify protein oxidation [29]. In addition, ROS can also react with lipids (polyunsaturated fatty acids) resulting in lipid peroxidation. The product of lipid peroxidation, such as malondialdehyde, was higher in a variety of cancers, including cervical, ovarian, brain, breast, prostate, lung, chronic lymphocytic leukemia, bladder, renal, and thyroid cancer [30]. Nrf2 as the heart of oxidative stress response could quickly be translocated from cytoplasm into the nucleus in response to oxidative stress. The accumulated evidence reported that the Nrf2 signaling pathway involved in multiple hallmarks of cancer, including tissue invasion and metastasis, insensitivity to antigrowth signals, altered redox homeostasis, limitless replicative potential, avoiding immune destruction, sustained proliferative signaling, genome instability, sustained angiogenesis, proteotoxic stress, resistance to apoptosis, metabolite programming, and tumor-promoting inflammation [16].

This study selected Nrf2 and Nrf2 pathway-related genes to do a systematic analysis in pan-cancer, including ATF4, BACH1, CREBBP, CUL3, EIF2AK3, EP300, FOS, FOSL1, GSK3B, JUN, KEAP1, MAF, MAFF, MAFG, MAFK, MAPK1, MAPK3, MAPK7, MAPK8, MAPK9, NRF2, PIK3CA, PRRT2, and RIT1. These genes directly interacted with Nrf2 in Nrf2 signaling pathway, which is involved in oxidative stress with Nrf2 based on some clinical and basic research applications [31]. KEAP1 interacted with Nrf2 in a redox-sensitive manner, whose dissociation could activate Nrf2 to transfer from cytoplasm to the nucleus, contributing to cancer progression. Using a mutant K-ras/p53 mouse model to study the function of Keap1 deletion, the formation of invasive cholangiocarcinoma occurred and genetic sequencing identified a number of upregulated Nrf2 target genes [32]. Cul3 gene, as the core component of an E3 ubiquitin ligase complex, played a critical role in the ubiquitylation-mediated protein degradation. Under normal conditions, Nrf2 was maintained at very low concentrations by interacting with Keap1 and the Cul3 E3 ligase. Using siRNA to silence Cul3 in breast cancer cells, microarray analysis revealed that the expressions of oxidative stress downstream genes (AKR1C1, UGDH, TXN, GCL, and NQO1) were overexpressed at least 2-fold. The upregulation of Cul3 could deplete Nrf2 in breast cancer and was associated with sensitivity to oxidative stress, carcinogens, and chemotherapy [33]. MAF was a DNA-binding protein that acts as a homodimer or a heterodimer, containing leucine zipper domain. Nrf2 was one of the binding partners of small MAF proteins, and the Nrf2/small Maf DNA-binding complexes help to recognize stress response element sites in response to oxidative stress [34]. PIK3CA protein represented the catalytic subunit, which used ATP to phosphorylate phosphatidylinositol, phosphatidylinositol 4-phosphate and phosphatidylinositol 4,5-bisphosphate. PIK3CA and AKT (PI3K/Akt) pathway was one of the most frequently dysregulated pathways in metabolic rewiring and ROS metabolism. The hyperactive PI3K/Akt signaling generated metabolic byproducts to stimulate ROS production, and further research focusing on the association of PIK3CA-mutant tumors and redox homeostasis could contribute suppress tumor growth and overcome drug resistance [35]. The MAP kinase family acted as an integration point for multiple cellular processes and targeted many specific transcription factors in response to various cellular stressors (oxidative stress, DNA damage, and endoplasmic reticulum stress) [36]. Studies demonstrated that the MAP kinases, such as ERK1/2, JNK, and p38, were activated in response to oxidative stress. The process often involved in reaction networks, not just one MAP kinase but a series of interlinked enzymes that initiated a cascade of signals. Furthermore, the redox-activated MAP kinase family also had crosstalk with other effector pathways, for example, PI3K interaction [37]. The heatmap of the Nrf2 pathway-related genes across 18 cancer types showed the significant overexpressions of some genes, including FOSL1 in COAD, ESCA, and READ, MAFG in CHOL, MAPK3 in BRCA, and RIT1 in CHOL (Log_2_^foldchange^ > 1.5) and the significant downregulations of some genes, including FOS in BLCA, BRCA, HNSC, KIRC, KIRP, LIHC, LUAD, LUSC, THCA, and UCEC, MAF in UCEC, MAPK9 in GBM, PRRT2 in GBM, and JUN in BLCA, BRCA, KICH, THCA, and UCEC (Log_2_^foldchange^ < −1.5). Furthermore, to investigate clinical characteristics that were associated with the NRF2 pathway-related genes, many dysregulated genes were significantly related to OS and pathological stages. Some highly consistent results might be important; for example, the overexpressed FOSL1 indicated poor survival in ESCA patients. The underexpressed PRRT2 indicated good survival in GBM patients. Some genes played poor-prognostic factors in cancers, for example, ATF4 in ACC, BACH1 in LGG and UVM, and FOSL1 in DLBC and UVM. Some genes played well-prognostic factors in cancers, for example, MAFK in KICH, MAPK3 in PCPG and MESC, NRF2 in UVM, and PIK3CA in KIRC. This study provided dysregulation of the NRF2 pathway-related genes in various cancers with corresponding clinical features. The highly consistent results might be potential biomarker in cancers.

This study not only analyzed the expressions of the Nrf2 pathway-related genes but also focused on the mutation data. PIK3CA was one of the most commonly mutated genes across different cancer types, and this study also obtained consistent results with the published reports. When compared to the wild-type PIK3CA protein, the mutation type prompted transformation and tumorigenicity. Efforts to target new hotspot mutations in PIK3CA were on the rise, which induced PI3K-based cancer drugs continuing to emerge in the clinical trials [38]. Aberrant expression of PIK3CA was recognized as the major target genes involved in oxidative stress-induced carcinogenesis. It might be a potential method to study the pathophysiologic roles of somatic mutations and develop highly specific agents in response to oxidative stress. Comprehensive genomic analyses also identified somatic mutations or other alterations in the NRF2 and KEAP1genes. The somatic mutations in KEAP1, NRF2, and other mechanisms increasing ROS could disrupt KEAP1-NRF2 binding sites and aberrantly activate NRF2 [39]. The EP300-G211S mutation was exclusively identified in the triple-negative breast cancer patients, and its presence was significantly associated with overall other pathological somatic mutational patterns. During long-term follow-up of triple-negative breast cancer patients, EP300-G211S was proved to be a protective factor, which decreased breast cancer-specific mortality and predicted a lower risk for relapses [40]. GSK3B encoded a serine-threonine kinase, belonging to the glycogen synthase kinase subfamily. It was involved in several cancers, including prostate cancer, leukemia, hepatic carcinoma, cholangiocarcinoma, melanoma, and breast cancer. GSK3B played a role in oxidative stress and could be a possible therapeutic target of synthetic inhibitors in many diseases. The mutation of various phosphorylation sites in GSK3B was identified in cell nuclei of prostate cancer tissue samples and contributed to prostate cancer progression by increasing protein kinase B/Akt and Akt activity [41]. Some high frequency of mutations, such as PIK3CA, CREBBP, EP300, KEAP1, and CUL3, should be focused on and especially PIK3CA mutations that have been reported in many published data [38]. Those mutations were expected to become new targets for cancer biotherapy.

This study also explored the association between the NRF2 pathway-related gene expressions and TMB, MSI, or CNV. TMB reflected cancer mutation quantity, which was predictive of survival benefit in patients with various cancers, and was regarded as the most prevalent biomarker to predict immunotherapy. EP300 as one of frequently mutated genes in the bladder cancer was significantly associated with increased TMB and enhanced antitumor immune response, which might serve as a new biomarker to predict clinical prognosis and immune response in bladder cancer [42]. TMB might additionally confer immunotherapy sensitivity, so further study which genes contribute more to increase TMB would be helpful for targeted therapy combination. The colorectal cancers with PIK3CA mutation showed a higher TMB than nonmutated cancers [43]. In this study, some high correlated TMB genes, such as ATF4 in DLBC, EIF2AK3 in LAML, MAF in DLBC, MAFF in ACC, MAFK in THYM, MAPK8 in ACC, NRF2 in THYM, and RIT1 in ACC (correlation coefficient > 0.4), should be focused on. MSI generated from loss or gain of repetitive DNA tracts, which was used as diagnostic phenotype in endometrial, gastrointestinal, and colorectal tumors. Those cancers were divided into MSI, MSI-H (microsatellite instability-high), MSI-L (microsatellite instability-low), and MSS (microsatellite stability) clusters. Pembrolizumab and nivolumab were approved by the FDA for the treatment of MSI-H cancers. In the clinical trial of pembrolizumab for colorectal cancer patients, the immune-related objective response rate of MSI-H patients was 40%, while that of MSS patients was 0% [44]. Several lines of evidence showed that EP300 had mononucleotide repeats in exons 27 and 31, which might be mutation targets in gastric and colorectal cancers with microsatellite instability. The expressional loss of EP300 might be one of the meaningful characteristics for MSI-H cancers [45]. Another study identified the role of MSI status in mutations of exons 9 and 20 of the PIK3CA gene in gastric cancers. A majority of patients with the PIK3CA mutation had MSI and had poor 5-year survival rate. The mutations of exons 9 and 20 showed different 5-year survival rate and for patients with the mutation in exon 9 were poor [46]. In this study, some high correlated MSI genes, such as BACH1 in DLBC, CUL3 in DLBC, MAFK in DLBC, MAPK1 in DLBC, MAPK9 in ACC, PIK3CA in DLBC, and PRRT2 in CHOL (correlation coefficient > 0.4), should be focused on. CNV might drive cancer progression through diversified forms, including single deletion, single gain, double deletion, and double gain. The expression of corresponding genes might be affected by CNV status, and expression dysregulation occurred in various cancers. For example, PIK3CA CNV gain was noted in cervical cancer patients. Compared to CNV gain with wild type PIK3CA patients, CNV normal with positive PIK3CA mutation was associated with poorer OS and trend to worse PFS. This study indicated that PIK3CA mutational status with CNV status (delete, gain, or normal) might be important in predicting outcome in cervical cancer patients [47]. TMB, MSI, and CNV that were quantifiable clinical indexes, combined with several biomarkers, would be a new paradigm for clinical practice.

Accumulating evidence demonstrated some crosstalk mechanisms between oxidative stress and immune regulation, including immune evasion, inflammation, innate immune responses, adaptive immunity, immune-related gene expression, activation, proliferation and differentiation of immune-related cells, immune cell interaction, immune suppression, and inflammatory mediators [48, 49]. Under many pathophysiologic conditions, oxidative stress and inflammatory responses were integrated and amplified in specialized cell types, such as cancer cells, stromal cells, and essential immune cells, to facilitate the progression of disease [50]. For example, inflammasomes were cytoplasmic multiprotein complexes and played a crucial role in immune surveillance. They could be activated by ROS production to secret proinflammatory cytokine interleukin (IL)-1*β* and IL-18 [51]. The oxidative stress mediators also suppressed cellular immune system in tumor microenvironment, especially effector T cell function. The T cell state and differential response might depend on how long and how much ROS exposure. Some T cells were even dead when they were exposed to elevated ROS concentrations for a long time [52]. In turn, high active ROS production can also be generated by neutrophils and macrophages in the form of “oxidative burst.” Thus, it can be stated that tumor microenvironment is virtually a cauldron of redox and inflammatory substances [53]. As the oxidative stress played a critical role in the immune microenvironment and antitumor immune response, some studies evaluated PD-L1 expressions and functions treating with molecules and drugs for oxidative stress. Increasing ROS could promote PD-L1 expressions in cancer cells, and conversely decreasing ROS generally suppressed PD-L1. The variable PD-L1 response to ROS modulation showed complex interplay between oxidative stress and immune microenvironment [54]. Our data showed that the expressions of all selected NRF2 pathway-related genes were significantly different among six immune clusters based on TCGA Pan-Cancer data, and those immune system influenced wound healing, IFN-gamma dominant, lymphocyte depleted, inflammatory, immunologically quiet, and TGF-beta dominant. Further analysis of the association of immune score and immune-related cells revealed the similar results; namely, those NRF2 pathway-related genes played roles in immune regulation. Most of the findings in this study were consistent with previous reports according to GenCLiP 3 dataset (http://ci.smu.edu.cn/genclip3/GeneAssociation.php) [55], and many NRF2 pathway-related genes participated in multiple immune responses, including MAPK1, MAPK8, JUN, PIK3CA, MAPK3, FOS, MAPK9, EP300, KEAP1, CREBBP, MAF, ATF4, GSK3B, EIF2AK3, FOSL1, CUL3, MAPK7, BACH1, and NRF2. Many evidences demonstrated the involvement of NRF2 in immune evasion; for example, melanocytes triggered the expression of PD-L1 with exposure of UV, which mediated inhibitory interactions between effector T cells and cancer cells in an NRF2-dependent manner [56]. BACH1/2 participated in oxidative stress-mediated apoptosis and was involved in macrophage-mediated innate immunity, B cell differentiation, adaptive immune response, and T cell homeostasis [57]. Inhibiting the function of PD-1/PD-L1 became one of the effective methods to activate human immune system and significantly improve the prognosis of various cancers. The reduced MAPK1 phosphorylation was proved to inhibit PD-L1 expression and improve the immune response to pancreatic cancer [58]. Jun/FOS was a central driver of toll-like receptor 7-induced immune responses by dendritic cells, which could regulate CCL2 production and IL-23 expression to recruit plasmacytoid dendritic cells [59]. PI3K-AKT signaling played a crucial role in immune cell development, especially in CD4+ T cell differentiation. When the pathway was blocked, the expressions of key regulators of T follicular helper cells were remarkably downregulated, including LCOS, TCF7, BCL6, and CXCR5 [60]. The patterns of tumor-infiltrating immune cells were related to tumor mutations and clinicopathological parameters. One study analyzed somatic mutations and tumor mutational burden and constructed immune patterns in 197 patients with non-small-cell lung cancer. KEAP1 was one of the identified somatic mutations that correlated with specific immune cell infiltrates. The evaluation of somatic mutation states and immune patterns helps to better define the immunogenic potency in immunotherapy [61]. The transcription factor c-Maf controlled immune responses by inducing anti-inflammatory cytokine IL-10 in CD4+ T cells, including helper T cells, TH2 cells, and TH17 cells [62]. This study supported an immune-activated/inhibited role of NRF2 pathway-related genes in various cancers. A regulation of the immune effects of the NRF2 pathway-related genes might therefore have the potential to increase the responsiveness to existing checkpoint inhibitor immune therapy or trigger the endogenous antitumor response for cancer therapy.

Cancer stem cells (CSCs) have the properties of unlimited growth, resistance to existing chemotherapy, and generation of diverse cancer cells. The majority of cancer cells might be destroyed with current therapies, but many researchers believed that CSCs without eradicating could lead to tumor recurrence and metastasis [63]. Studies on CSCs have shown that different oxidative stress-related signaling pathways were involved in stemness processes. ROS maintained the stemness-associated properties of cancer cells and promoted phenotypic plasticity. When compared to non-CSCs, CSC subpopulation was tested low ROS level in the tumor mass. Lower level of intracellular ROS helped acquisition of stemness during neoplasia [64]. Immunohistochemical analyses showed colocalization of stemness markers and oxidative DNA lesions in cancer cells [65]. This study also explored the association between expressions of NRF2 pathway-related genes and RNAss. The majority of the genes showed negative correlation with RNAss, and some of them were reported in previous stem cell studies according to GenCLiP 3 dataset (http://ci.smu.edu.cn/genclip3/GeneAssociation.php) [55], including MAPK1, MAPK3, MAPK8, JUN, EP300, ATF4, KEAP1, FOSL1, FOS, MAPK9, PRRT2, BACH1, GSK3B, RIT1, EIF2AK3, and MAPK7. The study demonstrated that the stem cell microenvironment gained a powerful proliferative ability and stem phenotype through activation of PI3K signaling pathway. In comparison to retinal pigment epithelium cells cultured alone, the cells cocultured with stem cells had a higher colony-forming efficiency with significantly upregulated PI3K pathway-related genes [66]. The inhibition of activated MAPK1 could enhance stemness maintenance in the tumor microenvironment, which suggested that phosphorylated MAPK could be a putative target for cancer treatment. The development of inhibitors in MAPK signaling pathway might change the current stemness-based effects [67], such as chemoresistance. MYH9 was an effective promoter of tumor stemness, which degraded GSK3B and *β*-catenin destruction complex by ubiquitin-mediated process to induce the downstream tumor stemness phenotype. C-Jun transcriptionally stimulated MYH9 expression to form MYH9/GSK3B/*β*-catenin/JUN feedback loop regulating CSC properties in hepatocellular carcinoma [68]. Additionally, this study also explored the association of the NRF2 pathway-related genes with drug sensibility, and the significant results would provide potential new therapeutic strategy. Finally, the different prognosis models in LUSC, BRCA, and STAD might reveal that the same functional genes were weighted unequally in heterogeneous tumors. Those prognosis models made one start to rethink the emphasis that placed molecular biomarker pattern and therapeutic targets in the context of predictive, preventive, and personalized medicine (PPPM) [69]. The individualized patient stratification and predictive/prognostic assessment and therapeutic targets/drugs for personalized therapy of different kinds of cancer patients would be a better paradigm due to cancer heterogeneity.

## 5. Conclusions

Pan-cancer system analysis revealed that the NRF2 pathway-related genes altered in different levels in various cancers. The comprehensive understanding of these genes would provide full view on expression, mutation, CNV, MSI, clinical relevance, crosstalk with other system, stemness, and drug sensitivity. The pan-cancer analysis was necessary and effective method to find the concentrated performance, meanwhile single kind of cancer could not be ignored, due to the guideline of PPPM in individualized medicine.

## Figures and Tables

**Table 1:** The correlation coefficients of the Cox regression in LUSC, BRCA, and STAD. ^∗^*p* < 0.05 and ^∗∗^*p* < 0.01.

Cancer type	Id	Coef	HR	HR.95 L	HR.95H	*p* value
LUSC	CUL3	0.64	1.90	1.04	3.46	0.036^∗^
	EIF2AK3	-0.37	0.69	0.44	0.97	0.049^∗^
	JUN	0.29	1.34	1.06	1.70	0.015^∗^
	MAFF	0.35	1.42	1.05	1.92	0.021^∗^
	MAPK3	0.40	1.49	1.04	2.31	0.049^∗^

BRCA	CREBBP	-0.54	0.58	0.35	0.96	0.035^∗^
	EP300	-0.55	0.58	0.34	0.99	0.047^∗^
	FOS	-0.19	0.83	0.71	0.97	0.016^∗^
	GSK3B	0.51	1.66	1.04	2.99	0.043^∗^
	MAFF	-0.30	0.74	0.30	0.99	0.042^∗^
	MAFK	0.55	1.73	1.16	2.60	0.008^∗∗^
	PIK3CA	0.60	1.83	1.03	3.23	0.038^∗^

STAD	BACH1	-0.58	0.56	0.32	0.98	0.042^∗^
	KEAP1	-0.59	0.55	0.32	0.96	0.036^∗^
	MAFG	-0.98	0.37	0.21	0.64	0.001^∗∗^
	MAPK3	0.36	1.43	0.11	2.15	0.044^∗^
	PIK3CA	0.99	2.74	1.33	5.64	0.006^∗∗^

## Data Availability

All the data used in this study were collected in this article and supplemental materials.

## Abbreviations

ACC: Adrenocortical carcinoma
ARE: Antioxidant response element
BLCA: Bladder urothelial carcinoma
BRCA: Breast cancer
CESC: Cervical squamous cell carcinoma and endocervical adenocarcinoma
CHOL: Cholangiocarcinoma
CNV: Copy number variations
COAD: Colon adenocarcinoma
CSCs: Cancer stem cells
DEGs: Differentially expressed genes
DLBC: Lymphoid neoplasm diffuse large B cell lymphoma
ECM: Extracellular matrix
ESCA: Esophageal carcinoma
GBM: Glioblastoma multiforme
HNSC: Head and neck squamous carcinoma
IPA: Ingenuity Pathway Analysis
Keap1: Kelch-like ECH-associated protein 1
KICH: Kidney chromophobe
KIRC: Kidney renal clear cell carcinoma
KIRP: Kidney renal papillary cell carcinoma
LAML: Acute myeloid leukemia
Lasso: Least absolute shrinkage and selection operator
LGG: Brain lower grade glioma
LIHC: Liver hepatocellular carcinoma
LUAD: Lung adenocarcinoma
LUSC: Lung squamous cell carcinoma
MDSCs: Myeloid-derived suppressor cells
MESO: Mesothelioma
MIS: Microsatellite instability
MSI-H: Microsatellite instability-high
MSI-L: Microsatellite instability-low
MSS: Microsatellite stability
Nrf2: Nuclear factor E2-related factor 2
OV: Ovarian serous cystadenocarcinoma
PAAD: Pancreatic adenocarcinoma
PCPG: Pheochromocytoma and paraganglioma
PPPM: Predictive, preventive, and personalized medicine
PRAD: Prostate adenocarcinoma
READ: Rectum adenocarcinoma
RNAss: RNA expression-based cancer stemness index
ROS: Reactive oxygen species
SARC: Sarcoma
SKCM: Skin cutaneous melanoma
STAD: Stomach adenocarcinoma
TCGA: The Cancer Genome Atlas
TGCT: Testicular germ cell tumors
THCA: Thyroid carcinoma
THYM: Thymoma
TMB: Tumor mutation burden
Tregs: Regulatory T cells
UCEC: Uterine corpus endometrial carcinoma
UCS: Uterine carcinosarcoma
UVM: Uveal melanoma

## Supplementary Materials

Supplementary 1 Supplementary Figure 1: the differentially expressed NRF2 pathway-related genes between normal and tumor tissues across 33 cancer types.

Supplementary 2 Supplementary Figure 2: the correlation between Nrf2 pathway-related genes and immunity across 33 cancer types.

Supplementary 3 Supplementary Table 1: the expression of NRF2 pathway-related genes across 33 cancer types.

Supplementary 4 Supplementary Table 2: the variant class of Nrf2 pathway-related genes across 33 cancer types.

Supplementary 5 Supplementary Table 3: the TMB score of 33 cancer types.

Supplementary 6 Supplementary Table 4: the MSI score of 33 cancer types.

Supplementary 7 Supplementary Table 5: LogFC and p value of differentially expressed Nrf2 pathway-related genes across 33 cancer types.

Supplementary 8 Supplementary Table 6: the Cox regression for survival analysis of the Nrf2 pathway-related genes across 33 cancer types.

Supplementary 9 Supplementary Table 7: StromalScore, ImmuneScore, ESTIMATEScore, and TumorPurity across 33 cancer types.

Supplementary 10 Supplementary Table 8: the proportion of 22 immune cells across 33 cancer types.

Supplementary 11 Supplementary Table 9: the correlation between the Nrf2 pathway-related gene expressions and drug susceptibility.

Supplementary 12 Supplementary Table 10: the Cox regression was used to construct gene signature in training and test groups of cancers LUSC, BRCA, and STAD.

Supplementary 13 Supplementary Table 11: the clinical features and clusters based on riskscore of LUCS, BRCA, and STAD.
