# Supplementary material for: Machine Learning Identifies Pan-Cancer Landscape of Nrf2 Oxidative Stress Response Pathway-Related Genes
Source: Oxid Med Cell Longev. 2022 Feb 17;2022:8450087. doi: 10.1155/2022/8450087 (PMC8886747; doi:10.1155/2022/8450087)
Supplement: Supplementary 1 — Supplementary Figure 1: the differentially expressed NRF2 pathway-related genes between normal and tumor tissues across 33 cancer types. [file 8450087.f1.pdf]

Supplementary figure 1:
The differentially expressed NRF2 pathway-related genes between normal and tumor tissues across 33 cancer types.

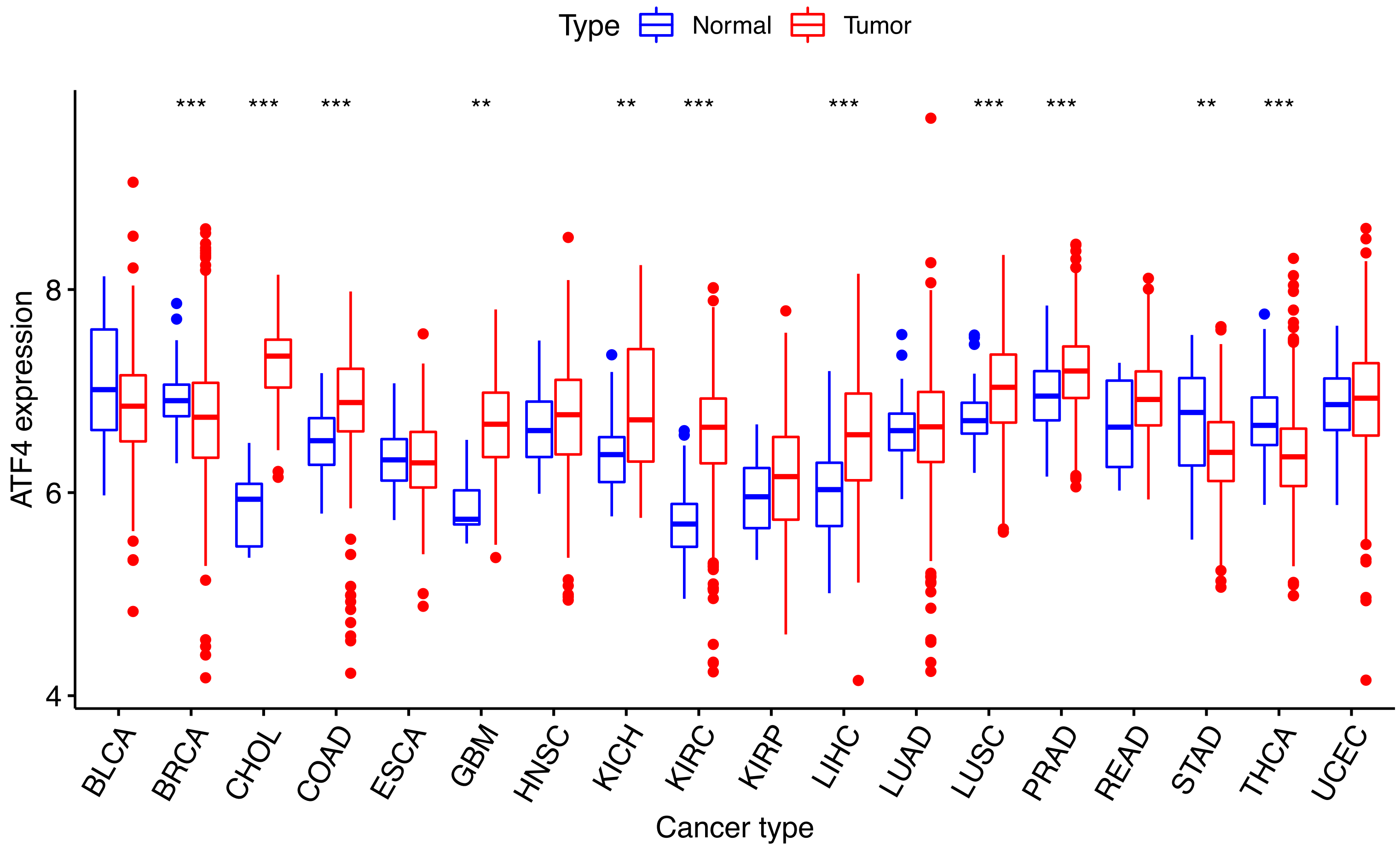

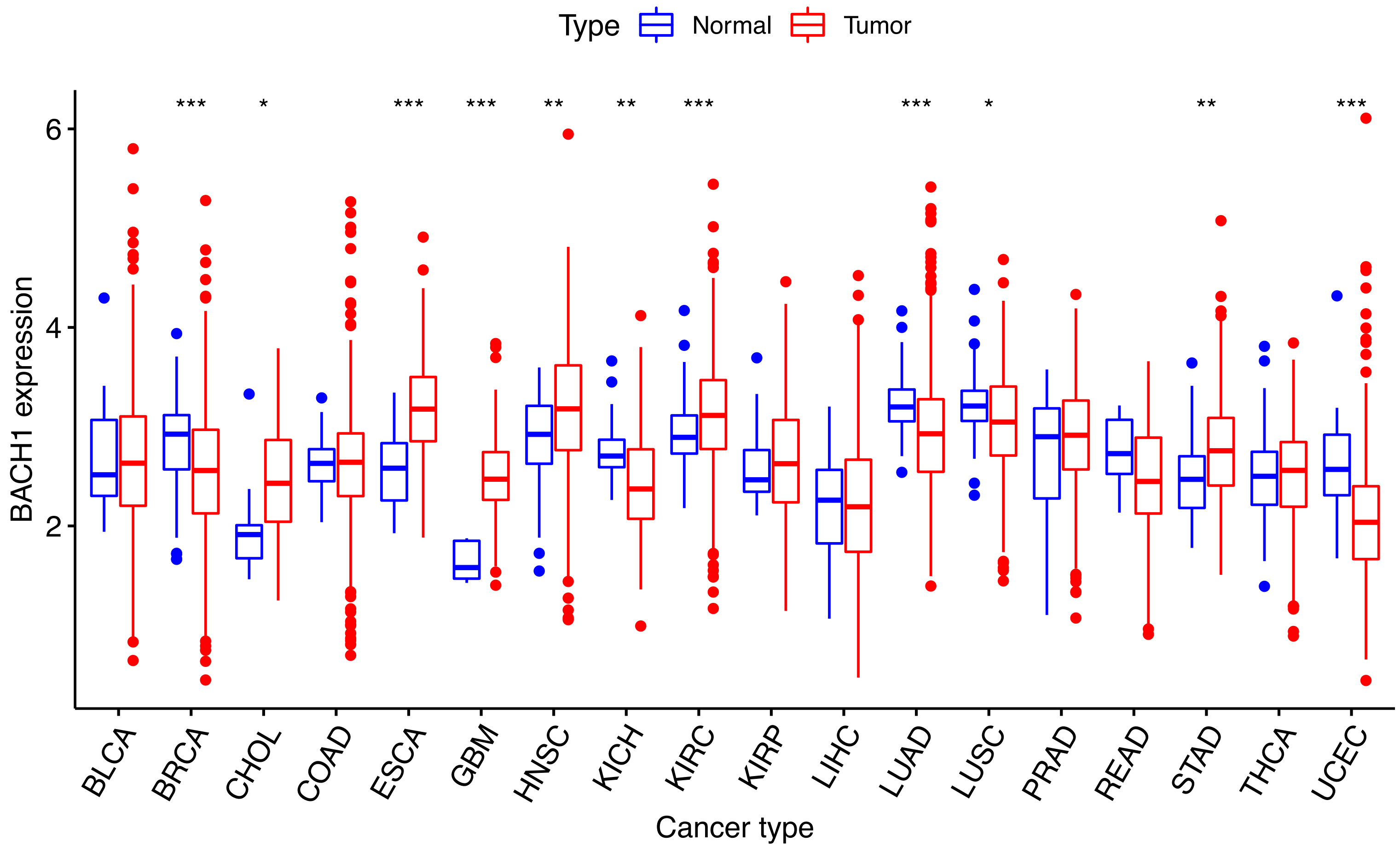

Type
Normal
Tumor
BACH1 expression
6
4
2
***
*
***
***
**
**
***
***
*
**
***
BLCA
BRCA
CHOL
COAD
ESCA
GBM
HNSC
KICH
KIRC
KIRP
LIHC
LUAD
LUSC
PRAD
READ
STAD
THCA
UCEC
Cancer type

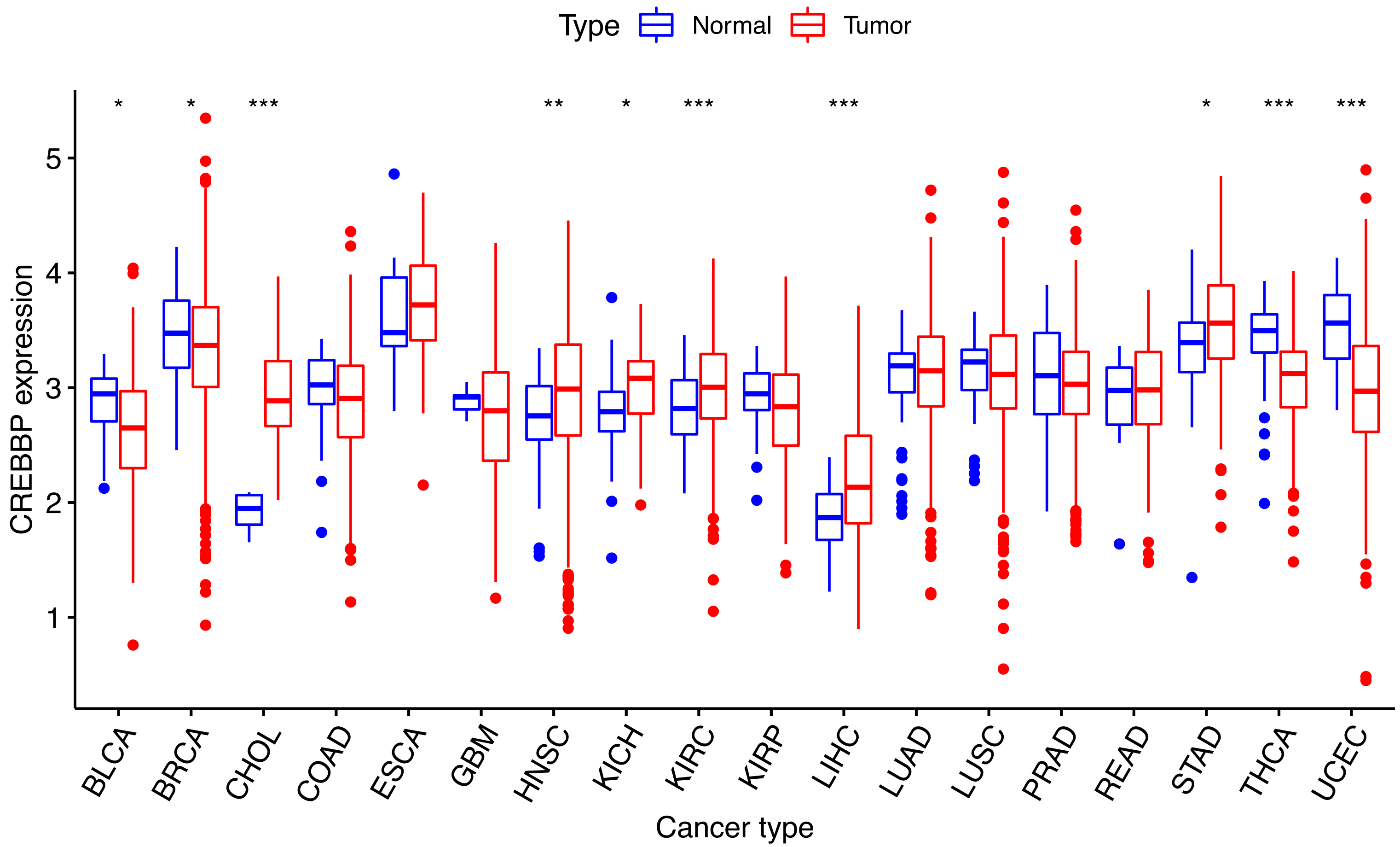
Type
Normal
Tumor
CREBBP expression
Cancer type
BLCA
BRCA
CHOL
COAD
ESCA
GBM
HNSC
KICH
KIRC
KIRP
LIHC
LUAD
LUSC
PRAD
READ
STAD
THCA
UCEC

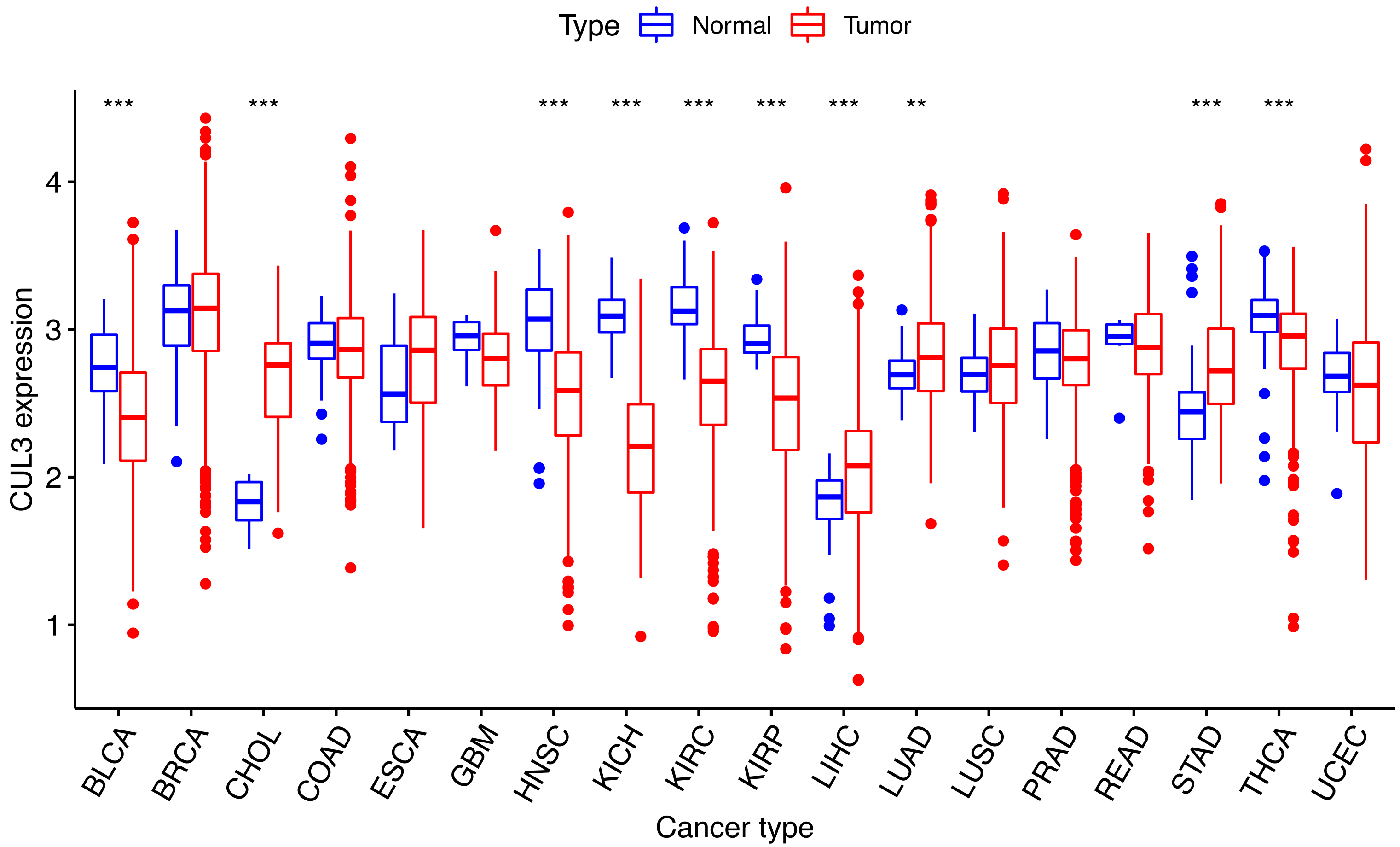
Type
Normal
Tumor
CUL3 expression
4
3
2
1
***
***
***
***
***
***
***
**
***
***
BLCA
BRCA
CHOL
COAD
ESCA
GBM
HNSC
KICH
KIRC
KIRP
LIHC
LUAD
LUSC
PRAD
READ
STAD
THCA
UCEC
Cancer type

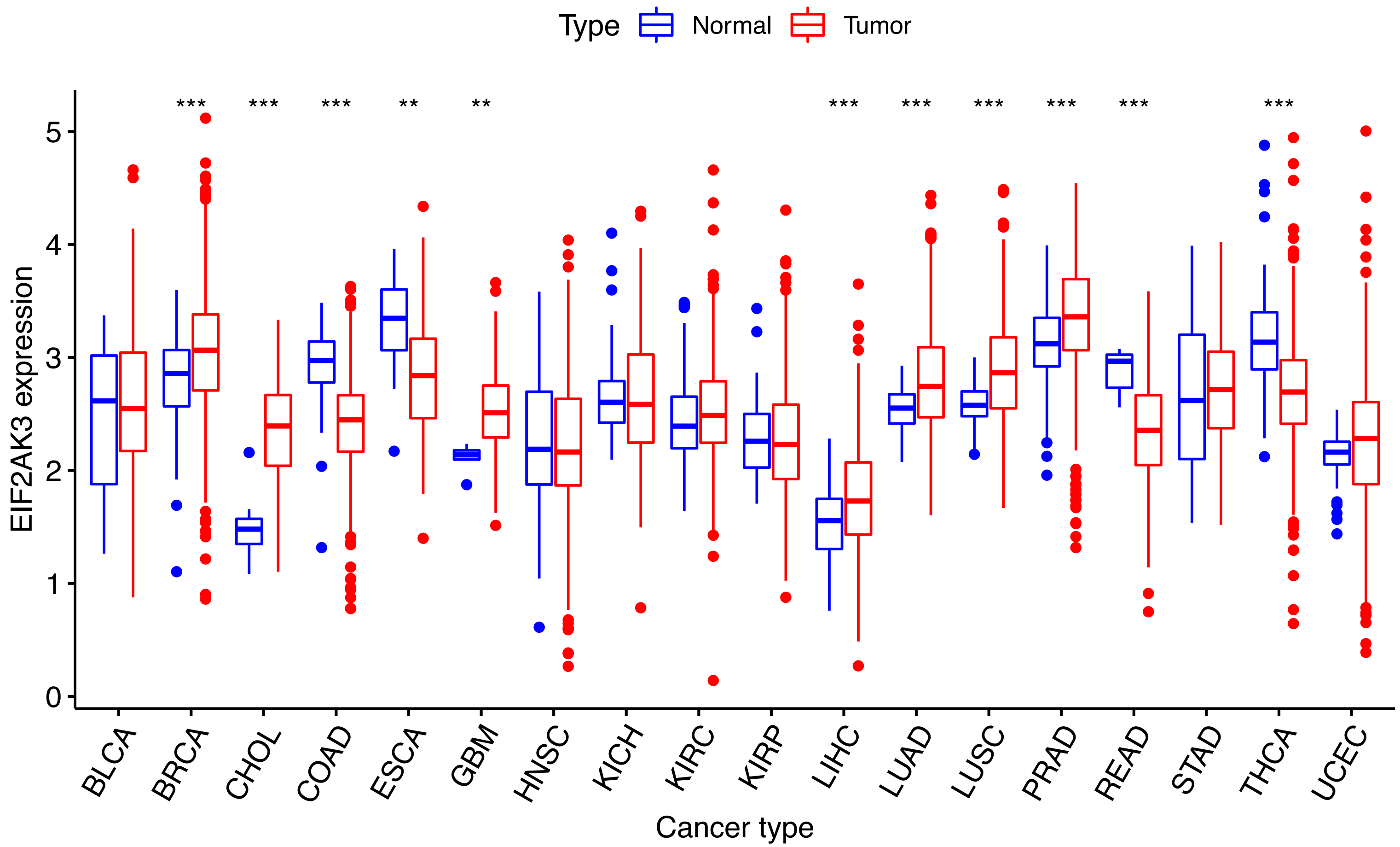
Type
Normal
Tumor
EIF2AK3 expression
5
4
3
2
1
0
***
***
***
**
**
***
***
***
***
***
***
BLCA
BRCA
CHOL
COAD
ESCA
GBM
HNSC
KICH
KIRC
KIRP
LIHC
LUAD
LUSC
PRAD
READ
STAD
THCA
UCEC
Cancer type

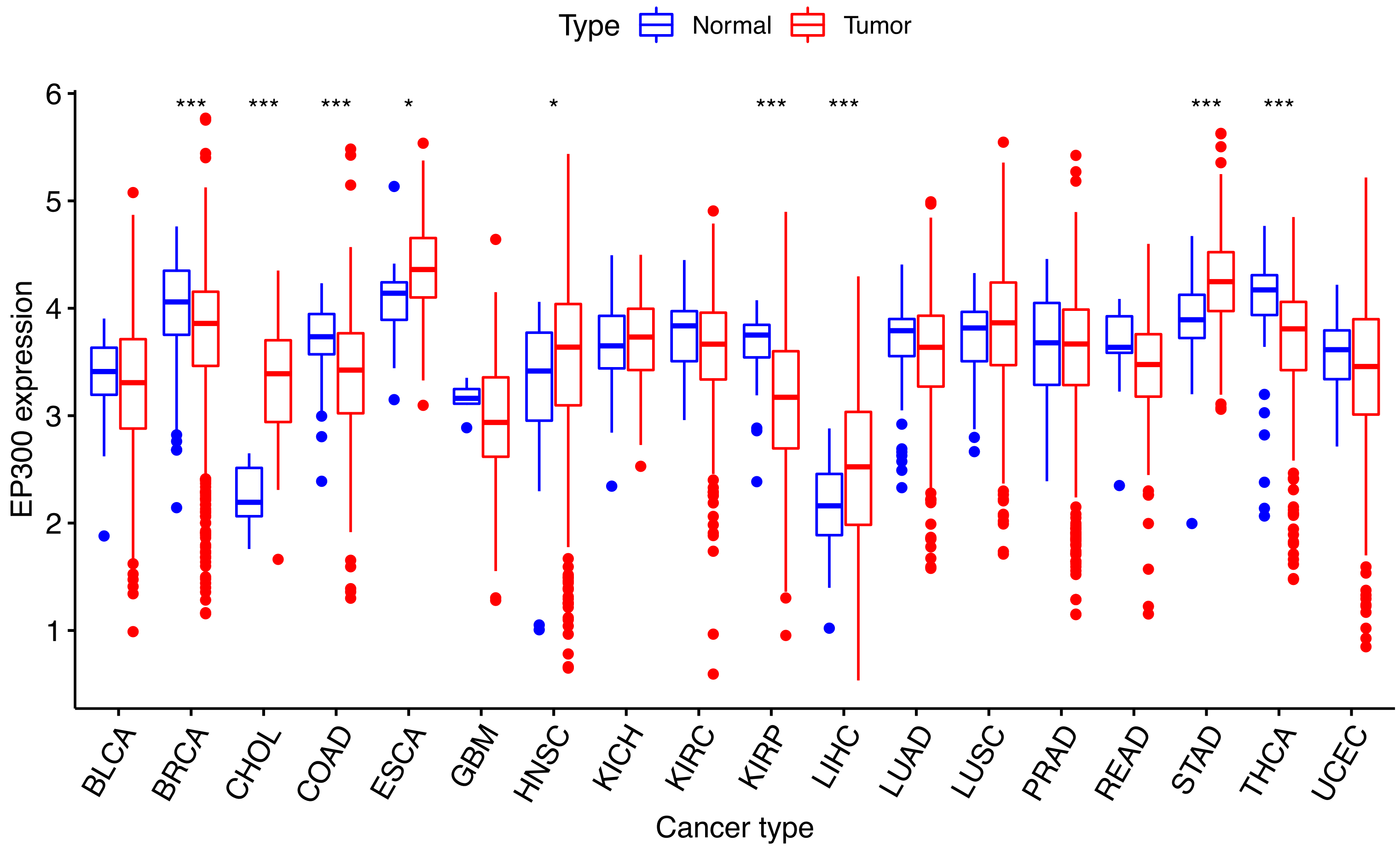

Type
Normal
Tumor
EP300 expression
6
5
4
3
2
1
***
***
***
*
*
***
***
***
***
BLCA
BRCA
CHOL
COAD
ESCA
GBM
HNSC
KICH
KIRC
KIRP
LIHC
LUAD
LUSC
PRAD
READ
STAD
THCA
UCEC
Cancer type

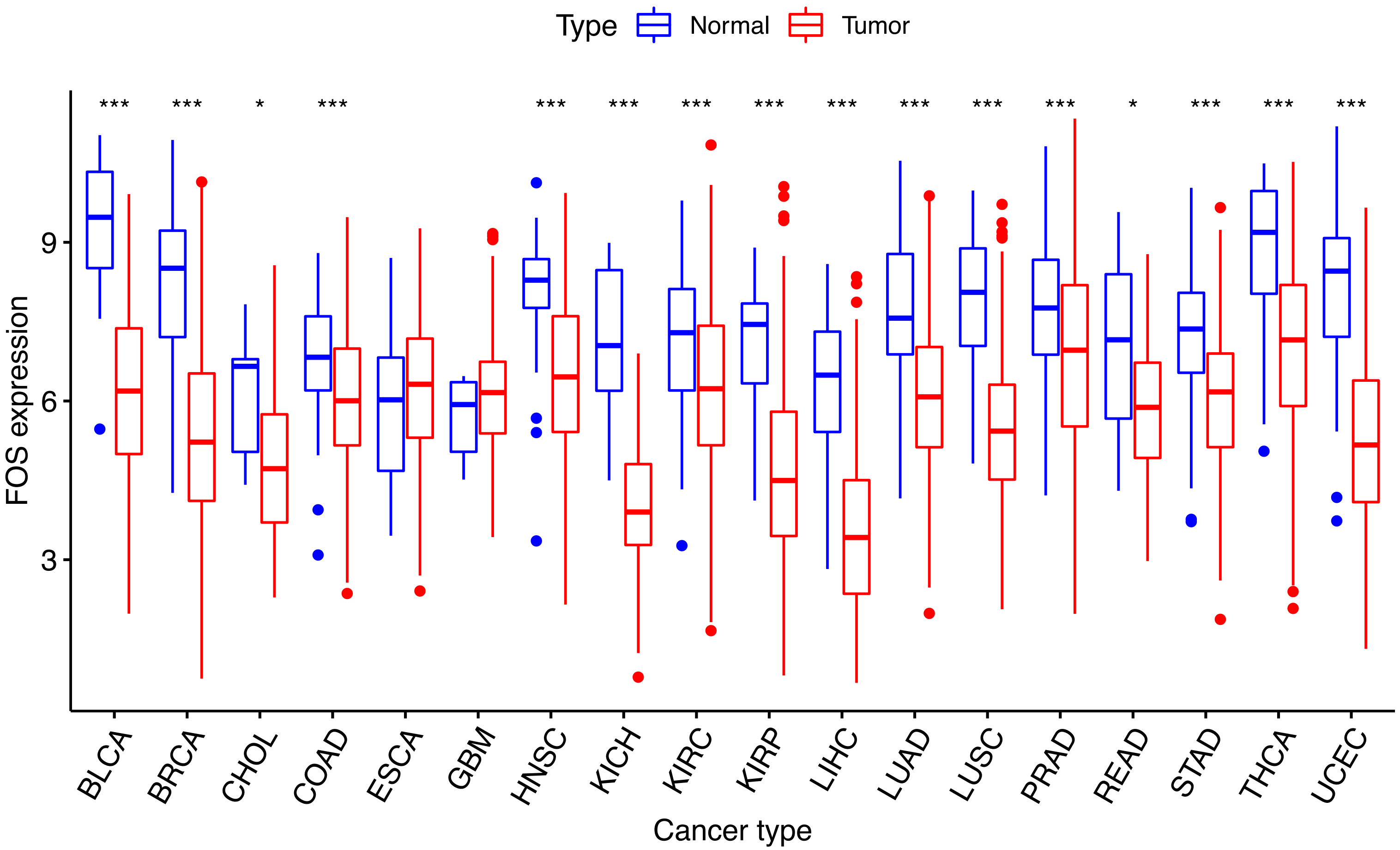

Type
Normal
Tumor
FOS expression
9
6
3
*** *** * *** *** *** *** *** *** *** *** *** * *** *** ***
BLCA
BRCA
CHOL
COAD
ESCA
GBM
HNSC
KICH
KIRC
KIRP
LIHC
LUAD
LUSC
PRAD
READ
STAD
THCA
UCEC
Cancer type

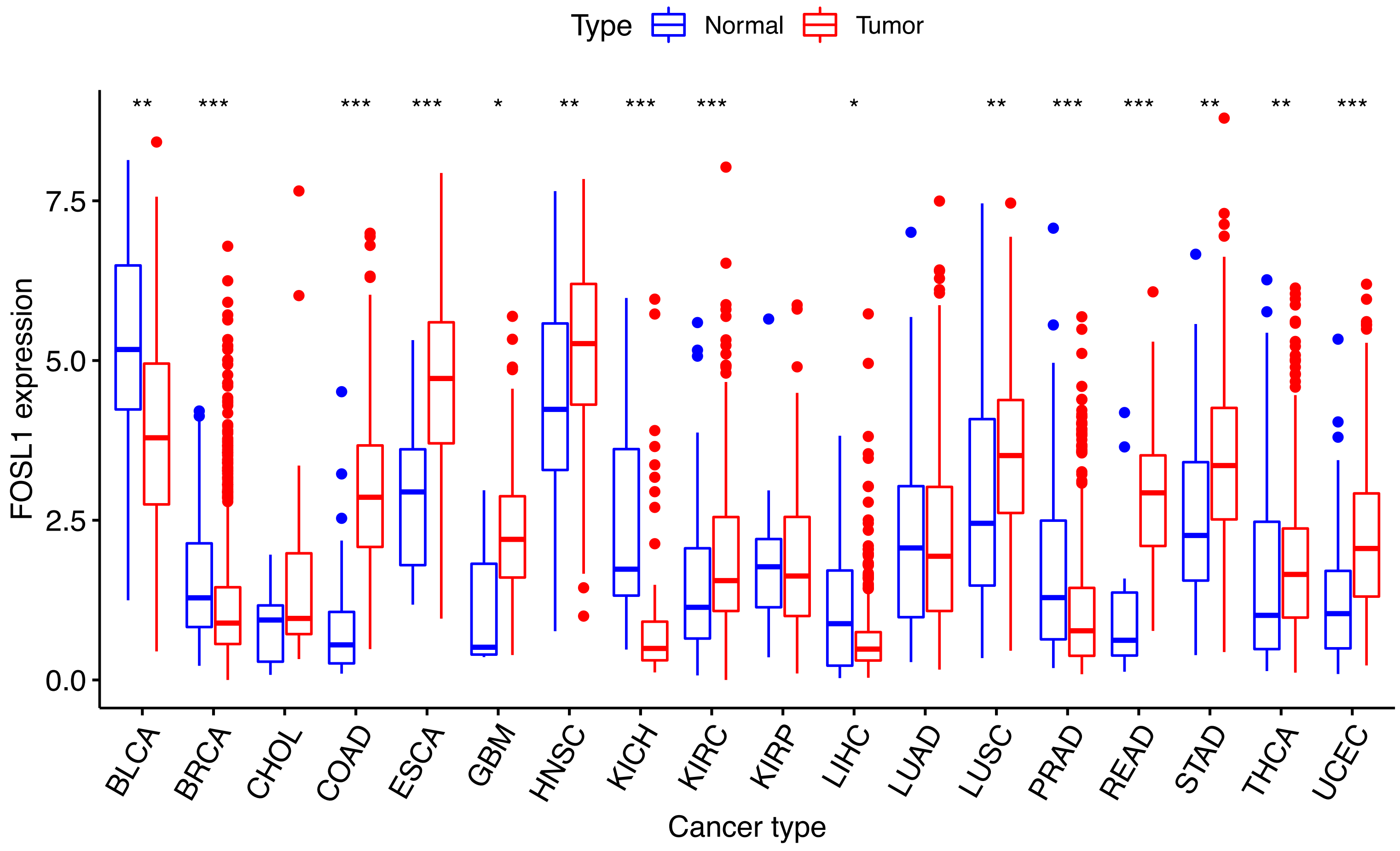

Type
Normal
Tumor
FOSL1 expression
0.0
2.5
5.0
7.5
**
***
***
***
*
**
***
***
*
**
***
***
**
**
***
BLCA
BRCA
CHOL
COAD
ESCA
GBM
HNSC
KICH
KIRC
KIRP
LIHC
LUAD
LUSC
PRAD
READ
STAD
THCA
UCEC
Cancer type

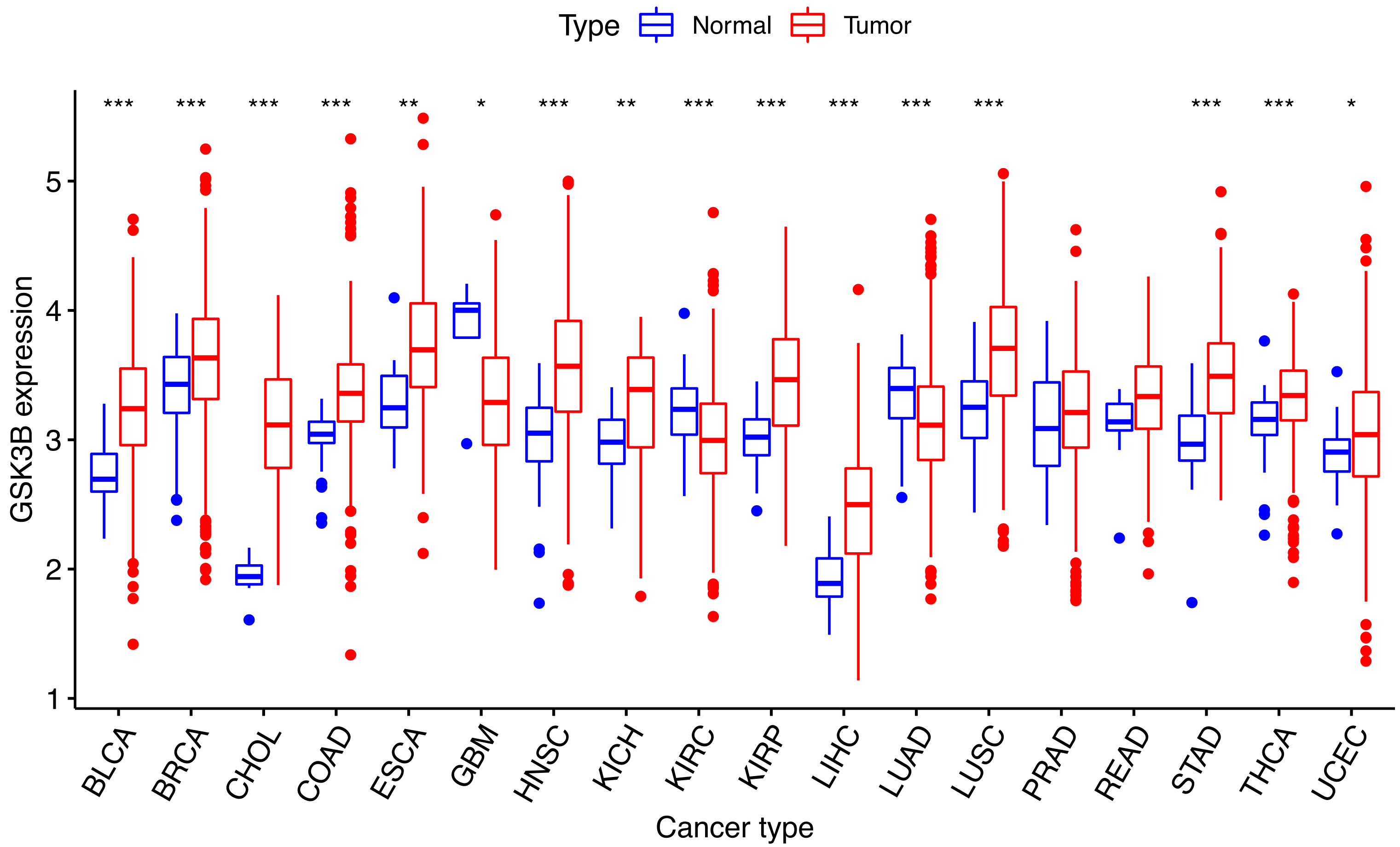
Type
Normal
Tumor
*** *** *** *** ** * *** ** *** *** *** *** *** *** *** *
GSK3B expression
5
4
3
2
1
BLCA
BRCA
CHOL
COAD
ESCA
GBM
HNSC
KICH
KIRC
KIRP
LIHC
LUAD
LUSC
PRAD
READ
STAD
THCA
UCEC
Cancer type

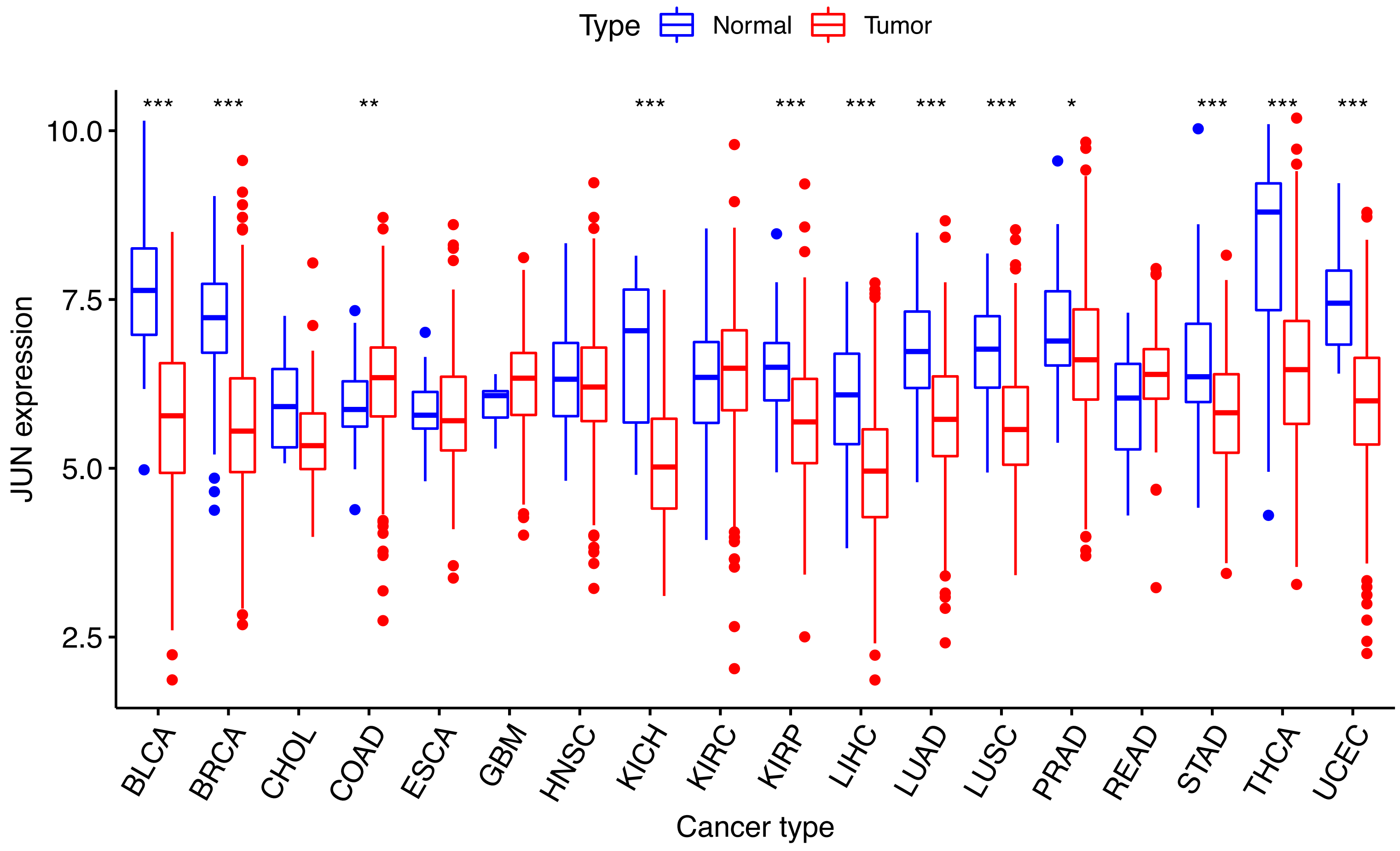

Type
Normal
Tumor
JUN expression
10.0
7.5
5.0
2.5
*** *** ** *** *** *** *** *** * *** *** ***
BLCA
BRCA
CHOL
COAD
ESCA
GBM
HNSC
KICH
KIRC
KIRP
LIHC
LUAD
LUSC
PRAD
READ
STAD
THCA
UCEC
Cancer type

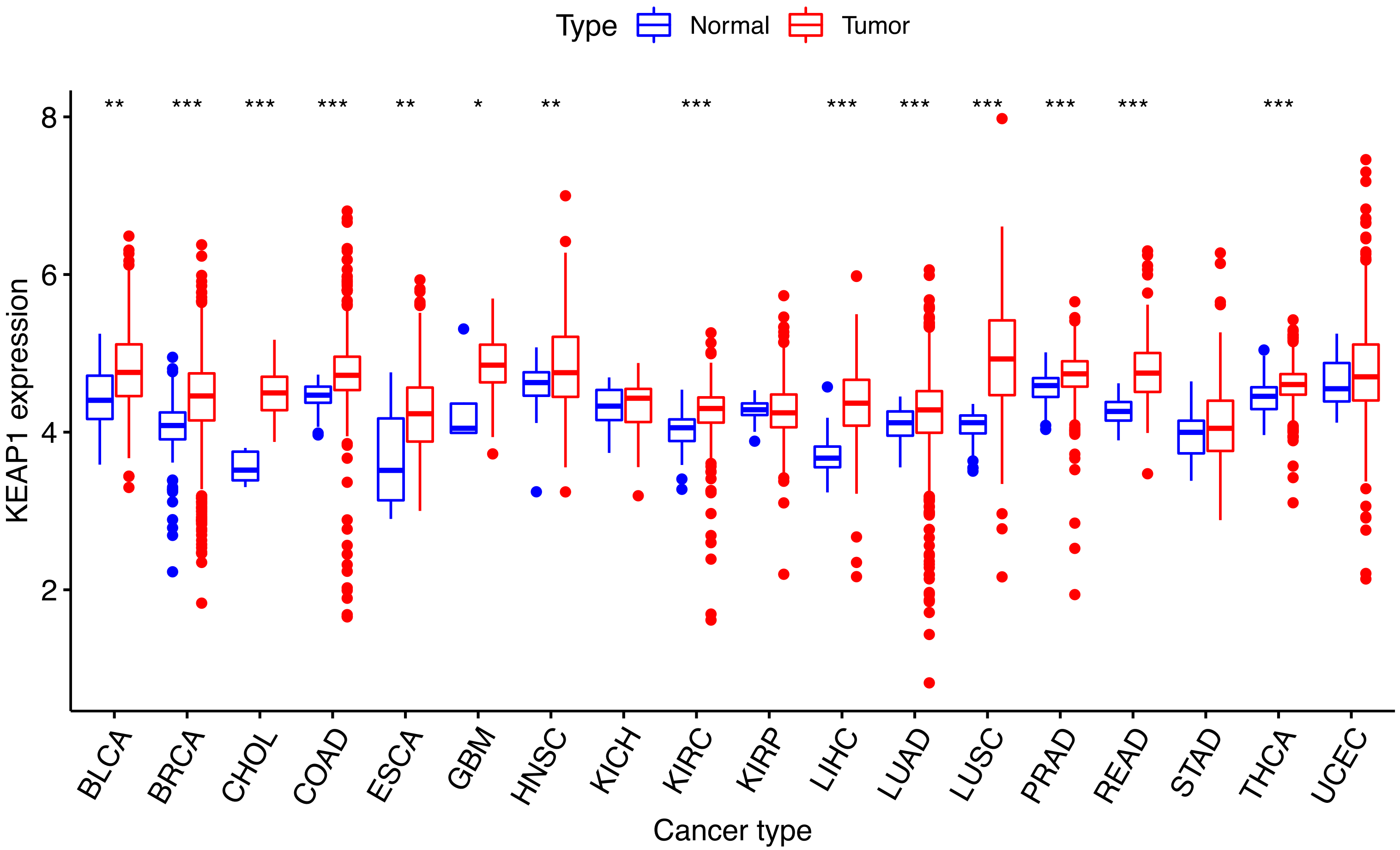
Type
Normal
Tumor
KEAP1 expression
Cancer type
BLCA
BRCA
CHOL
COAD
ESCA
GBM
HNSC
KICH
KIRC
KIRP
LIHC
LUAD
LUSC
PRAD
READ
STAD
THCA
UCEC
**
***
***
***
**
*
**
***
***
***
***
***
***
***

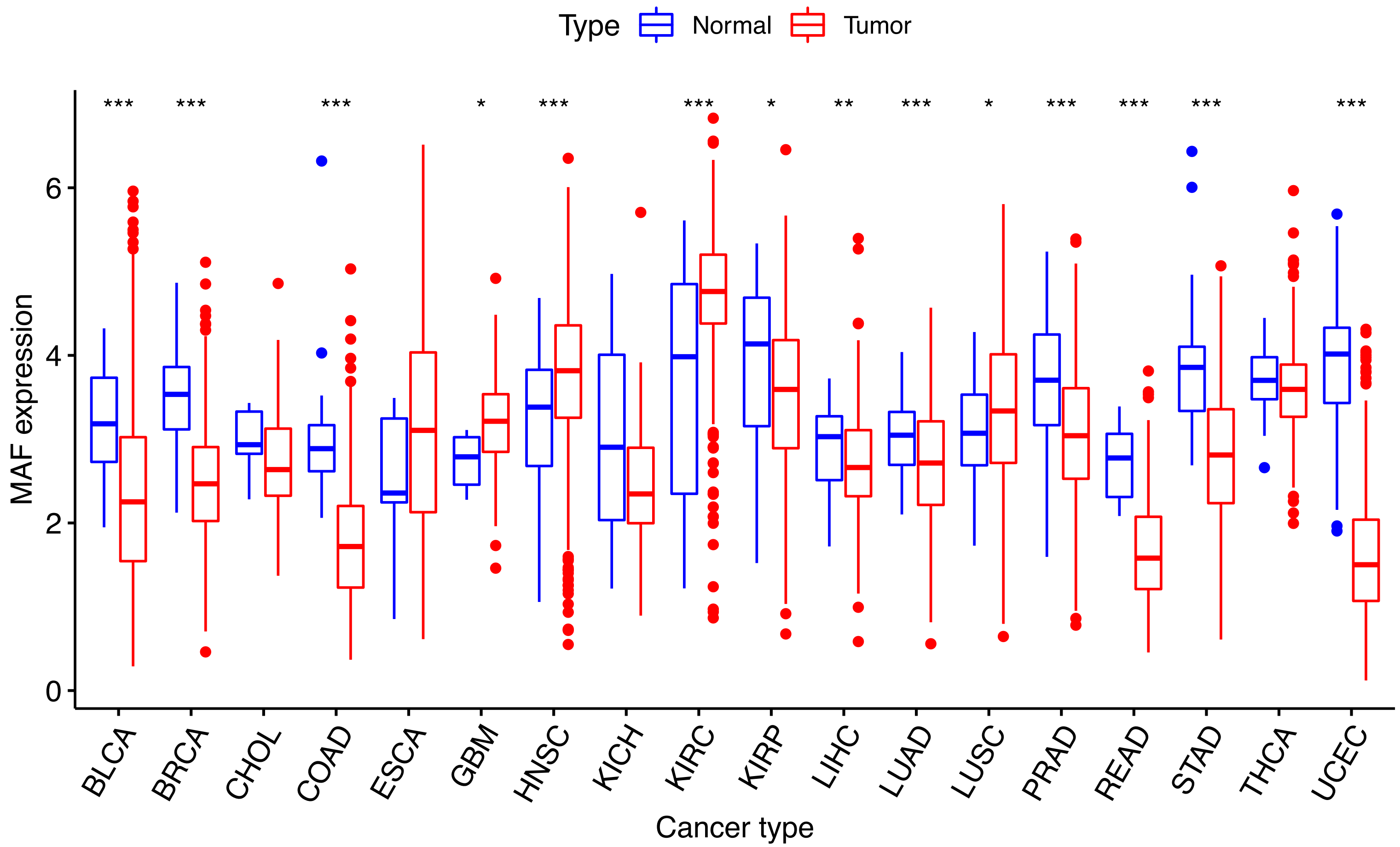

Type
Normal
Tumor
MAF expression
Cancer type
0
2
4
6
BLCA
BRCA
CHOL
COAD
ESCA
GBM
HNSC
KICH
KIRC
KIRP
LIHC
LUAD
LUSC
PRAD
READ
STAD
THCA
UCEC
***
***
***
*
***
***
*
**
***
*
***
***
***
***

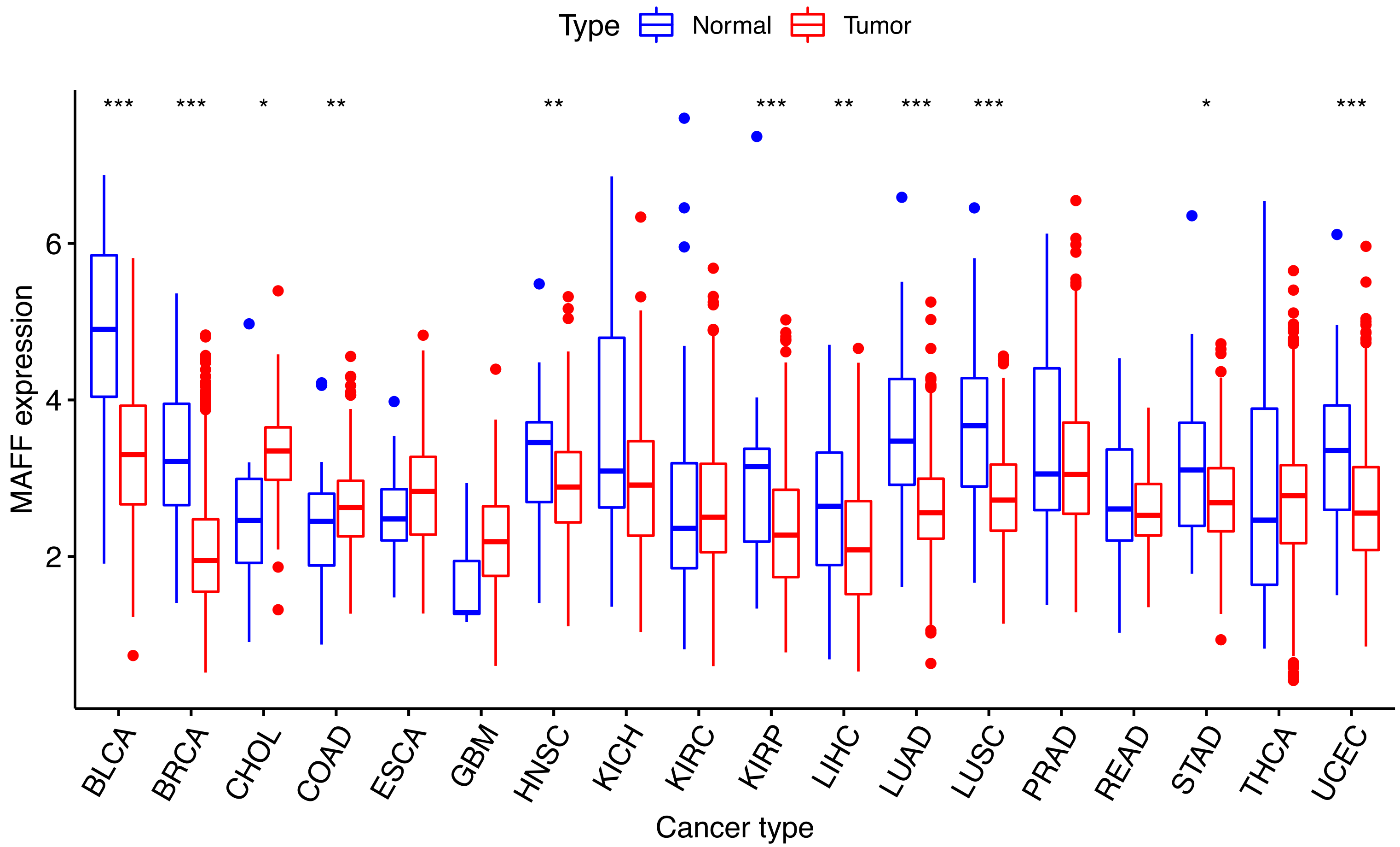

Type
Normal
Tumor
MAFF expression
6
4
2
*** *** * ** ** *** ** *** *** * ***
BLCA
BRCA
CHOL
COAD
ESCA
GBM
HNSC
KICH
KIRC
KIRP
LIHC
LUAD
LUSC
PRAD
READ
STAD
THCA
UCEC
Cancer type

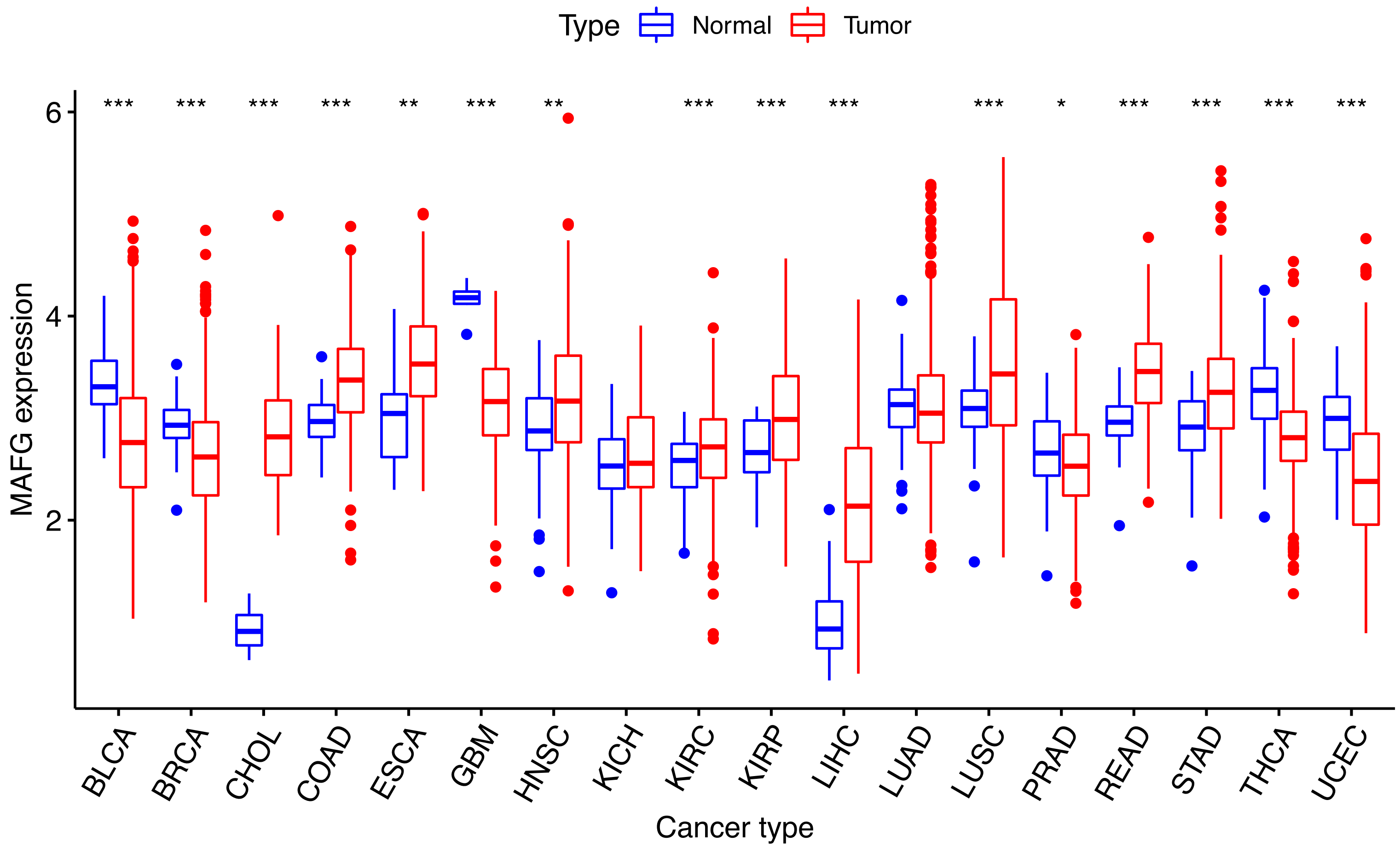

Type
Normal
Tumor
MAFG expression
6
4
2
*** *** *** *** ** *** ** *** *** *** *** * *** *** *** ***
BLCA
BRCA
CHOL
COAD
ESCA
GBM
HNSC
KICH
KIRC
KIRP
LIHC
LUAD
LUSC
PRAD
READ
STAD
THCA
UCEC
Cancer type

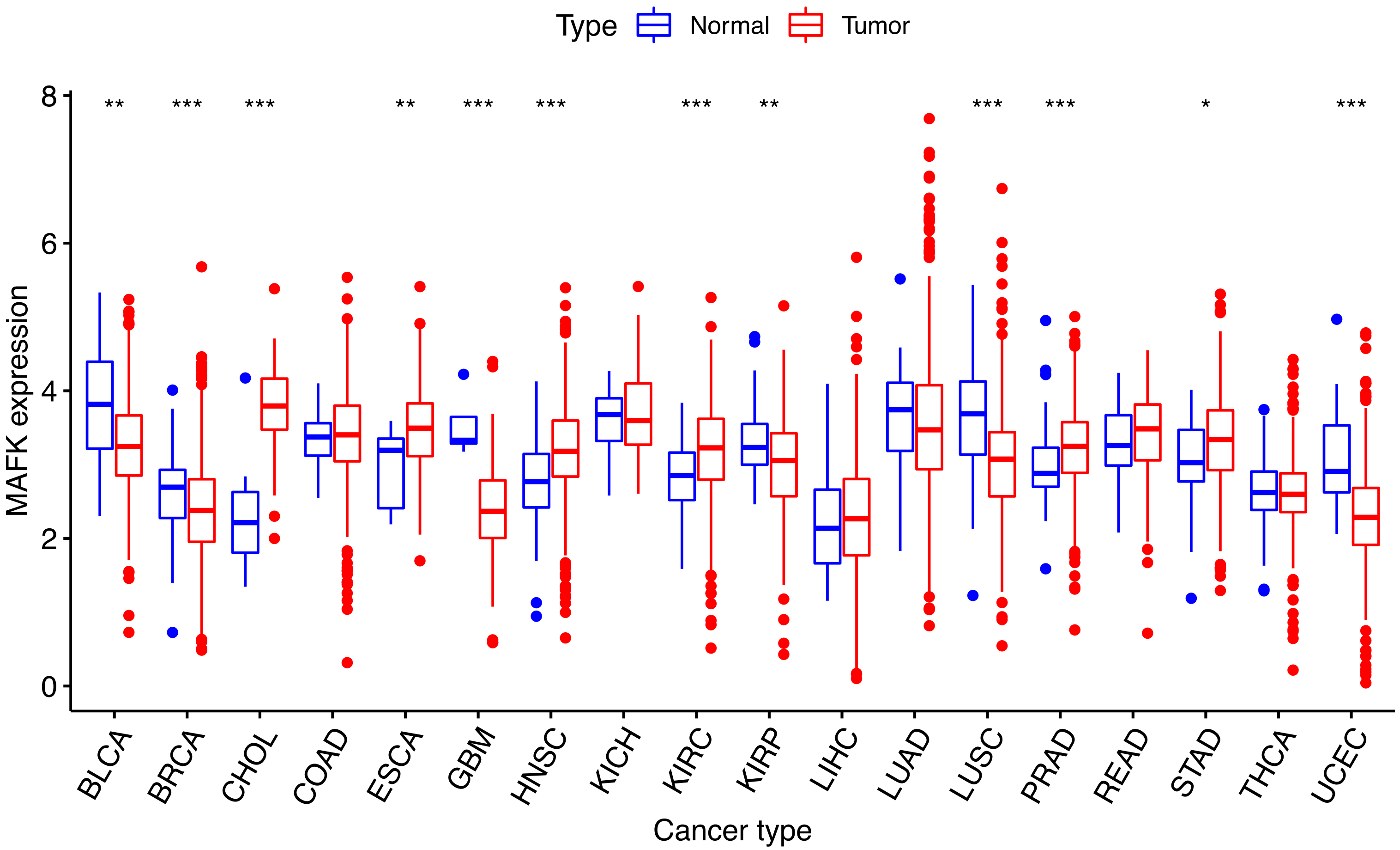
Type
Normal
Tumor
MAFK expression
8
6
4
2
0
**
***
***
**
***
***
***
**
***
***
*
***
BLCA
BRCA
CHOL
COAD
ESCA
GBM
HNSC
KICH
KIRC
KIRP
LIHC
LUAD
LUSC
PRAD
READ
STAD
THCA
UCEC
Cancer type

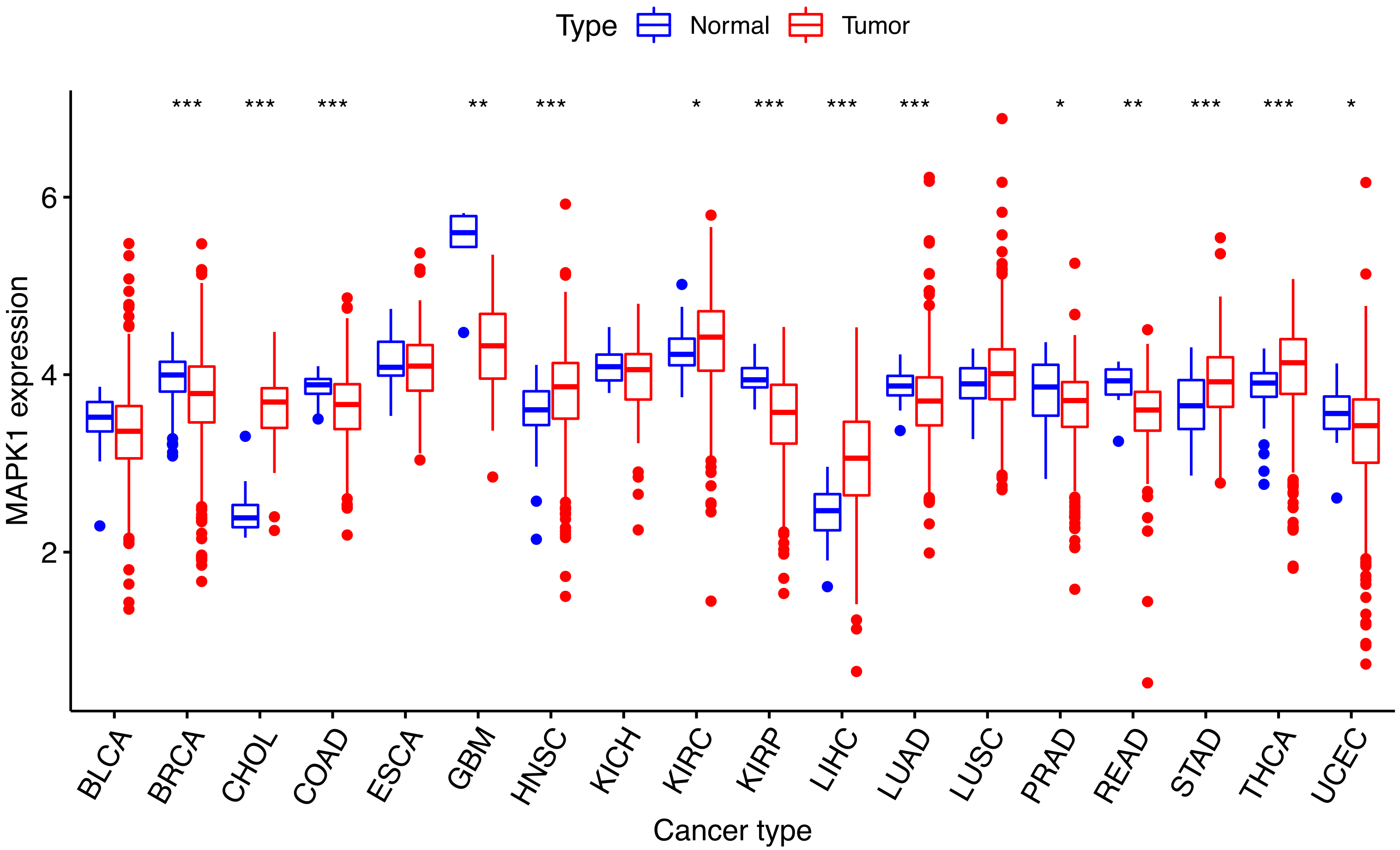
Type
Normal
Tumor
MAPK1 expression
6
4
2
*** *** *** ** *** * *** *** *** * ** *** *** *
BLCA
BRCA
CHOL
COAD
ESCA
GBM
HNSC
KICH
KIRC
KIRP
LIHC
LUAD
LUSC
PRAD
READ
STAD
THCA
UCEC
Cancer type

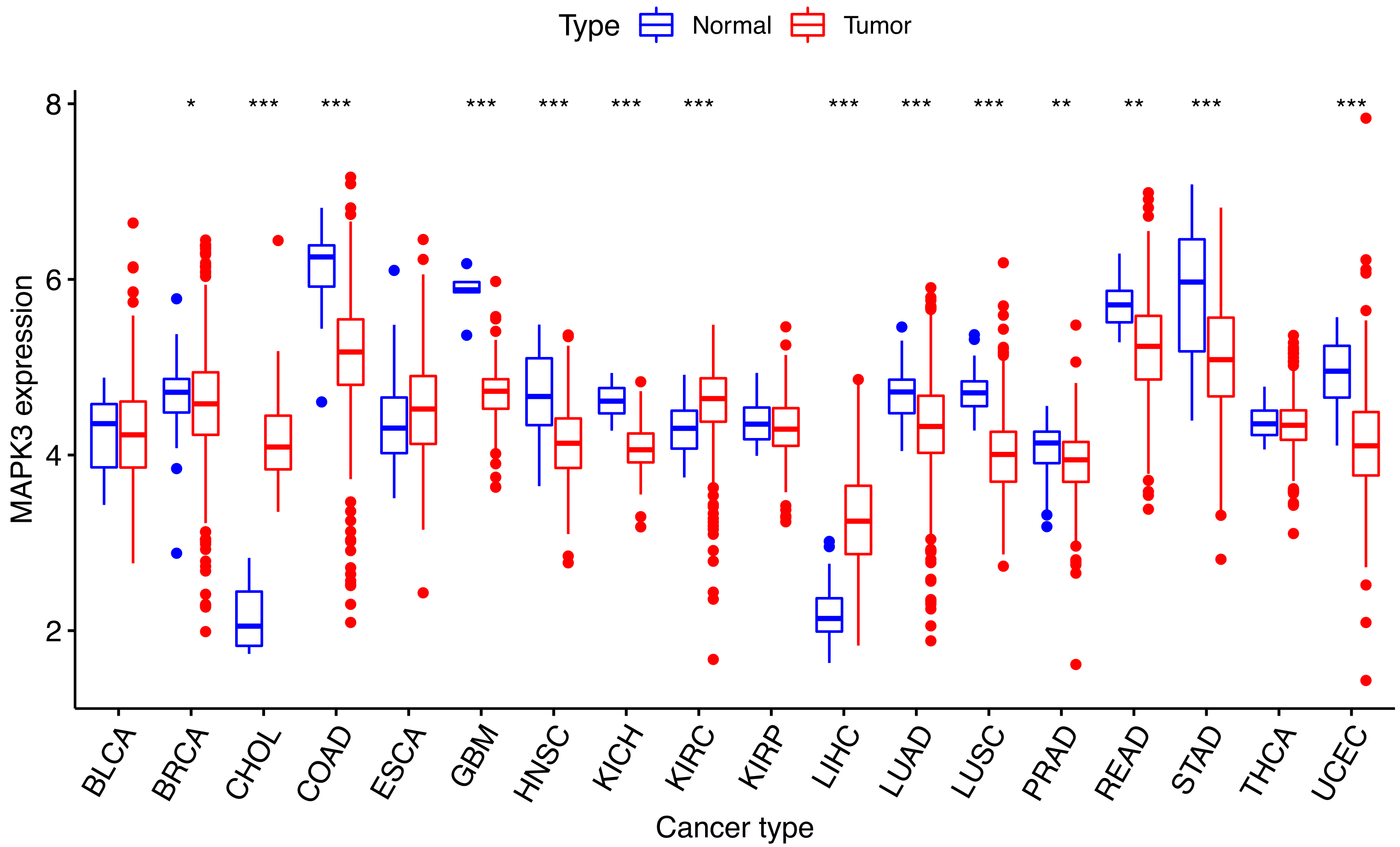
Type
Normal
Tumor
MAPK3 expression
8
6
4
2
*
***
***
***
***
***
***
***
***
***
**
**
***
***
BLCA
BRCA
CHOL
COAD
ESCA
GBM
HNSC
KICH
KIRC
KIRP
LIHC
LUAD
LUSC
PRAD
READ
STAD
THCA
UCEC
Cancer type

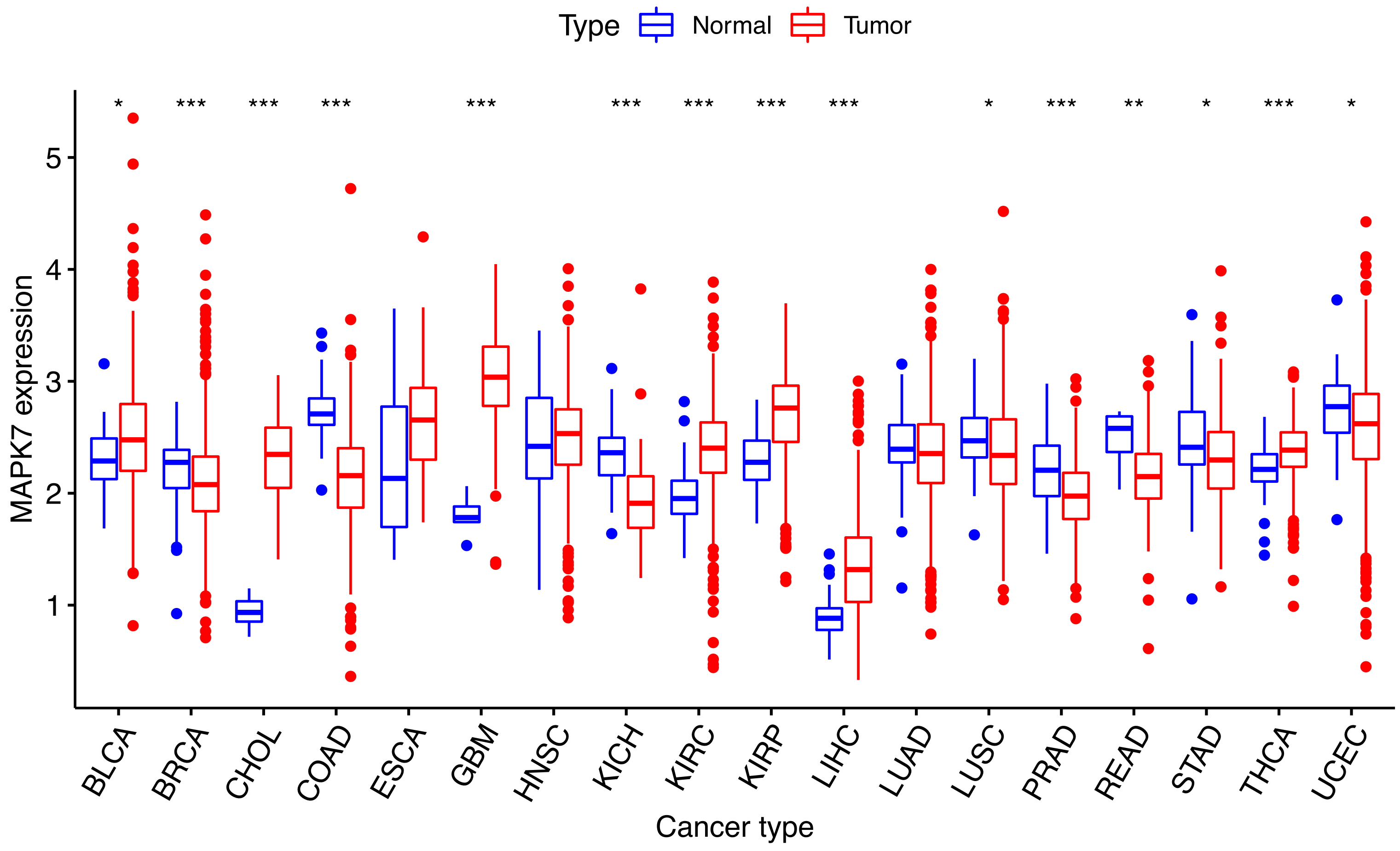

Type
Normal
Tumor
MAPK7 expression
5
4
3
2
1
*
***
***
***
***
***
***
***
***
*
***
**
*
***
*
BLCA
BRCA
CHOL
COAD
ESCA
GBM
HNSC
KICH
KIRC
KIRP
LIHC
LUAD
LUSC
PRAD
READ
STAD
THCA
UCEC
Cancer type

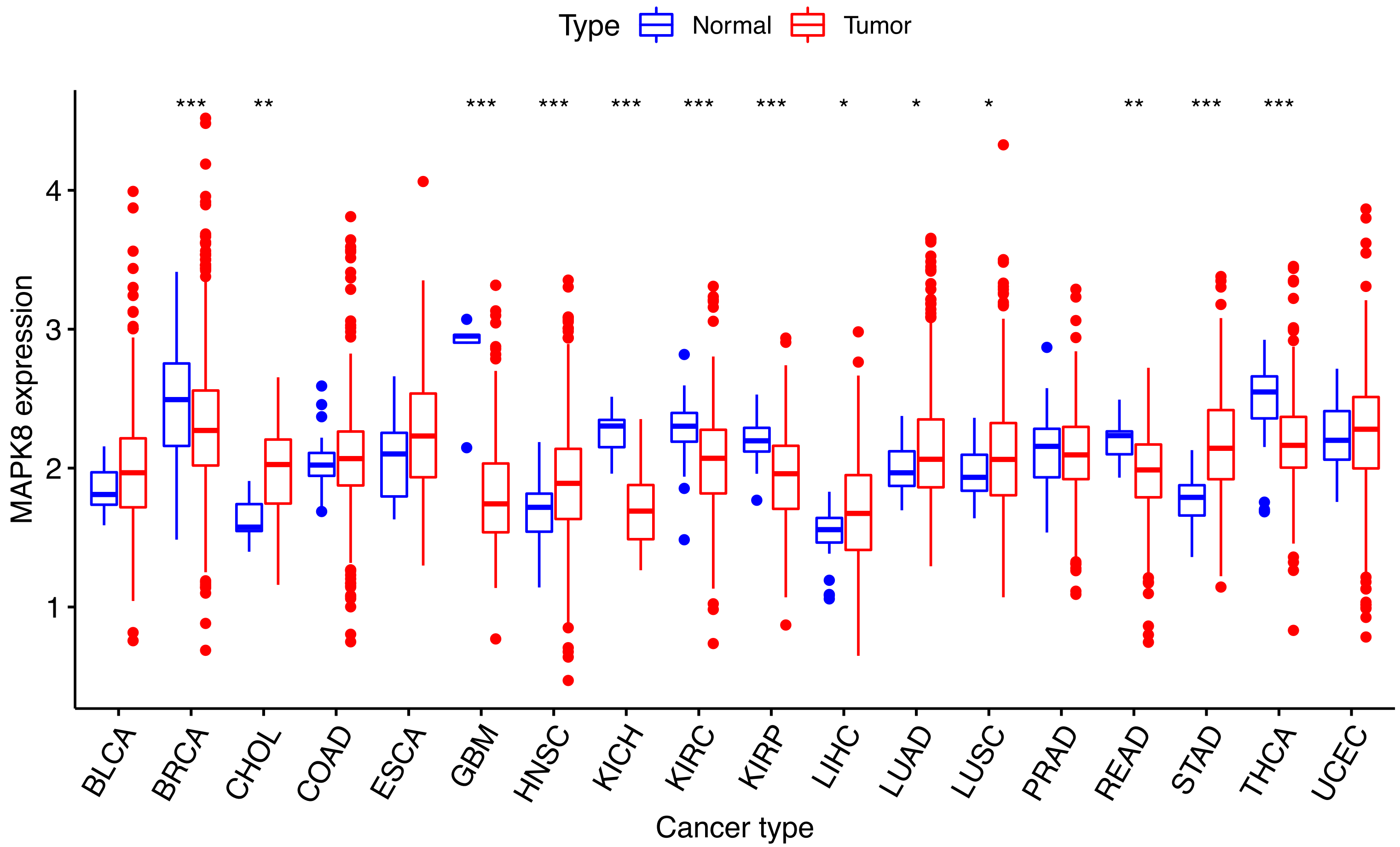

Type
Normal
Tumor
MAPK8 expression
4
3
2
1
*** ** *** *** *** *** *** * * * ** *** ***
BLCA
BRCA
CHOL
COAD
ESCA
GBM
HNSC
KICH
KIRC
KIRP
LIHC
LUAD
LUSC
PRAD
READ
STAD
THCA
UCEC
Cancer type

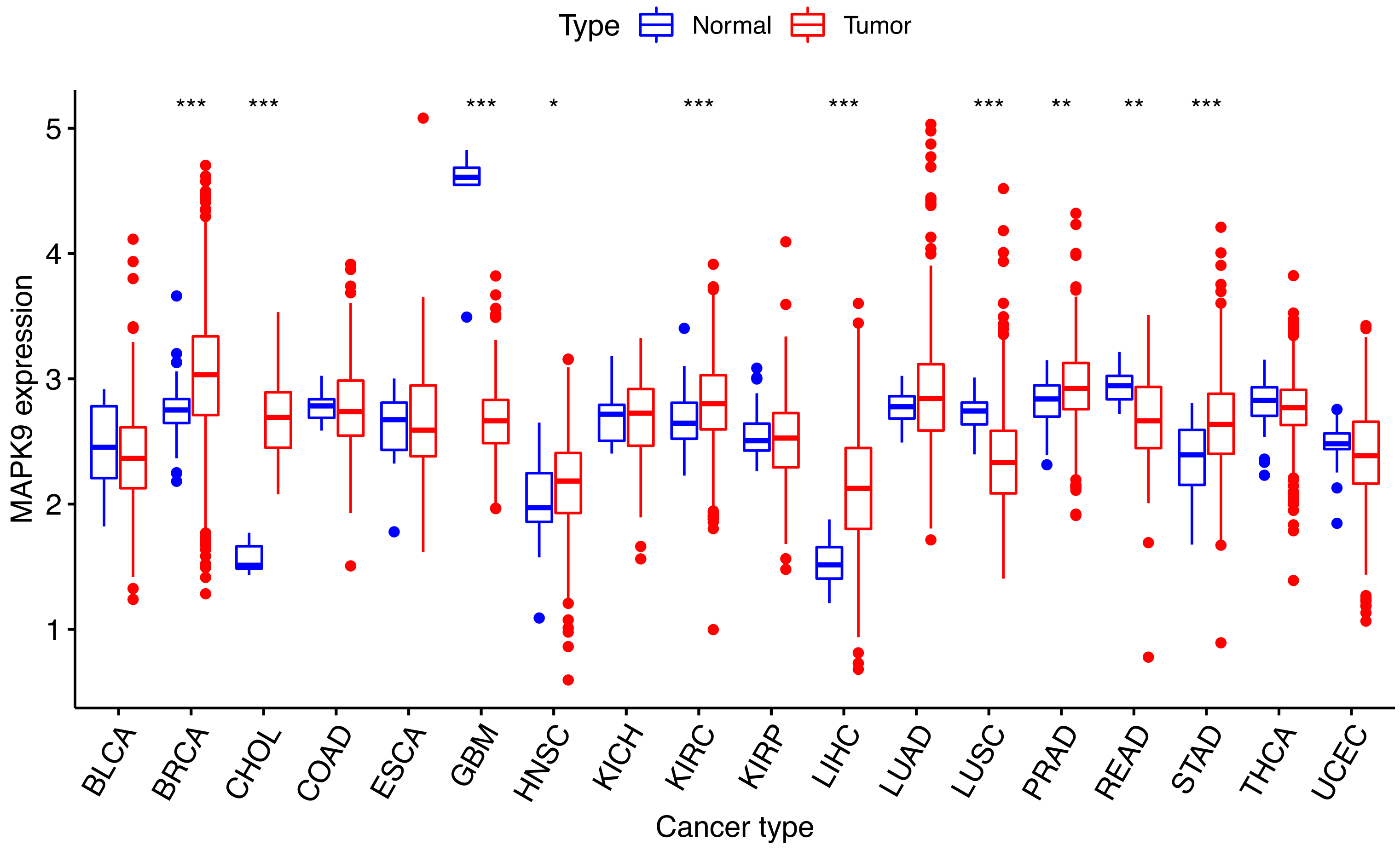

Type
Normal
Tumor
MAPK9 expression
Cancer type
5
4
3
2
1
***
***
***
*
***
***
***
**
**
***
BLCA
BRCA
CHOL
COAD
ESCA
GBM
HNSC
KICH
KIRC
KIRP
LIHC
LUAD
LUSC
PRAD
READ
STAD
THCA
UCEC

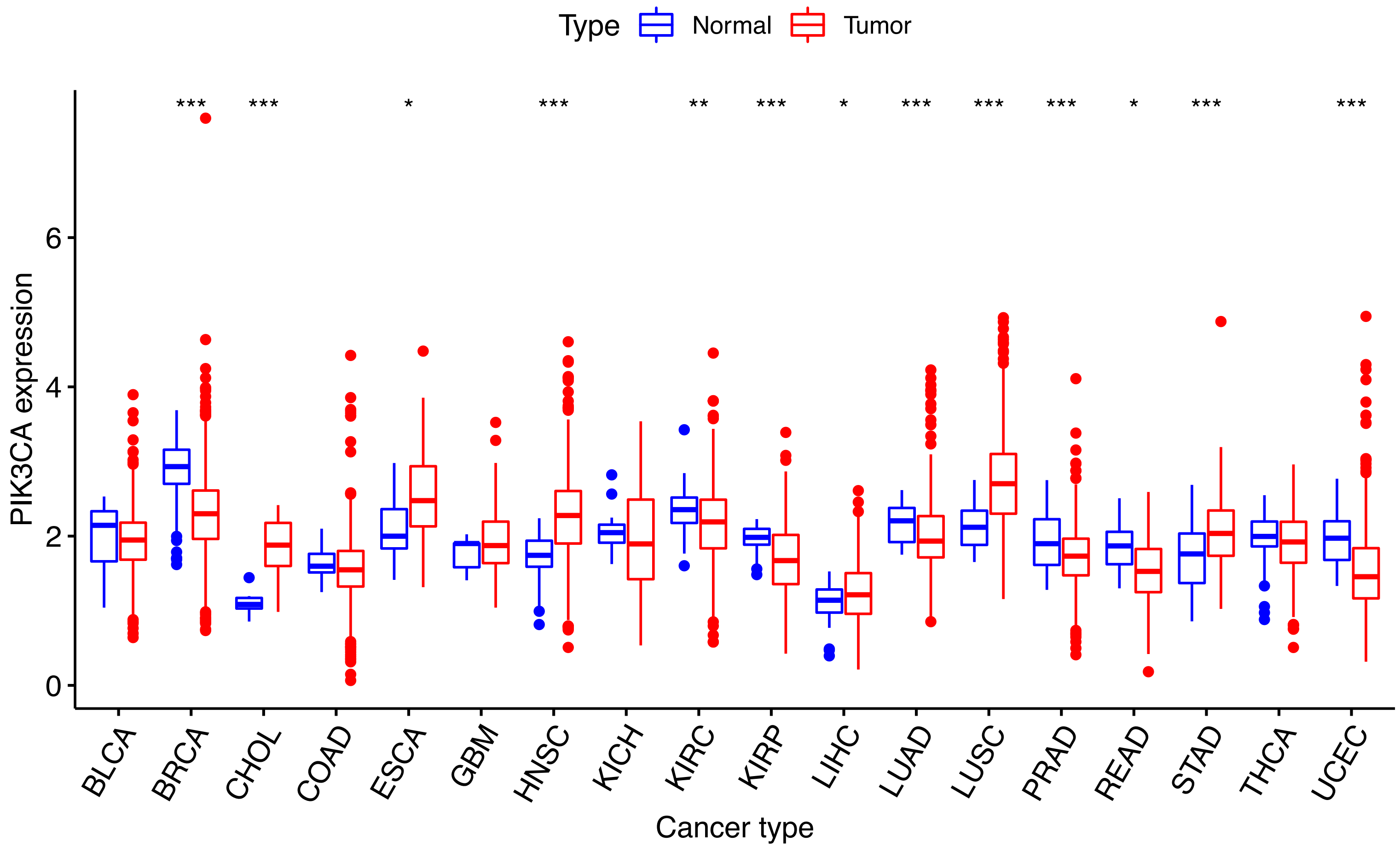
Type
Normal
Tumor
PIK3CA expression
0
2
4
6
***
***
*
***
**
***
*
***
***
***
*
***
***
BLCA
BRCA
CHOL
COAD
ESCA
GBM
HNSC
KICH
KIRC
KIRP
LIHC
LUAD
LUSC
PRAD
READ
STAD
THCA
UCEC
Cancer type

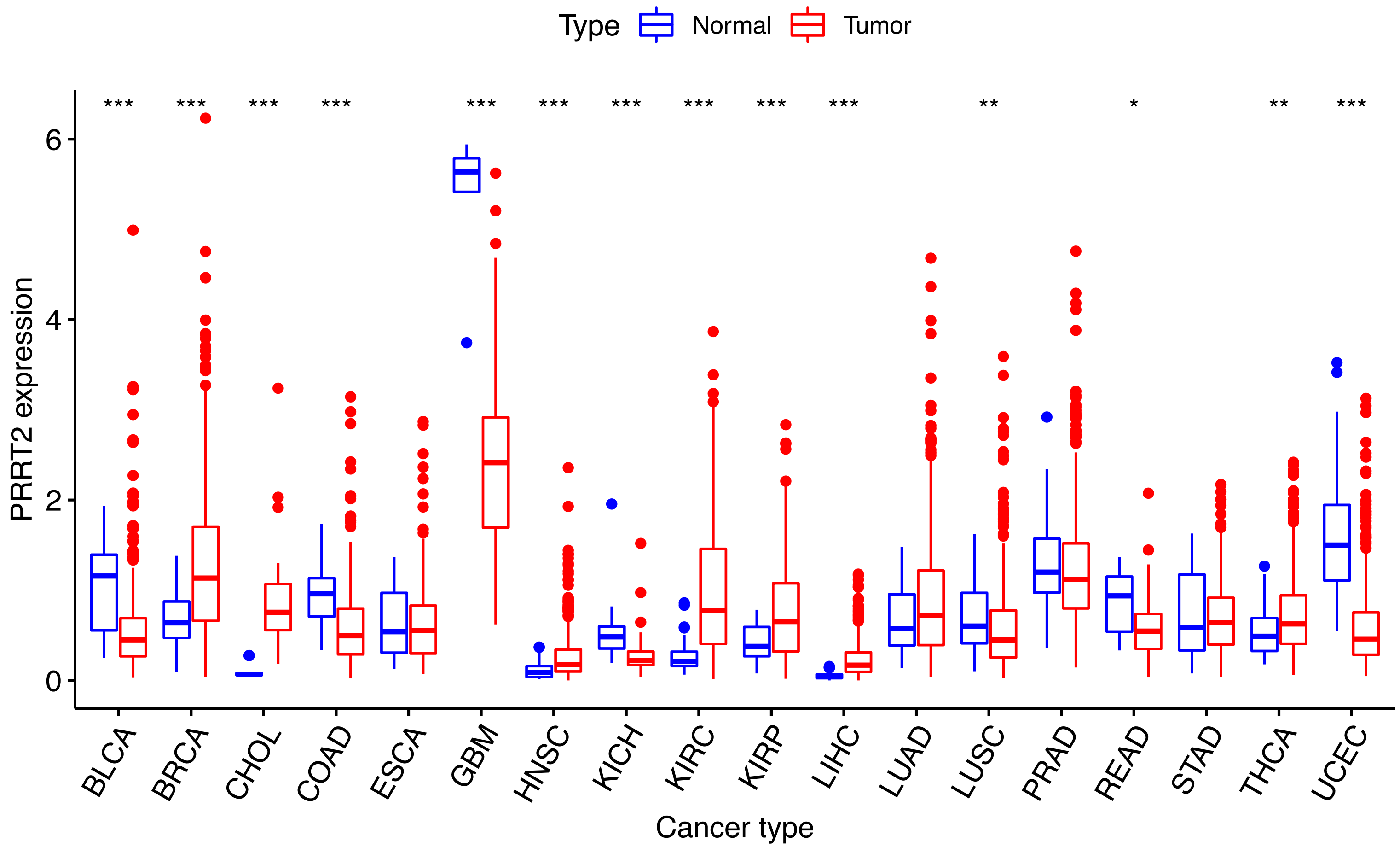

Type
Normal
Tumor
PRRT2 expression
0
2
4
6
*** *** *** *** *** *** *** *** *** *** ** * ** ***
BLCA
BRCA
CHOL
COAD
ESCA
GBM
HNSC
KICH
KIRC
KIRP
LIHC
LUAD
LUSC
PRAD
READ
STAD
THCA
UCEC
Cancer type

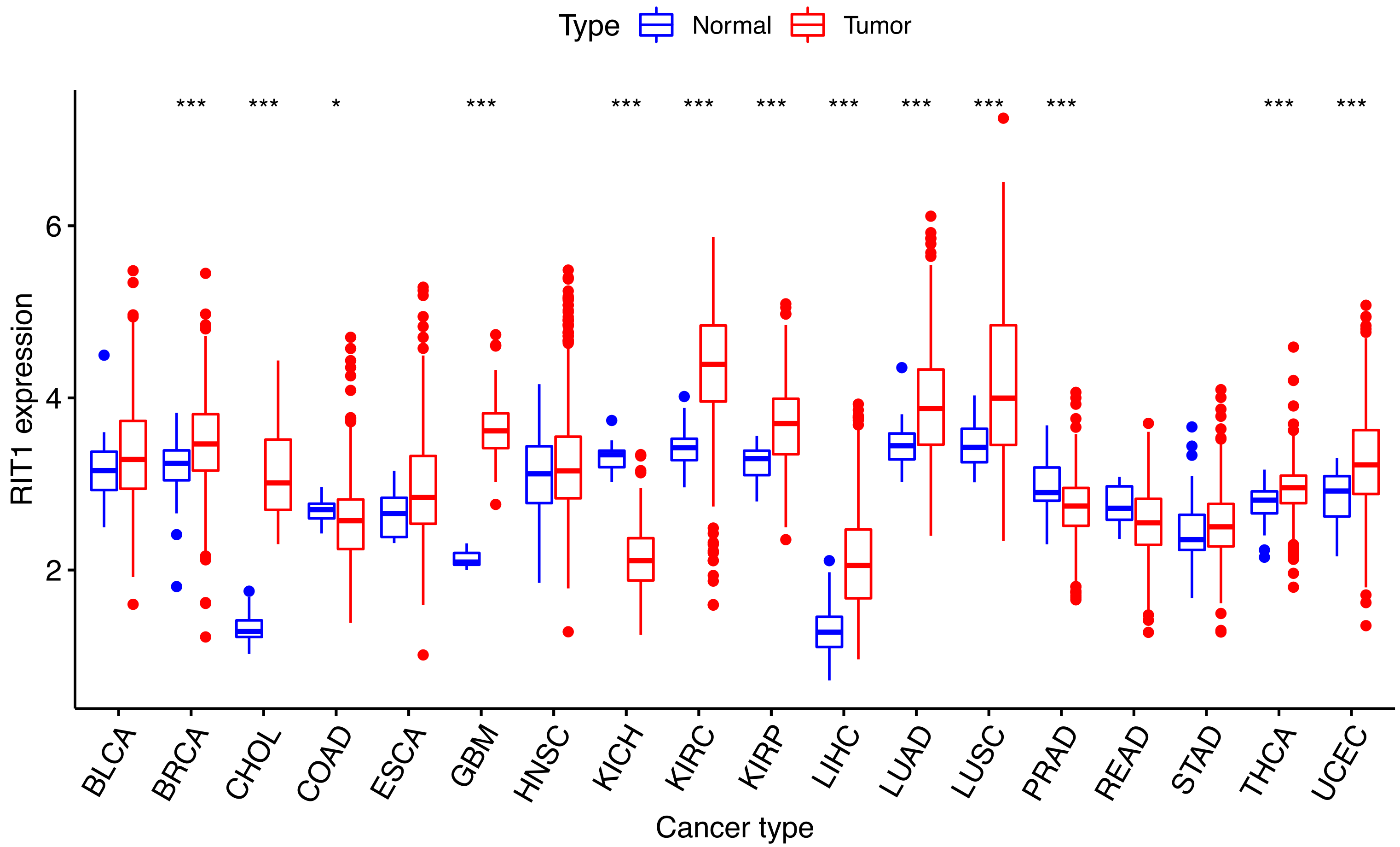

Type
Normal
Tumor
RIT1 expression
6
4
2
***
***
*
***
***
***
***
***
***
***
***
***
***
BLCA
BRCA
CHOL
COAD
ESCA
GBM
HNSC
KICH
KIRC
KIRP
LIHC
LUAD
LUSC
PRAD
READ
STAD
THCA
UCEC
Cancer type
