# Supplementary material for: Machine Learning Identifies Pan-Cancer Landscape of Nrf2 Oxidative Stress Response Pathway-Related Genes
Source: Oxid Med Cell Longev. 2022 Feb 17;2022:8450087. doi: 10.1155/2022/8450087 (PMC8886747; doi:10.1155/2022/8450087)
Supplement: Supplementary 2 — Supplementary Figure 2: the correlation between Nrf2 pathway-related genes and immunity across 33 cancer types. [file 8450087.f2.pdf]

Supplementary figure 2

The correlation between Nrf2 pathway-related genes and immunity across 33 cancer types.

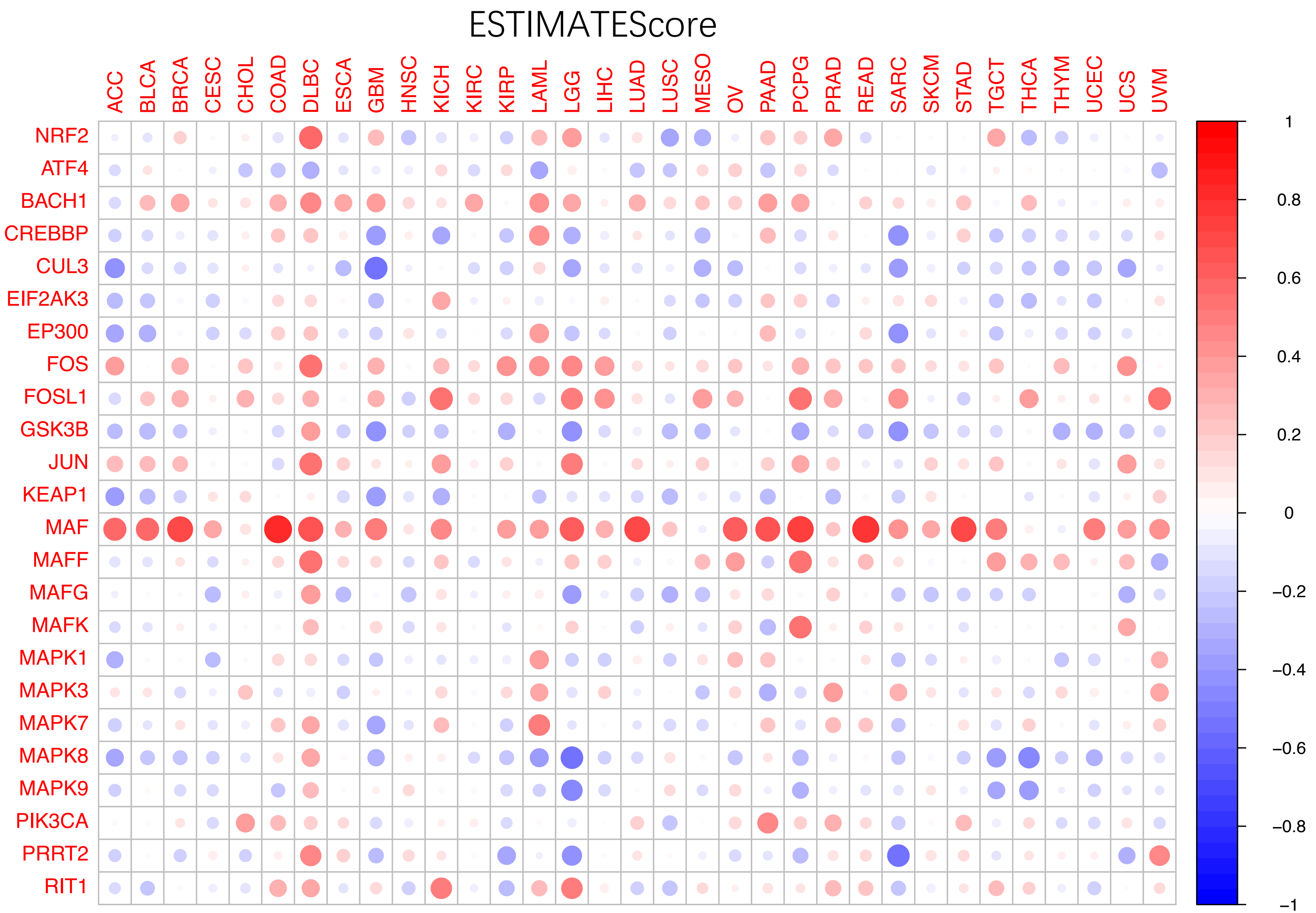

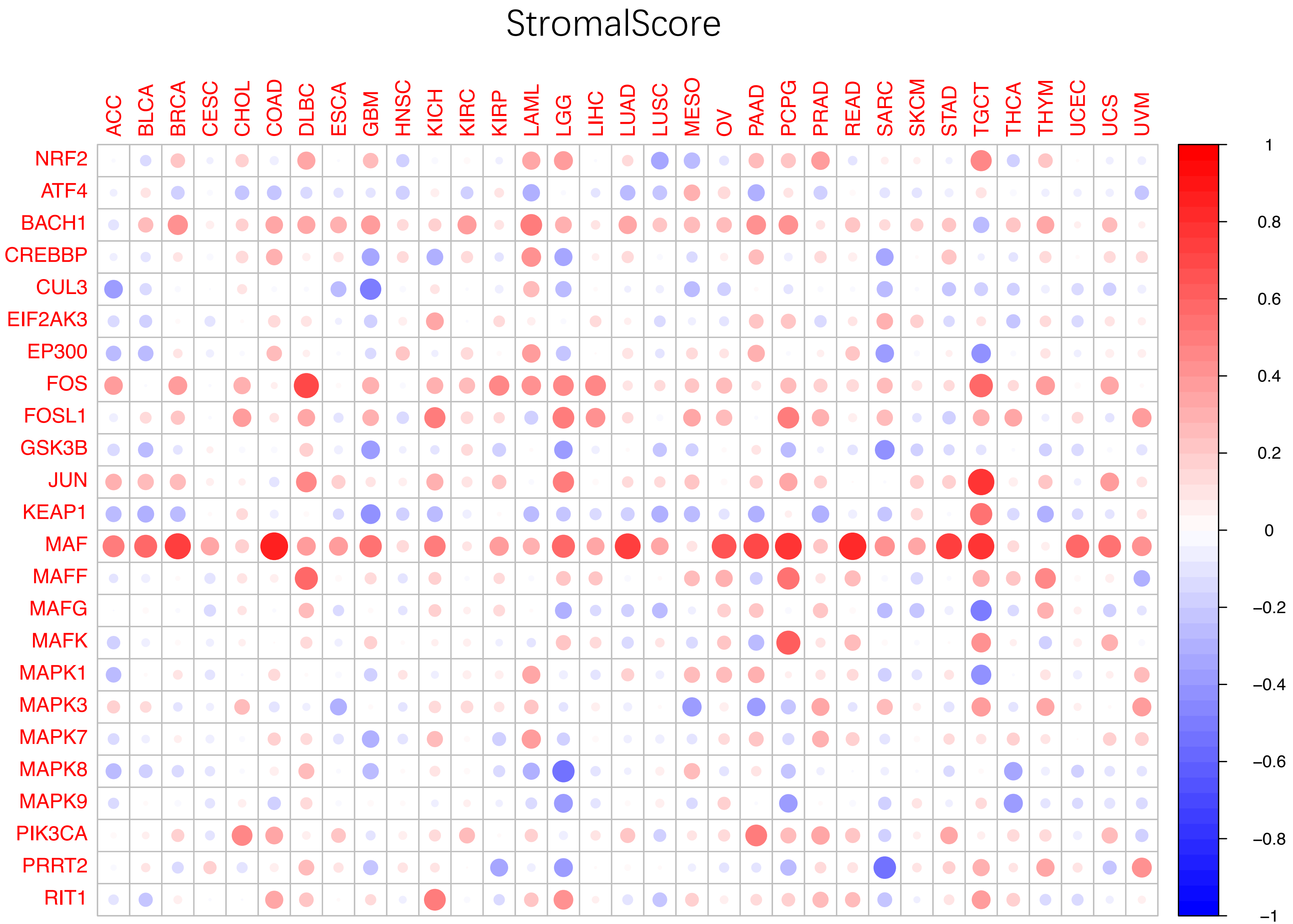

StromalScore
ACC
BLCA
BRCA
CESC
CHOL
COAD
DLBC
ESCA
GBM
HNSC
KICH
KIRC
KIRP
LAML
LGG
LIHC
LUAD
LUSC
MESO
OV
PAAD
PCPG
PRAD
READ
SARC
SKCM
STAD
TGCT
THCA
THYM
UCEC
UCS
UVM
NRF2
ATF4
BACH1
CREBBP
CUL3
EIF2AK3
EP300
FOS
FOSL1
GSK3B
JUN
KEAP1
MAF
MAFF
MAFG
MAFK
MAPK1
MAPK3
MAPK7
MAPK8
MAPK9
PIK3CA
PRRT2
RIT1
1
0.8
0.6
0.4
0.2
0
−0.2
−0.4
−0.6
−0.8
−1

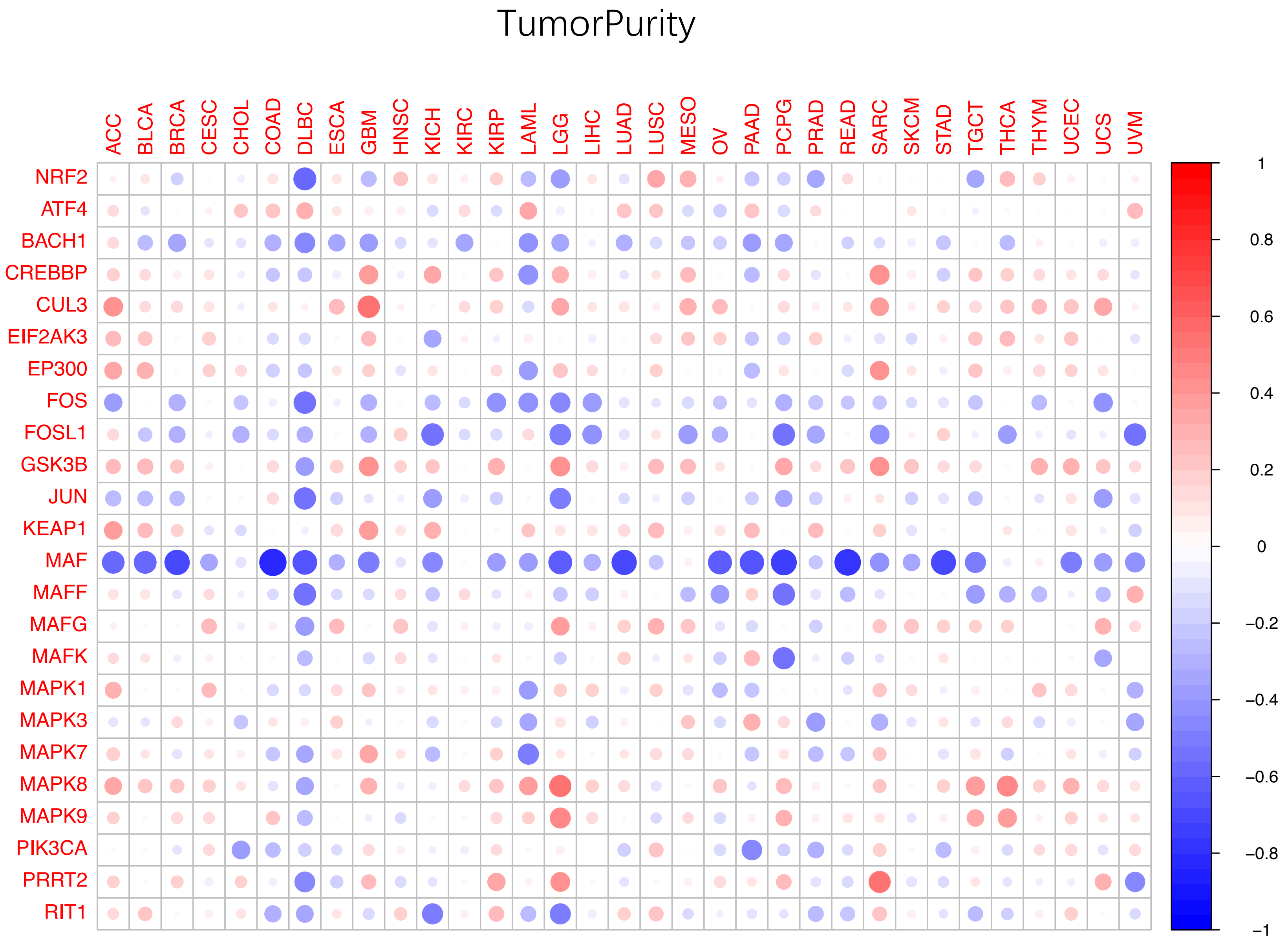

TumorPurity
ACC
BLCA
BRCA
CESC
CHOL
COAD
DLBC
ESCA
GBM
HNSC
KICH
KIRC
KIRP
LAML
LGG
LIHC
LUAD
LUSC
MESO
OV
PAAD
PCPG
PRAD
READ
SARC
SKCM
STAD
TGCT
THCA
THYM
UCEC
UCS
UVM
NRF2
ATF4
BACH1
CREBBP
CUL3
EIF2AK3
EP300
FOS
FOSL1
GSK3B
JUN
KEAP1
MAF
MAFF
MAFG
MAFK
MAPK1
MAPK3
MAPK7
MAPK8
MAPK9
PIK3CA
PRRT2
RIT1
1
0.8
0.6
0.4
0.2
0
−0.2
−0.4
−0.6
−0.8
−1

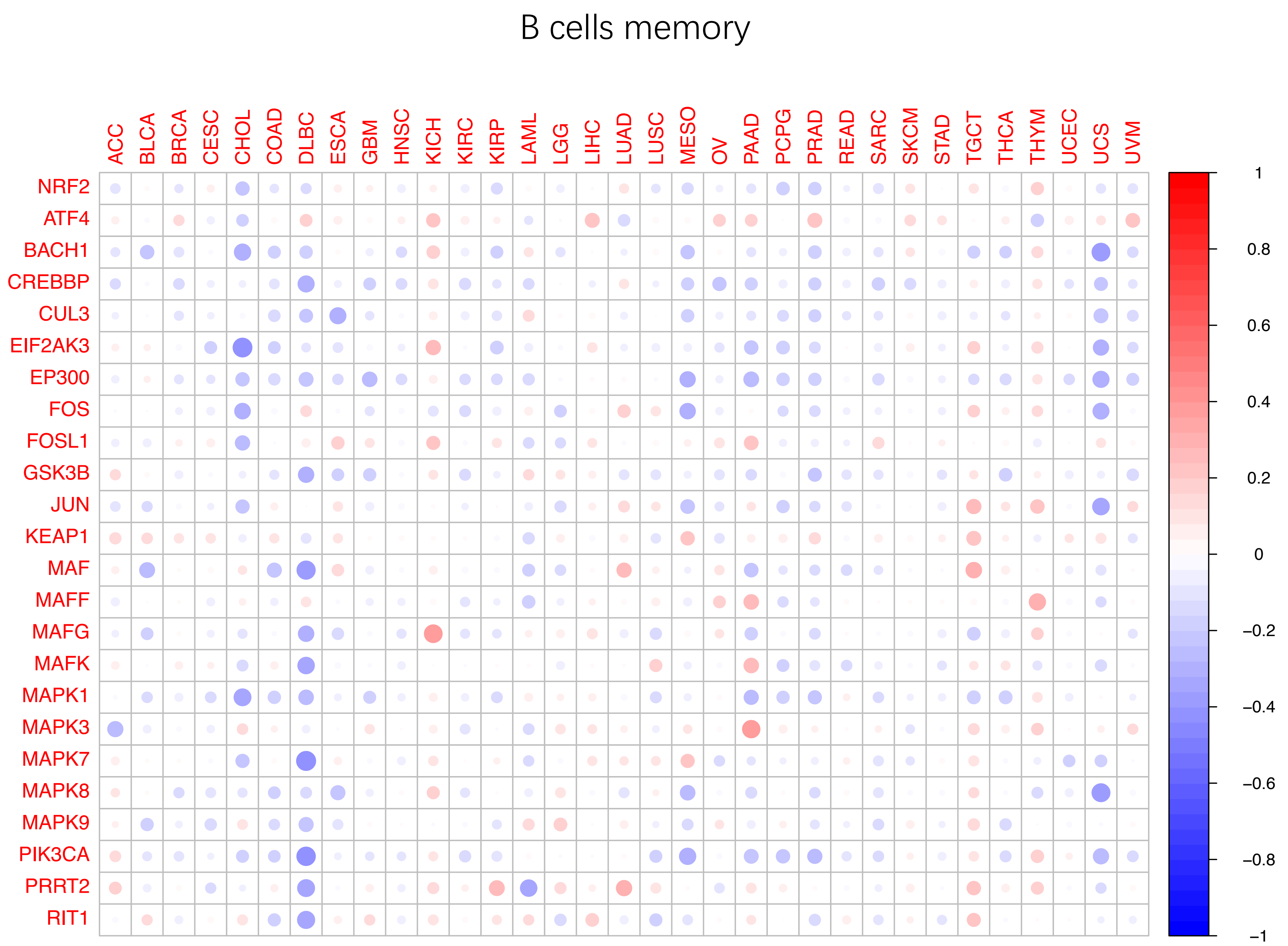
B cells memory
ACC
BLCA
BRCA
CESC
CHOL
COAD
DLBC
ESCA
GBM
HNSC
KICH
KIRC
KIRP
LAML
LGG
LIHC
LUAD
LUSC
MESO
OV
PAAD
PCPG
PRAD
READ
SARC
SKCM
STAD
TGCT
THCA
THYM
UCEC
UCS
UVM
NRF2
ATF4
BACH1
CREBBP
CUL3
EIF2AK3
EP300
FOS
FOSL1
GSK3B
JUN
KEAP1
MAF
MAFF
MAFG
MAFK
MAPK1
MAPK3
MAPK7
MAPK8
MAPK9
PIK3CA
PRRT2
RIT1
1
0.8
0.6
0.4
0.2
0
−0.2
−0.4
−0.6
−0.8
−1

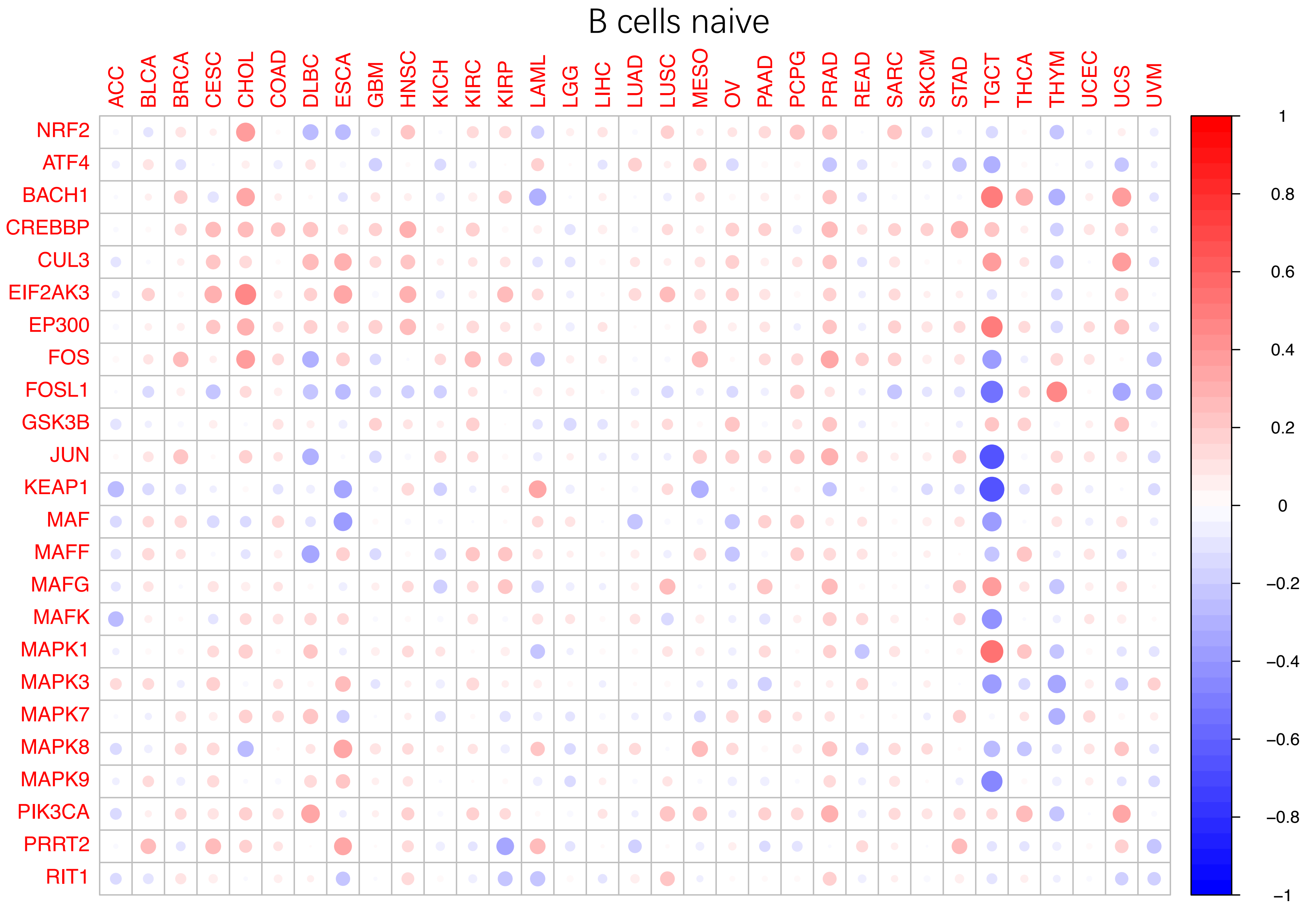

B cells naive
ACC
BLCA
BRCA
CESC
CHOL
COAD
DLBC
ESCA
GBM
HNSC
KICH
KIRC
KIRP
LAML
LGG
LIHC
LUAD
LUSC
MESO
OV
PAAD
PCPG
PRAD
READ
SARC
SKCM
STAD
TGCT
THCA
THYM
UCEC
UCS
UVM
NRF2
ATF4
BACH1
CREBBP
CUL3
EIF2AK3
EP300
FOS
FOSL1
GSK3B
JUN
KEAP1
MAF
MAFF
MAFG
MAFK
MAPK1
MAPK3
MAPK7
MAPK8
MAPK9
PIK3CA
PRRT2
RIT1
1
0.8
0.6
0.4
0.2
0
−0.2
−0.4
−0.6
−0.8
−1

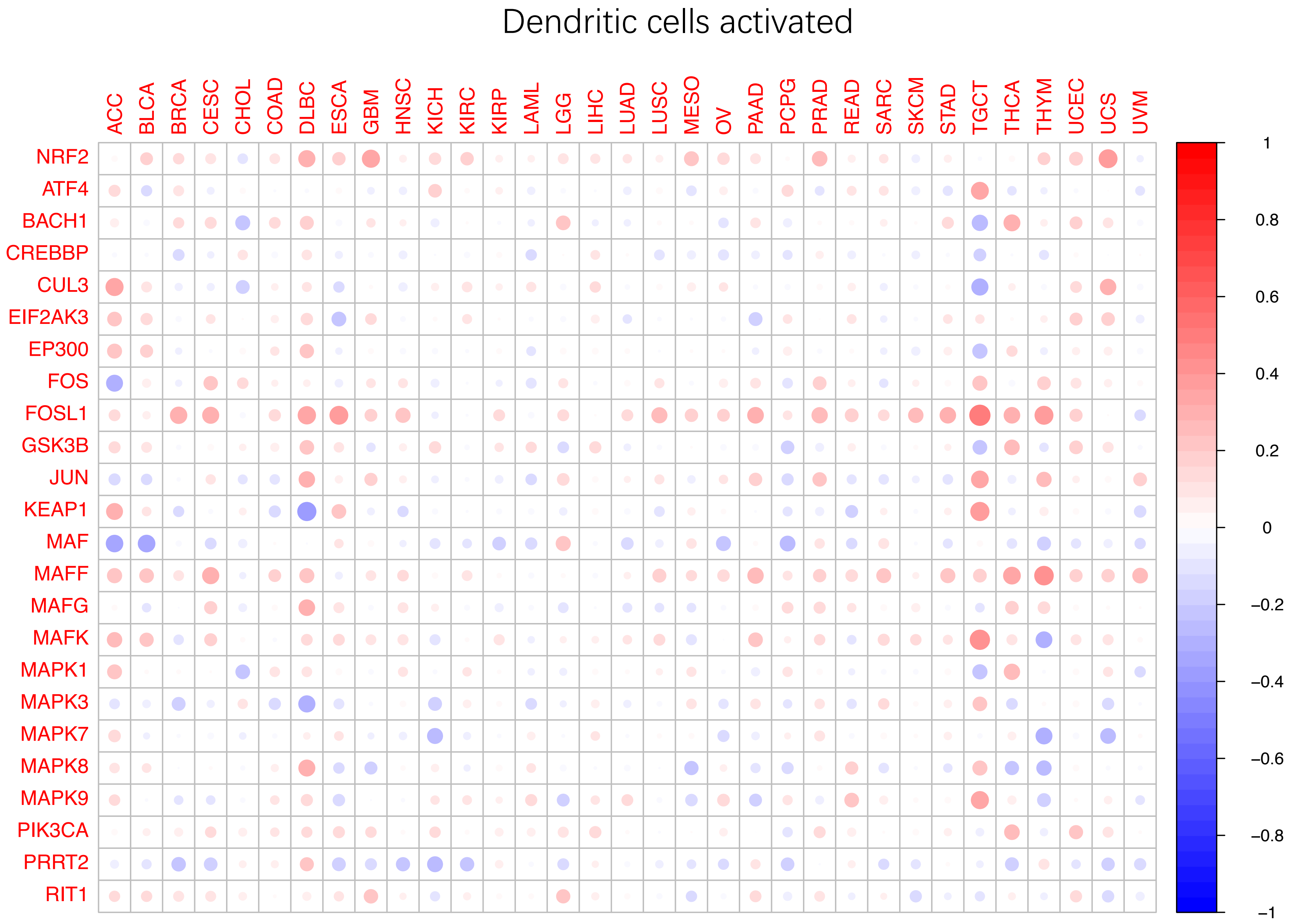
Dendritic cells activated
ACC
BLCA
BRCA
CESC
CHOL
COAD
DLBC
ESCA
GBM
HNSC
KICH
KIRC
KIRP
LAML
LGG
LIHC
LUAD
LUSC
MESO
OV
PAAD
PCPG
PRAD
READ
SARC
SKCM
STAD
TGCT
THCA
THYM
UCEC
UCS
UVM
NRF2
ATF4
BACH1
CREBBP
CUL3
EIF2AK3
EP300
FOS
FOSL1
GSK3B
JUN
KEAP1
MAF
MAFF
MAFG
MAFK
MAPK1
MAPK3
MAPK7
MAPK8
MAPK9
PIK3CA
PRRT2
RIT1
1
0.8
0.6
0.4
0.2
0
−0.2
−0.4
−0.6
−0.8
−1

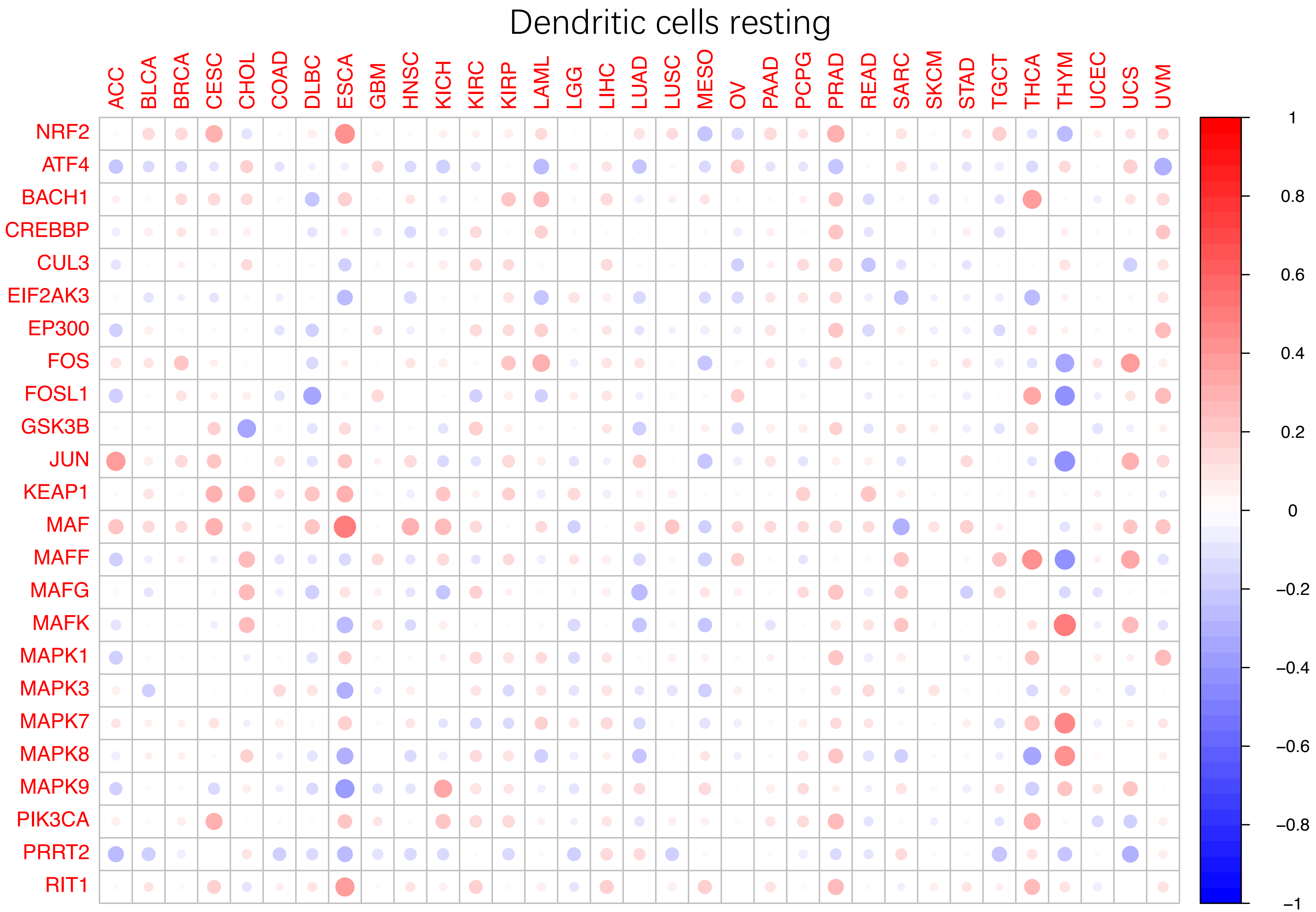
Dendritic cells resting
ACC
BLCA
BRCA
CESC
CHOL
COAD
DLBC
ESCA
GBM
HNSC
KICH
KIRC
KIRP
LAML
LGG
LIHC
LUAD
LUSC
MESO
OV
PAAD
PCPG
PRAD
READ
SARC
SKCM
STAD
TGCT
THCA
THYM
UCEC
UCS
UVM
NRF2
ATF4
BACH1
CREBBP
CUL3
EIF2AK3
EP300
FOS
FOSL1
GSK3B
JUN
KEAP1
MAF
MAFF
MAFG
MAFK
MAPK1
MAPK3
MAPK7
MAPK8
MAPK9
PIK3CA
PRRT2
RIT1
1
0.8
0.6
0.4
0.2
0
−0.2
−0.4
−0.6
−0.8
−1

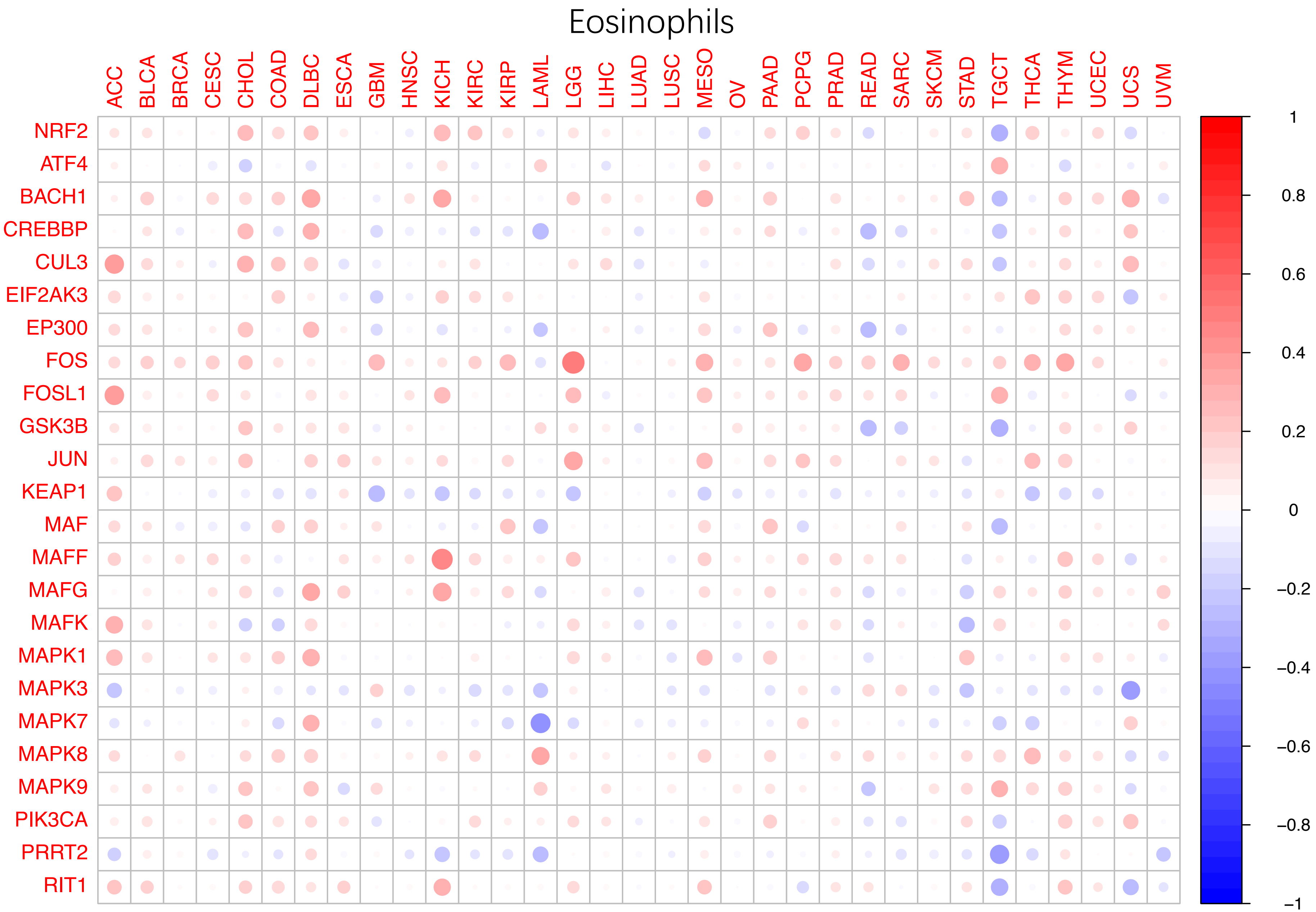

Eosinophils
ACC
BLCA
BRCA
CESC
CHOL
COAD
DLBC
ESCA
GBM
HNSC
KICH
KIRC
KIRP
LAML
LGG
LIHC
LUAD
LUSC
MESO
OV
PAAD
PCPG
PRAD
READ
SARC
SKCM
STAD
TGCT
THCA
THYM
UCEC
UCS
UVM
NRF2
ATF4
BACH1
CREBBP
CUL3
EIF2AK3
EP300
FOS
FOSL1
GSK3B
JUN
KEAP1
MAF
MAFF
MAFG
MAFK
MAPK1
MAPK3
MAPK7
MAPK8
MAPK9
PIK3CA
PRRT2
RIT1
1
0.8
0.6
0.4
0.2
0
−0.2
−0.4
−0.6
−0.8
−1

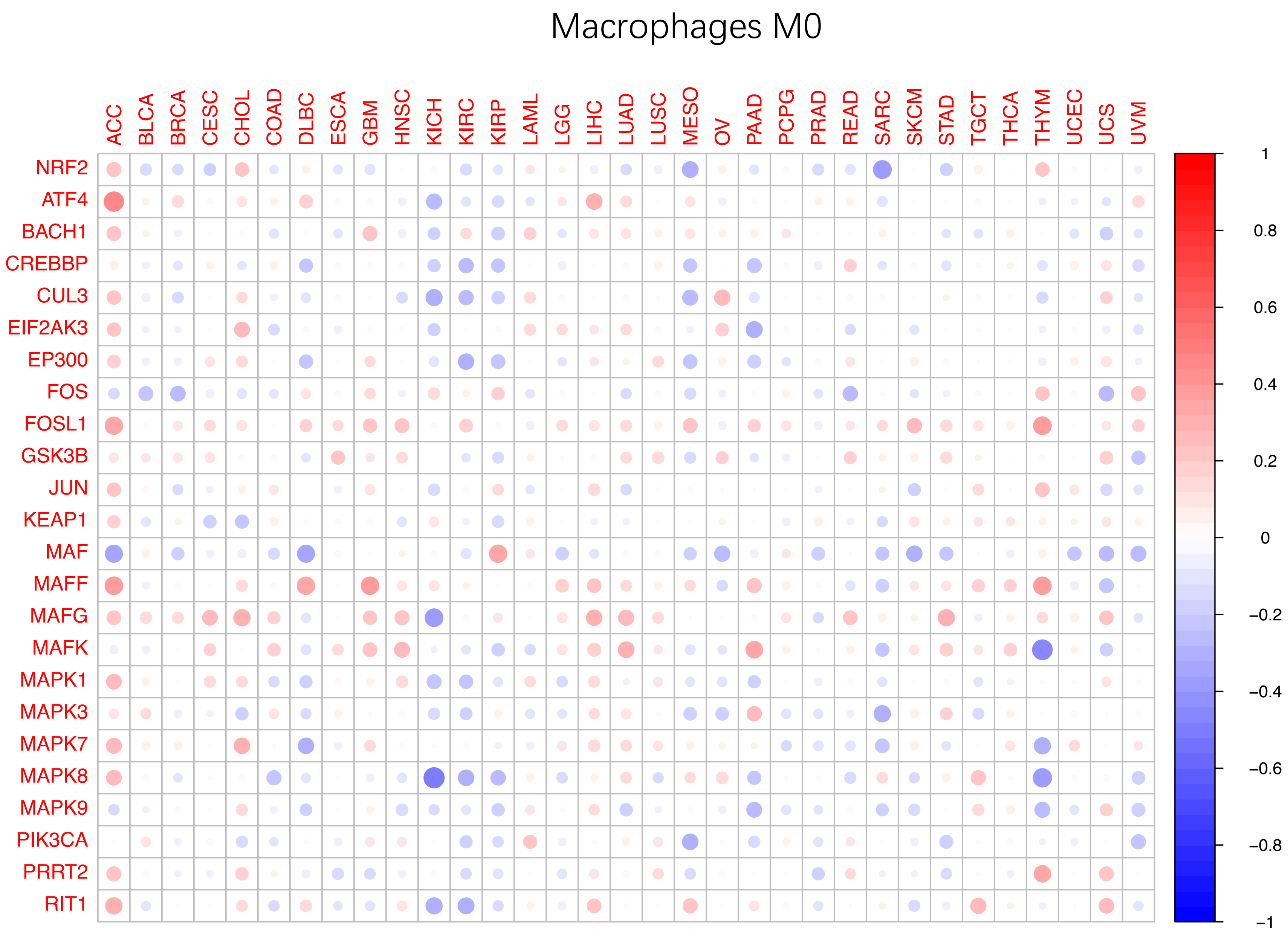
Macrophages M0
ACC
BLCA
BRCA
CESC
CHOL
COAD
DLBC
ESCA
GBM
HNSC
KICH
KIRC
KIRP
LAML
LGG
LIHC
LUAD
LUSC
MESO
OV
PAAD
PCPG
PRAD
READ
SARC
SKCM
STAD
TGCT
THCA
THYM
UCEC
UCS
UVM
NRF2
ATF4
BACH1
CREBBP
CUL3
EIF2AK3
EP300
FOS
FOSL1
GSK3B
JUN
KEAP1
MAF
MAFF
MAFG
MAFK
MAPK1
MAPK3
MAPK7
MAPK8
MAPK9
PIK3CA
PRRT2
RIT1
1
0.8
0.6
0.4
0.2
0
−0.2
−0.4
−0.6
−0.8
−1

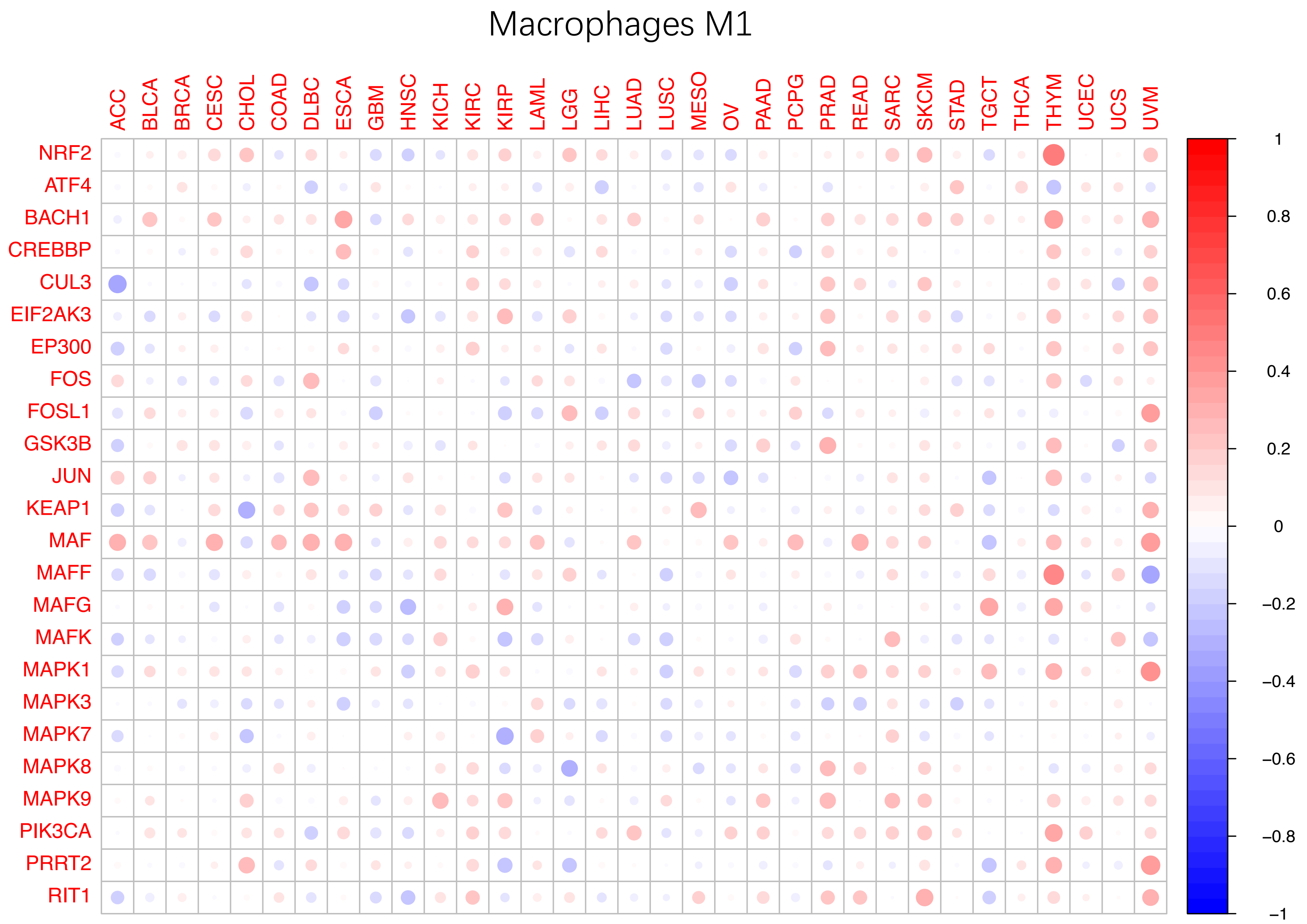
Macrophages M1
ACC
BLCA
BRCA
CESC
CHOL
COAD
DLBC
ESCA
GBM
HNSC
KICH
KIRC
KIRP
LAML
LGG
LIHC
LUAD
LUSC
MESO
OV
PAAD
PCPG
PRAD
READ
SARC
SKCM
STAD
TGCT
THCA
THYM
UCEC
UCS
UVM
NRF2
ATF4
BACH1
CREBBP
CUL3
EIF2AK3
EP300
FOS
FOSL1
GSK3B
JUN
KEAP1
MAF
MAFF
MAFG
MAFK
MAPK1
MAPK3
MAPK7
MAPK8
MAPK9
PIK3CA
PRRT2
RIT1
1
0.8
0.6
0.4
0.2
0
−0.2
−0.4
−0.6
−0.8
−1

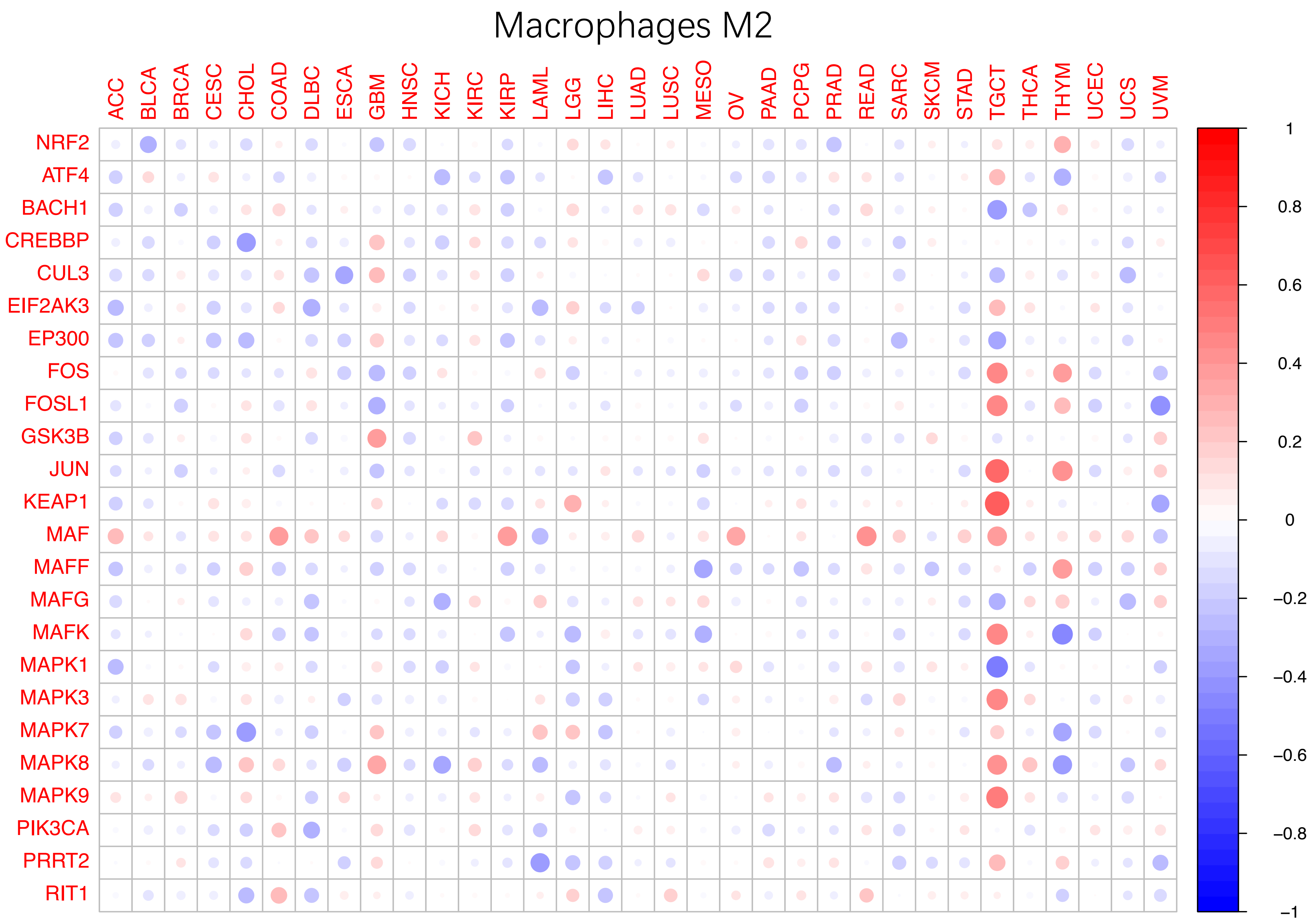
Macrophages M2
ACC
BLCA
BRCA
CESC
CHOL
COAD
DLBC
ESCA
GBM
HNSC
KICH
KIRC
KIRP
LAML
LGG
LIHC
LUAD
LUSC
MESO
OV
PAAD
PCPG
PRAD
READ
SARC
SKCM
STAD
TGCT
THCA
THYM
UCEC
UCS
UVM
NRF2
ATF4
BACH1
CREBBP
CUL3
EIF2AK3
EP300
FOS
FOSL1
GSK3B
JUN
KEAP1
MAF
MAFF
MAFG
MAFK
MAPK1
MAPK3
MAPK7
MAPK8
MAPK9
PIK3CA
PRRT2
RIT1
1
0.8
0.6
0.4
0.2
0
−0.2
−0.4
−0.6
−0.8
−1

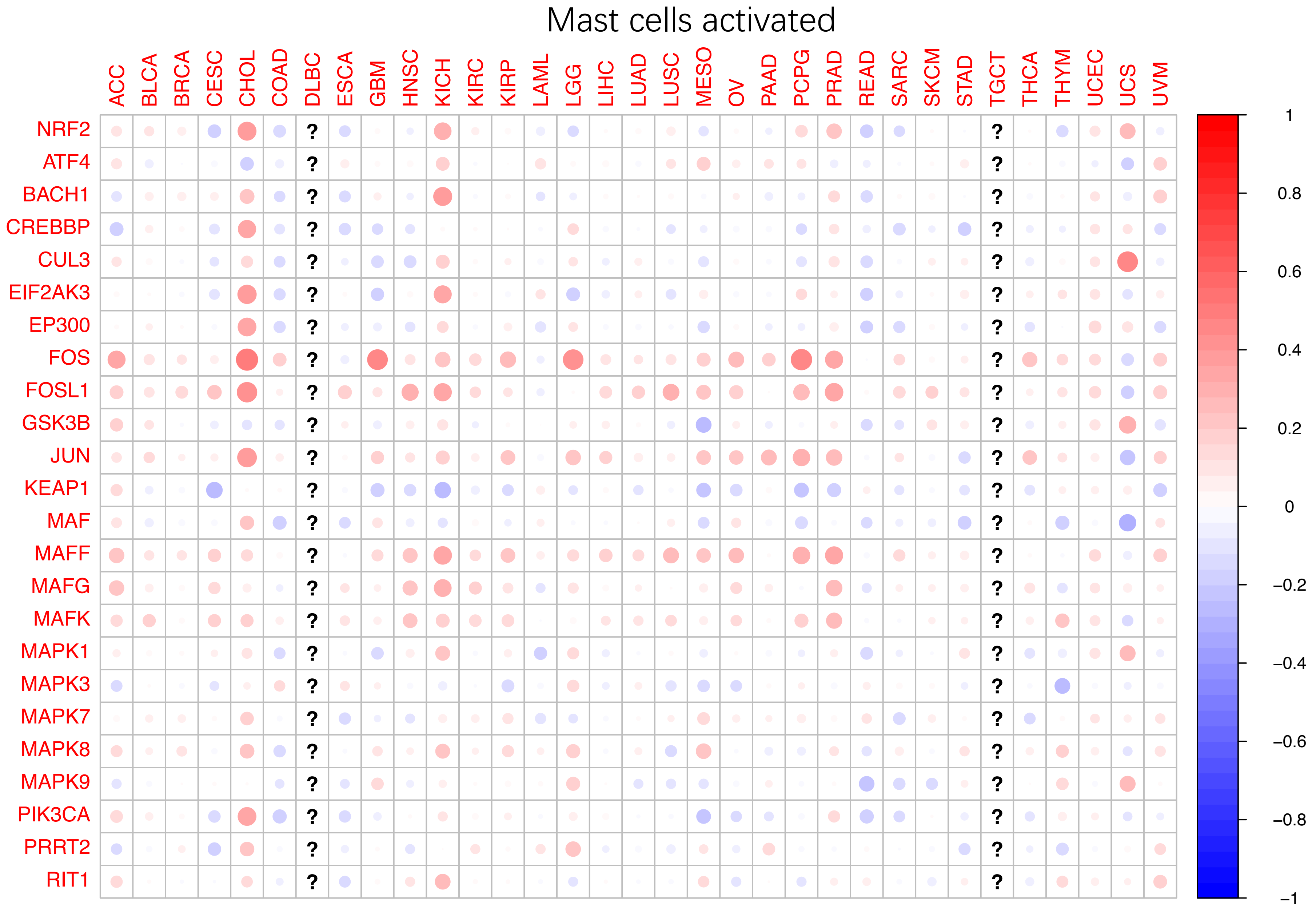

Mast cells activated
ACC
BLCA
BRCA
CESC
CHOL
COAD
DLBC
ESCA
GBM
HNSC
KICH
KIRC
KIRP
LAML
LGG
LIHC
LUAD
LUSC
MESO
OV
PAAD
PCPG
PRAD
READ
SARC
SKCM
STAD
TGCT
THCA
THYM
UCEC
UCS
UVM
NRF2
ATF4
BACH1
CREBBP
CUL3
EIF2AK3
EP300
FOS
FOSL1
GSK3B
JUN
KEAP1
MAF
MAFF
MAFG
MAFK
MAPK1
MAPK3
MAPK7
MAPK8
MAPK9
PIK3CA
PRRT2
RIT1
1
0.8
0.6
0.4
0.2
0
−0.2
−0.4
−0.6
−0.8
−1

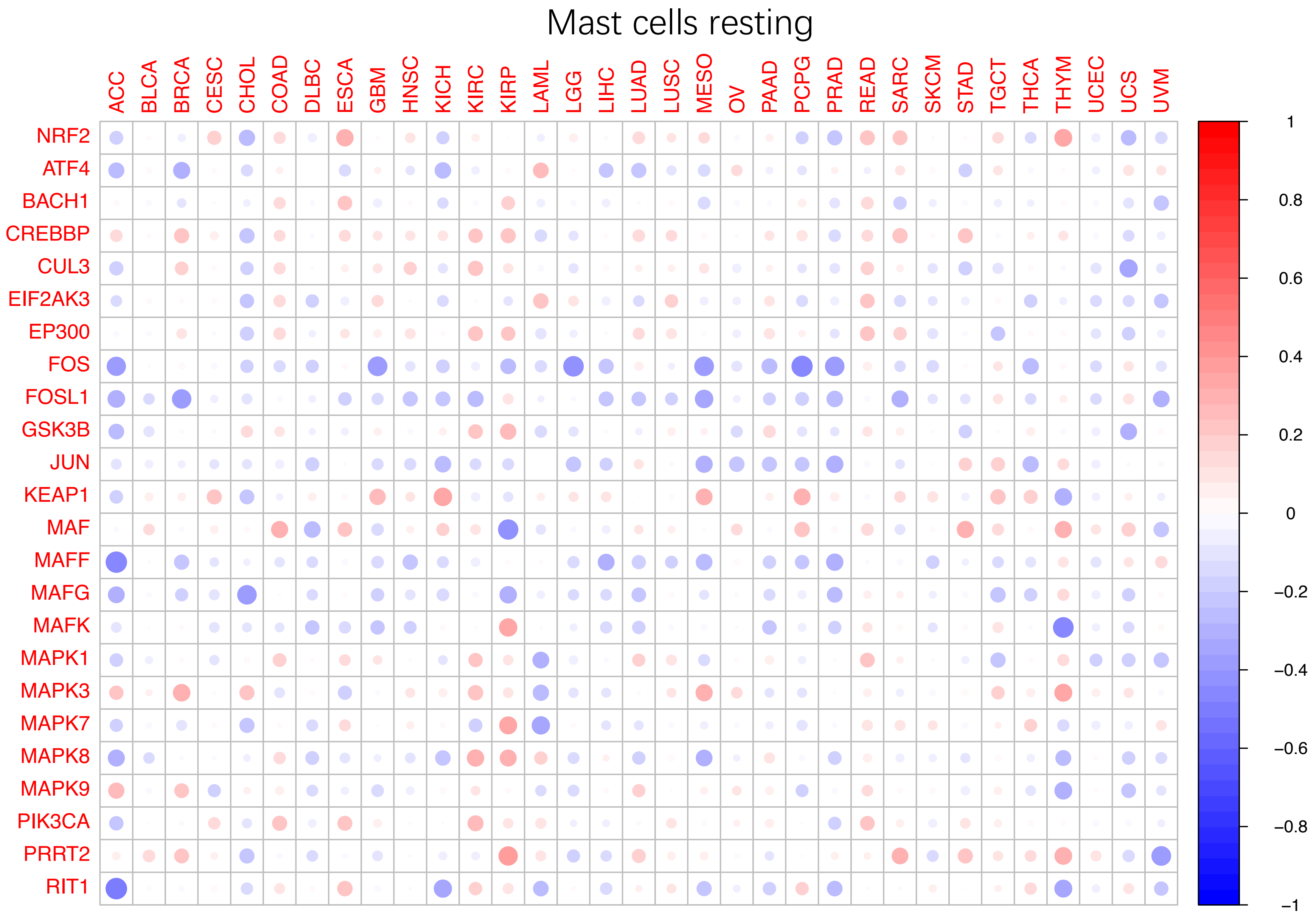

Mast cells resting
ACC BLCA BRCA CESC CHOL COAD DLBC ESCA GBM HNSC KICH KIRC KIRP LAML LGG LIHC LUAD LUSC MESO OV PAAD PCPG PRAD READ SARC SKCM STAD TGCT THCA THYM UCEC UCS UVM
NRF2
ATF4
BACH1
CREBBP
CUL3
EIF2AK3
EP300
FOS
FOSL1
GSK3B
JUN
KEAP1
MAF
MAFF
MAFG
MAFK
MAPK1
MAPK3
MAPK7
MAPK8
MAPK9
PIK3CA
PRRT2
RIT1
1
0.8
0.6
0.4
0.2
0
−0.2
−0.4
−0.6
−0.8
−1

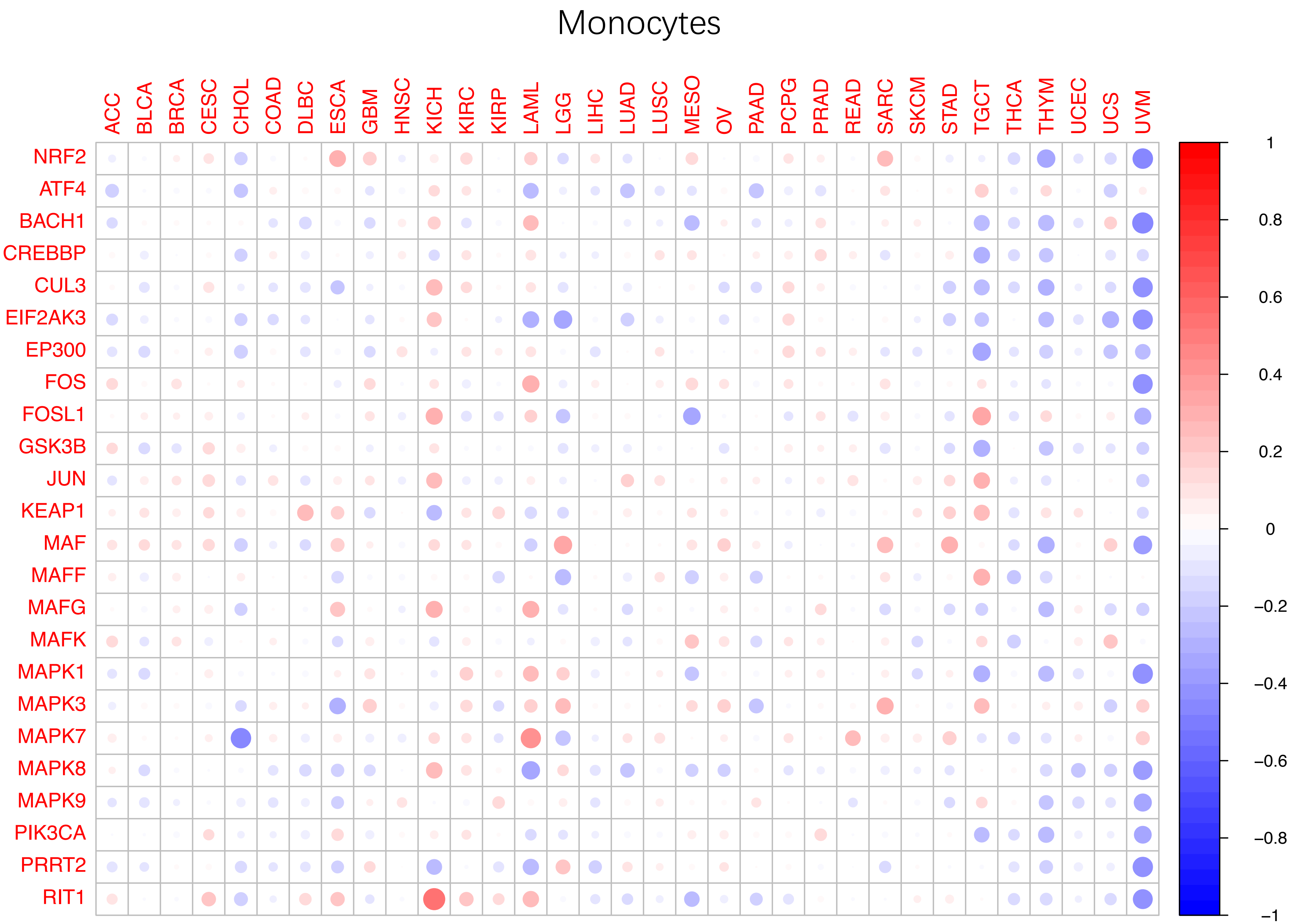
Monocytes
ACC
BLCA
BRCA
CESC
CHOL
COAD
DLBC
ESCA
GBM
HNSC
KICH
KIRC
KIRP
LAML
LGG
LIHC
LUAD
LUSC
MESO
OV
PAAD
PCPG
PRAD
READ
SARC
SKCM
STAD
TGCT
THCA
THYM
UCEC
UCS
UVM
NRF2
ATF4
BACH1
CREBBP
CUL3
EIF2AK3
EP300
FOS
FOSL1
GSK3B
JUN
KEAP1
MAF
MAFF
MAFG
MAFK
MAPK1
MAPK3
MAPK7
MAPK8
MAPK9
PIK3CA
PRRT2
RIT1
1
0.8
0.6
0.4
0.2
0
−0.2
−0.4
−0.6
−0.8
−1

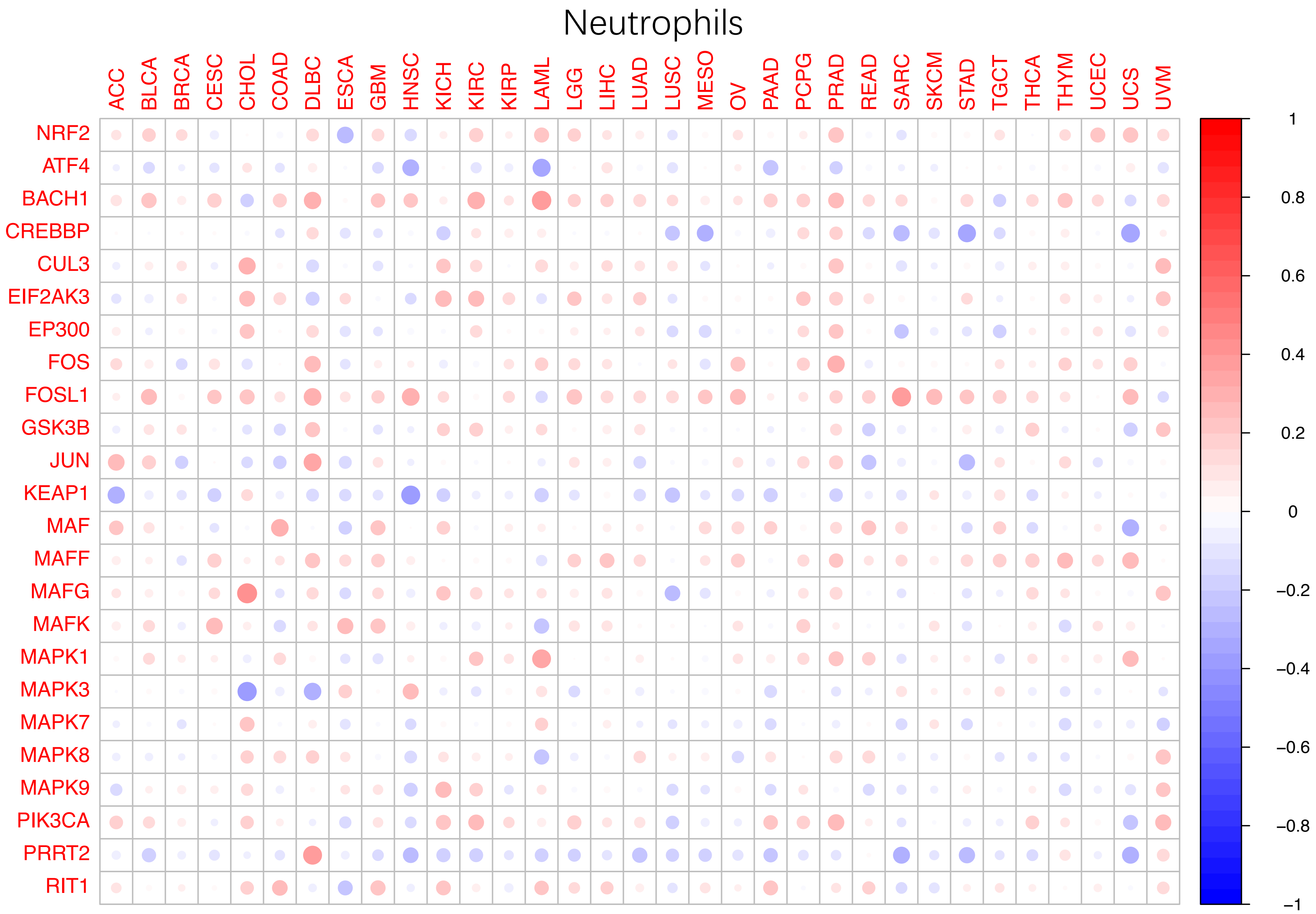
Neutrophils
ACC
BLCA
BRCA
CESC
CHOL
COAD
DLBC
ESCA
GBM
HNSC
KICH
KIRC
KIRP
LAML
LGG
LIHC
LUAD
LUSC
MESO
OV
PAAD
PCPG
PRAD
READ
SARC
SKCM
STAD
TGCT
THCA
THYM
UCEC
UCS
UVM
NRF2
ATF4
BACH1
CREBBP
CUL3
EIF2AK3
EP300
FOS
FOSL1
GSK3B
JUN
KEAP1
MAF
MAFF
MAFG
MAFK
MAPK1
MAPK3
MAPK7
MAPK8
MAPK9
PIK3CA
PRRT2
RIT1
1
0.8
0.6
0.4
0.2
0
−0.2
−0.4
−0.6
−0.8
−1

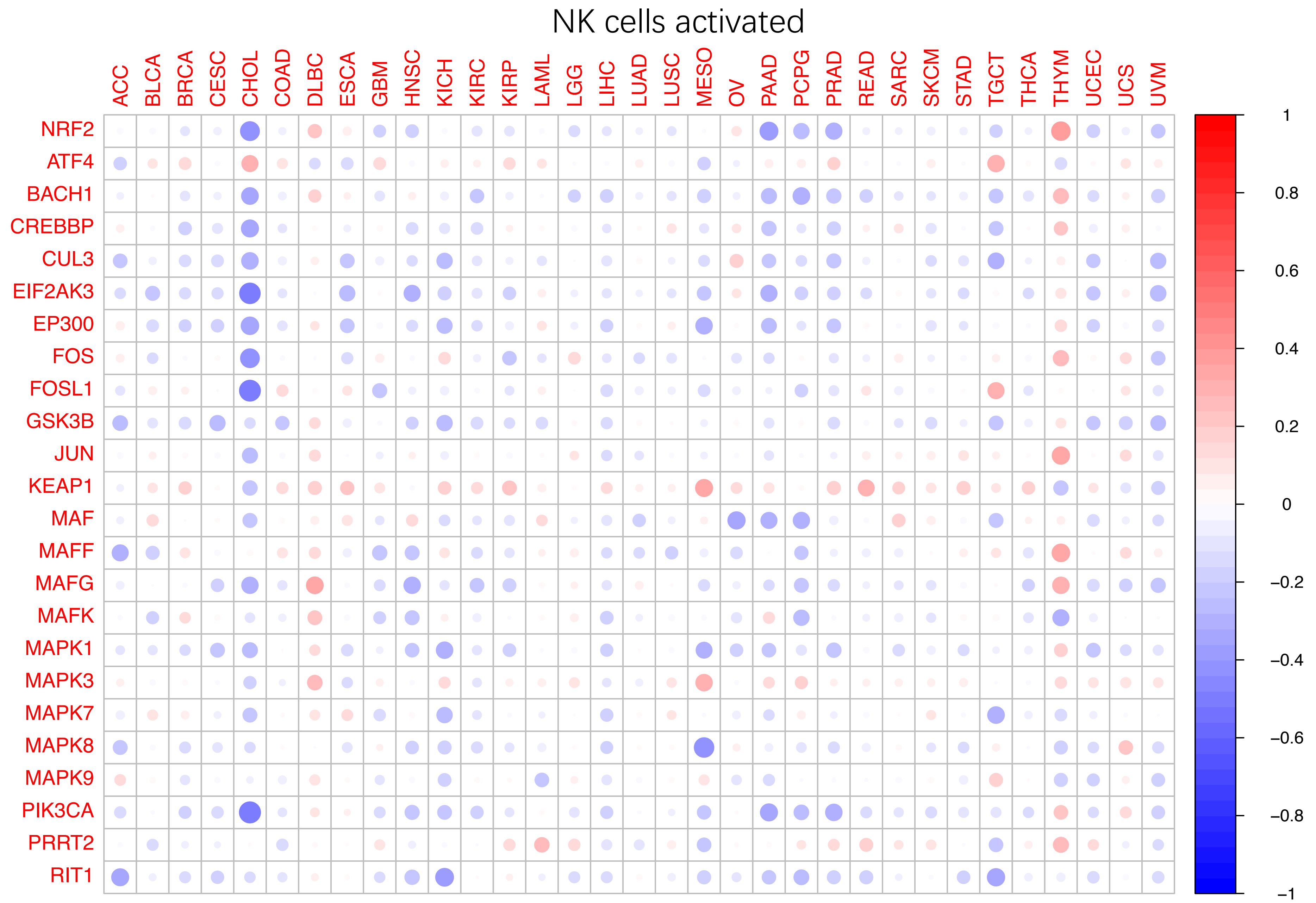
NK cells activated
ACC
BLCA
BRCA
CESC
CHOL
COAD
DLBC
ESCA
GBM
HNSC
KICH
KIRC
KIRP
LAML
LGG
LIHC
LUAD
LUSC
MESO
OV
PAAD
PCPG
PRAD
READ
SARC
SKCM
STAD
TGCT
THCA
THYM
UCEC
UCS
UVM
NRF2
ATF4
BACH1
CREBBP
CUL3
EIF2AK3
EP300
FOS
FOSL1
GSK3B
JUN
KEAP1
MAF
MAFF
MAFG
MAFK
MAPK1
MAPK3
MAPK7
MAPK8
MAPK9
PIK3CA
PRRT2
RIT1
1
0.8
0.6
0.4
0.2
0
−0.2
−0.4
−0.6
−0.8
−1

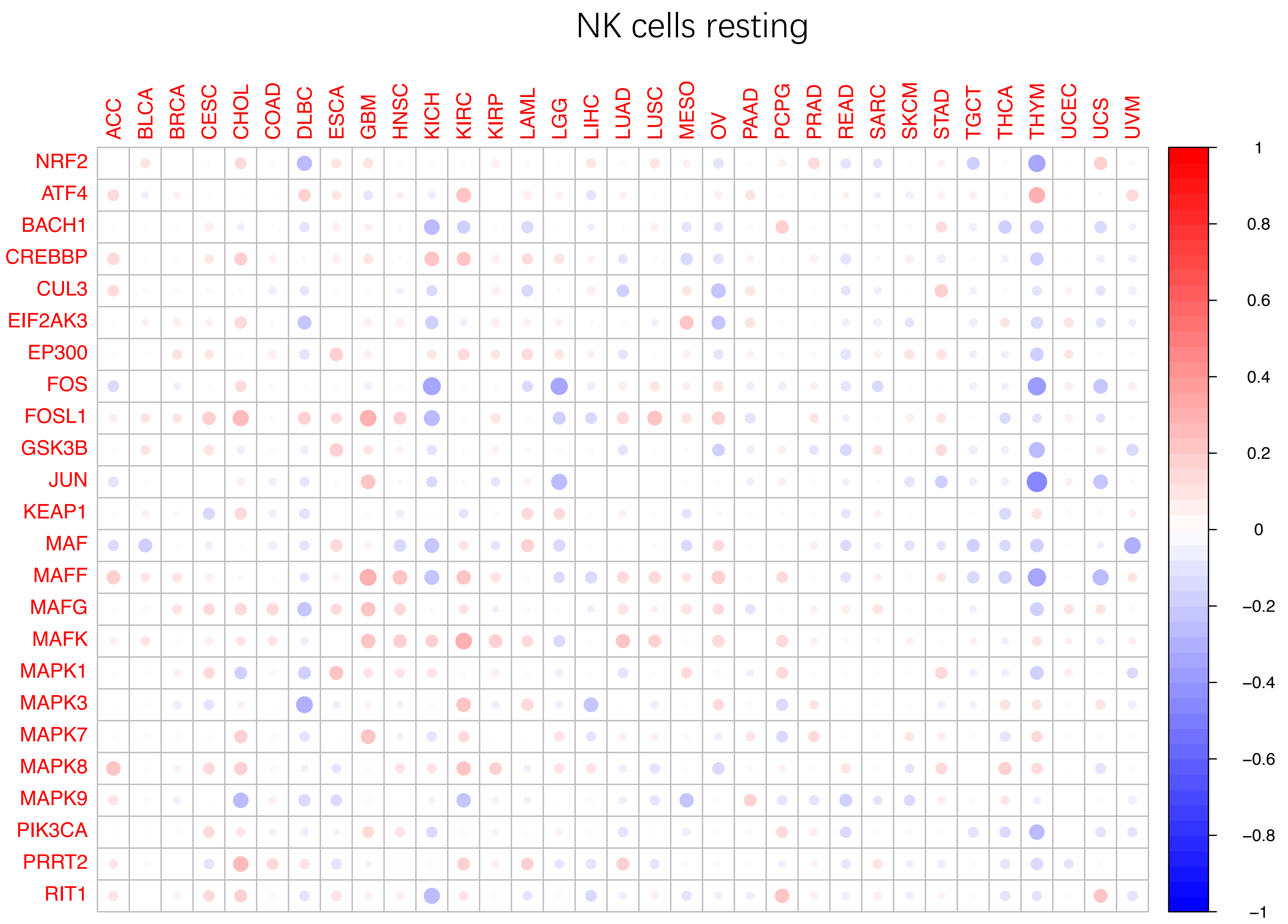
NK cells resting
ACC
BLCA
BRCA
CESC
CHOL
COAD
DLBC
ESCA
GBM
HNSC
KICH
KIRC
KIRP
LAML
LGG
LIHC
LUAD
LUSC
MESO
OV
PAAD
PCPG
PRAD
READ
SARC
SKCM
STAD
TGCT
THCA
THYM
UCEC
UCS
UVM
NRF2
ATF4
BACH1
CREBBP
CUL3
EIF2AK3
EP300
FOS
FOSL1
GSK3B
JUN
KEAP1
MAF
MAFF
MAFG
MAFK
MAPK1
MAPK3
MAPK7
MAPK8
MAPK9
PIK3CA
PRRT2
RIT1
1
0.8
0.6
0.4
0.2
0
−0.2
−0.4
−0.6
−0.8
−1

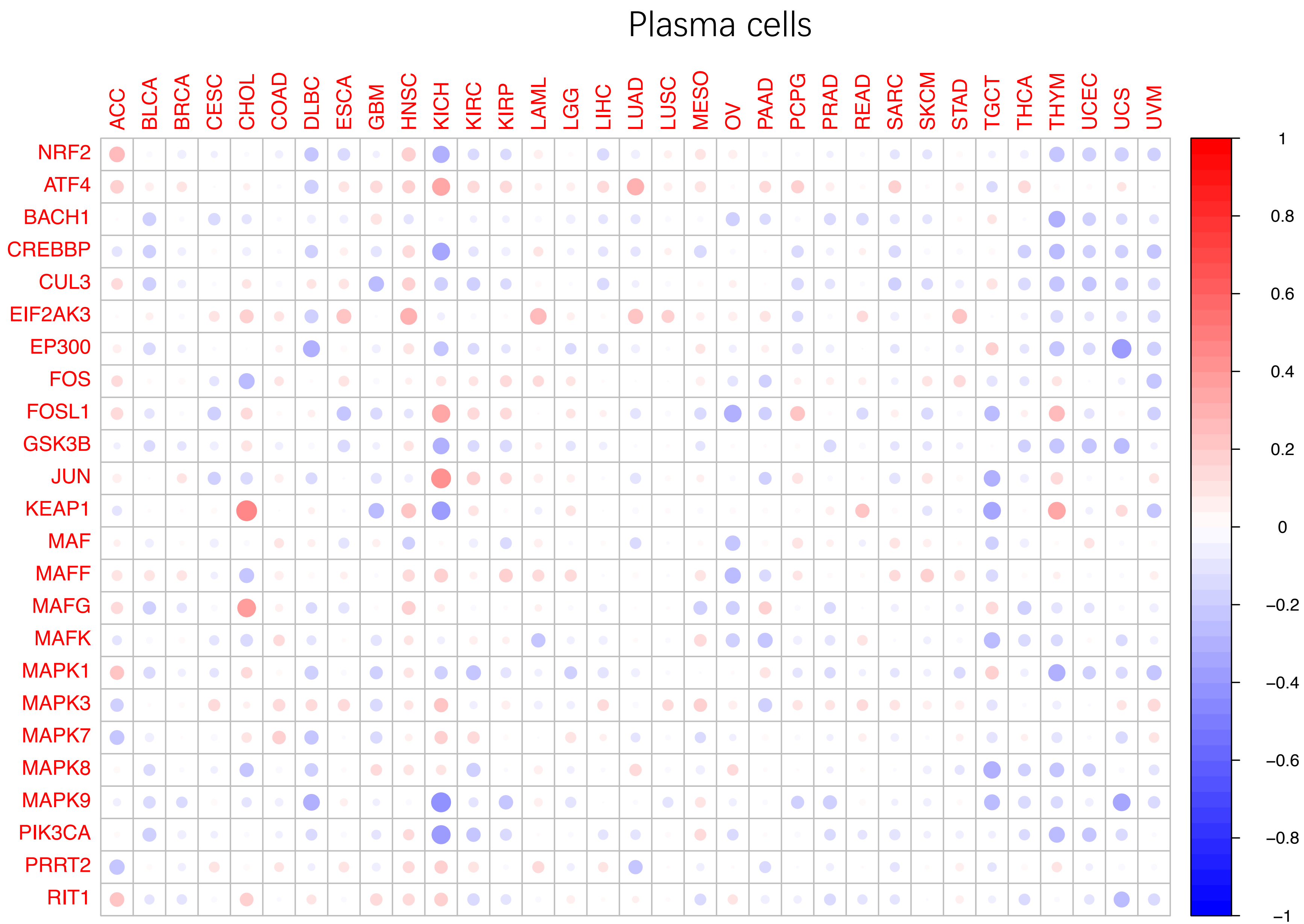
Plasma cells
ACC
BLCA
BRCA
CESC
CHOL
COAD
DLBC
ESCA
GBM
HNSC
KICH
KIRC
KIRP
LAML
LGG
LIHC
LUAD
LUSC
MESO
OV
PAAD
PCPG
PRAD
READ
SARC
SKCM
STAD
TGCT
THCA
THYM
UCEC
UCS
UVM
NRF2
ATF4
BACH1
CREBBP
CUL3
EIF2AK3
EP300
FOS
FOSL1
GSK3B
JUN
KEAP1
MAF
MAFF
MAFG
MAFK
MAPK1
MAPK3
MAPK7
MAPK8
MAPK9
PIK3CA
PRRT2
RIT1
1
0.8
0.6
0.4
0.2
0
−0.2
−0.4
−0.6
−0.8
−1

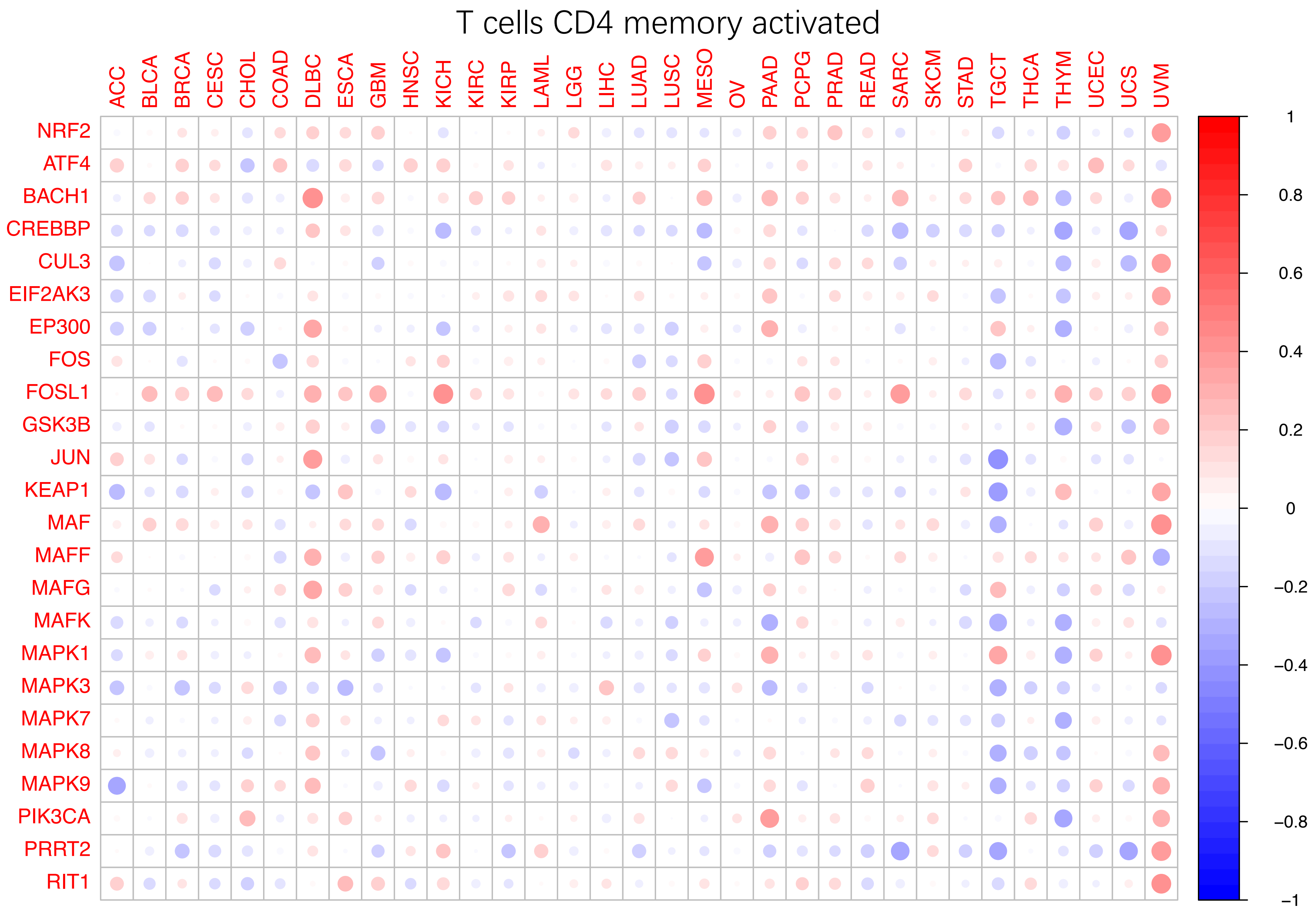
T cells CD4 memory activated
ACC
BLCA
BRCA
CESC
CHOL
COAD
DLBC
ESCA
GBM
HNSC
KICH
KIRC
KIRP
LAML
LGG
LIHC
LUAD
LUSC
MESO
OV
PAAD
PCPG
PRAD
READ
SARC
SKCM
STAD
TGCT
THCA
THYM
UCEC
UCS
UVM
NRF2
ATF4
BACH1
CREBBP
CUL3
EIF2AK3
EP300
FOS
FOSL1
GSK3B
JUN
KEAP1
MAF
MAFF
MAFG
MAFK
MAPK1
MAPK3
MAPK7
MAPK8
MAPK9
PIK3CA
PRRT2
RIT1
1
0.8
0.6
0.4
0.2
0
−0.2
−0.4
−0.6
−0.8
−1

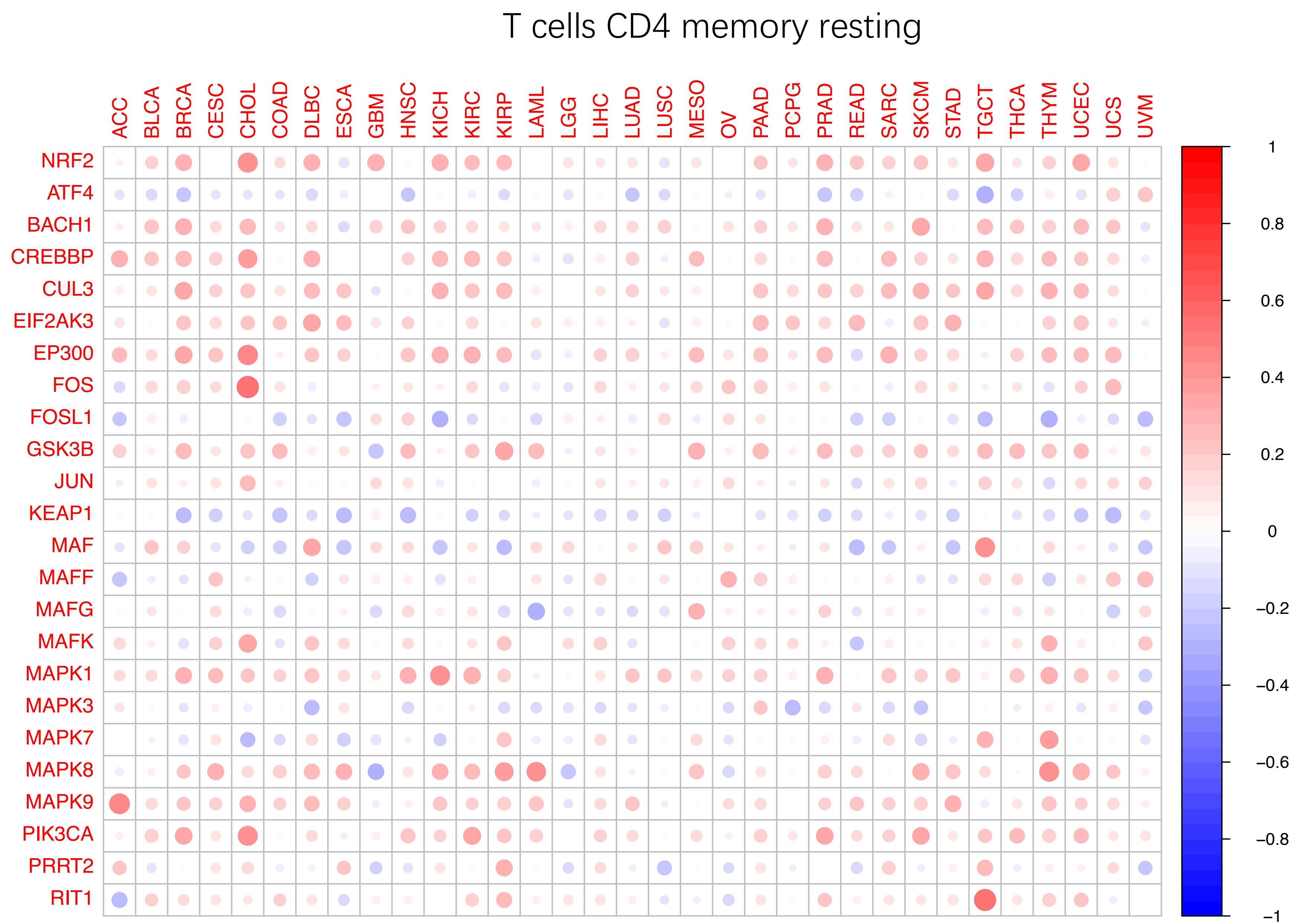
T cells CD4 memory resting
ACC
BLCA
BRCA
CESC
CHOL
COAD
DLBC
ESCA
GBM
HNSC
KICH
KIRC
KIRP
LAML
LGG
LIHC
LUAD
LUSC
MESO
OV
PAAD
PCPG
PRAD
READ
SARC
SKCM
STAD
TGCT
THCA
THYM
UCEC
UCS
UVM
NRF2
ATF4
BACH1
CREBBP
CUL3
EIF2AK3
EP300
FOS
FOSL1
GSK3B
JUN
KEAP1
MAF
MAFF
MAFG
MAFK
MAPK1
MAPK3
MAPK7
MAPK8
MAPK9
PIK3CA
PRRT2
RIT1
1
0.8
0.6
0.4
0.2
0
−0.2
−0.4
−0.6
−0.8
−1

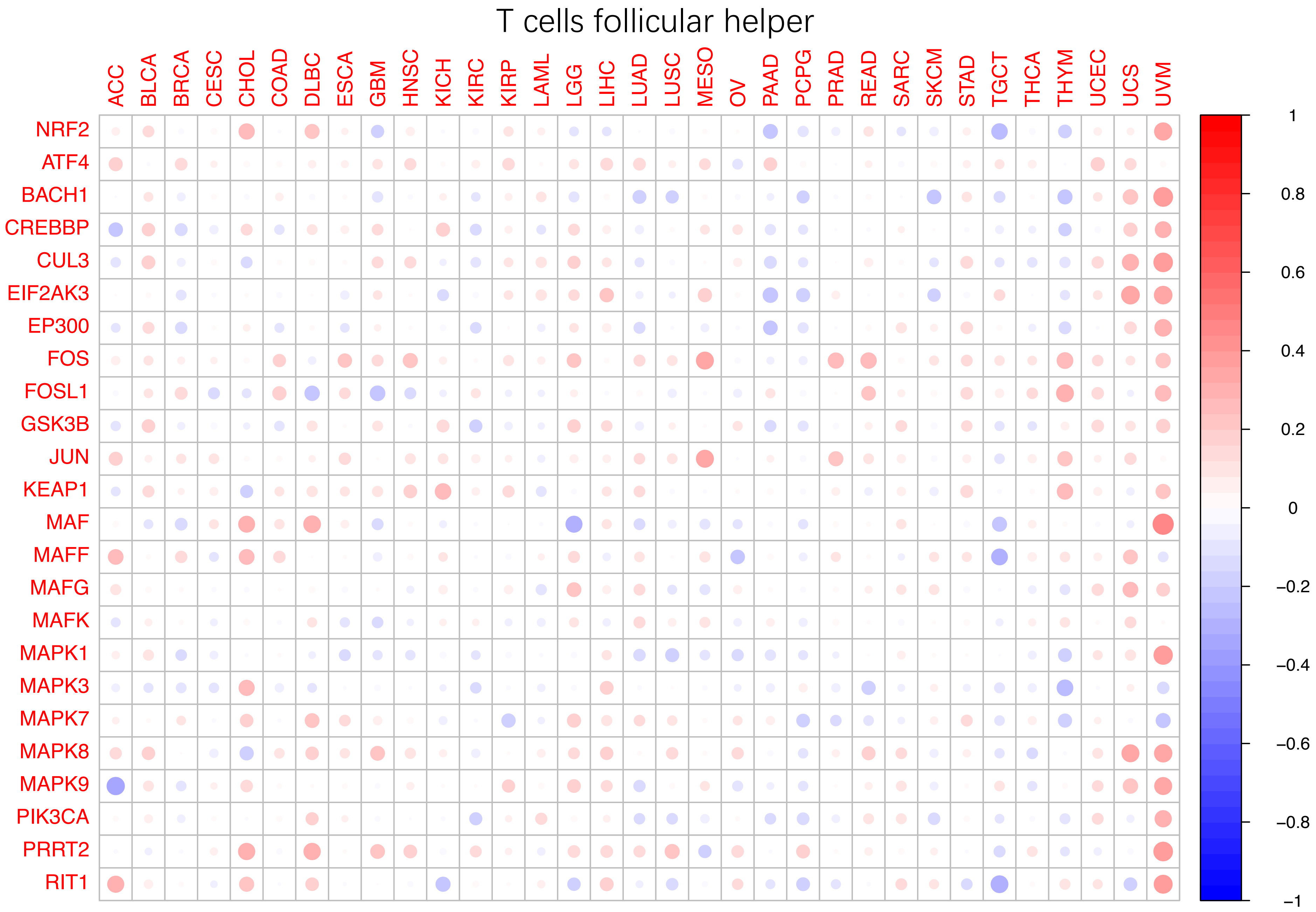
T cells follicular helper
ACC
BLCA
BRCA
CESC
CHOL
COAD
DLBC
ESCA
GBM
HNSC
KICH
KIRC
KIRP
LAML
LGG
LIHC
LUAD
LUSC
MESO
OV
PAAD
PCPG
PRAD
READ
SARC
SKCM
STAD
TGCT
THCA
THYM
UCEC
UCS
UVM
NRF2
ATF4
BACH1
CREBBP
CUL3
EIF2AK3
EP300
FOS
FOSL1
GSK3B
JUN
KEAP1
MAF
MAFF
MAFG
MAFK
MAPK1
MAPK3
MAPK7
MAPK8
MAPK9
PIK3CA
PRRT2
RIT1
1
0.8
0.6
0.4
0.2
0
−0.2
−0.4
−0.6
−0.8
−1

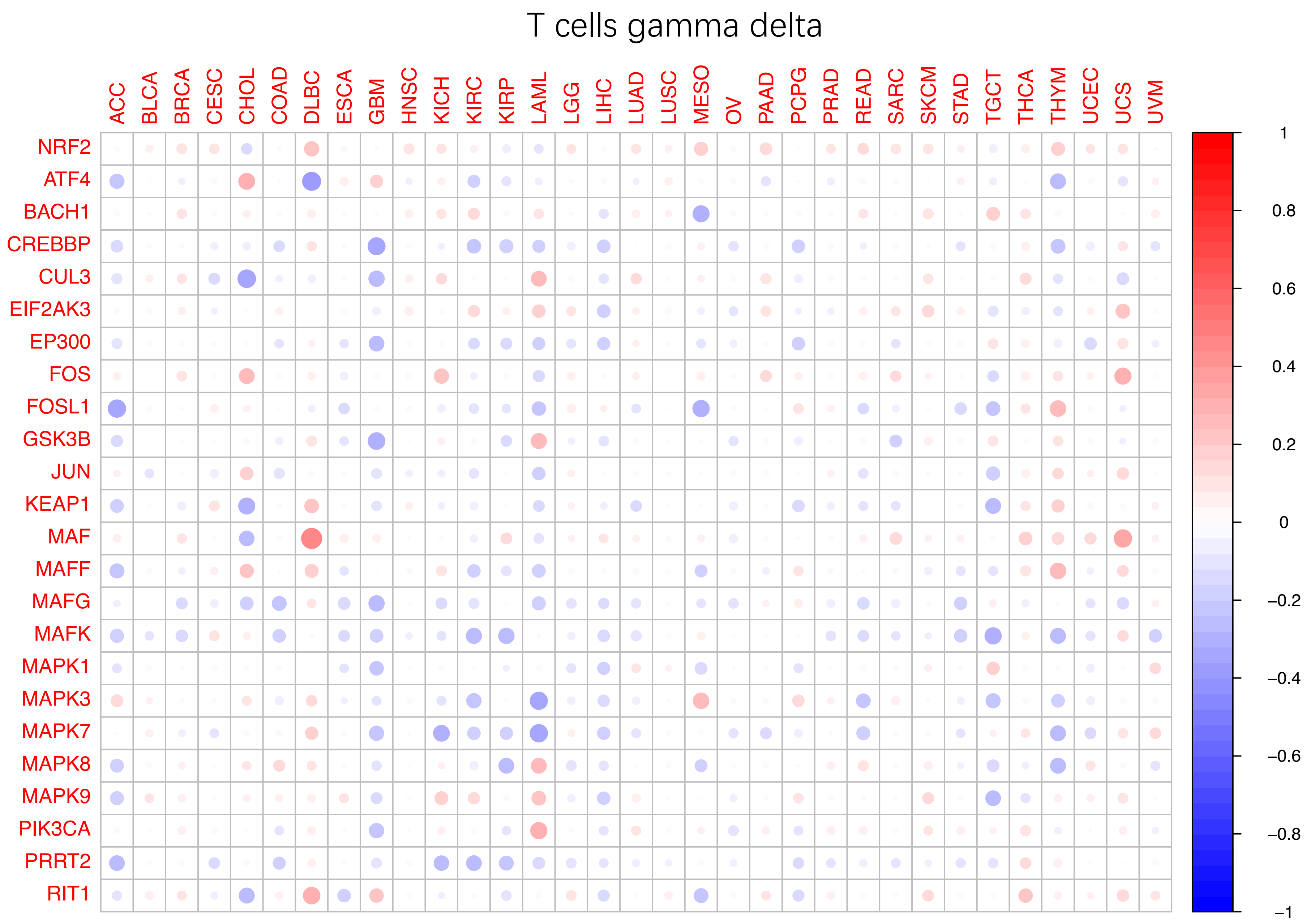
T cells gamma delta
ACC
BLCA
BRCA
CESC
CHOL
COAD
DLBC
ESCA
GBM
HNSC
KICH
KIRC
KIRP
LAML
LGG
LIHC
LUAD
LUSC
MESO
OV
PAAD
PCPG
PRAD
READ
SARC
SKCM
STAD
TGCT
THCA
THYM
UCEC
UCS
UVM
NRF2
ATF4
BACH1
CREBBP
CUL3
EIF2AK3
EP300
FOS
FOSL1
GSK3B
JUN
KEAP1
MAF
MAFF
MAFG
MAFK
MAPK1
MAPK3
MAPK7
MAPK8
MAPK9
PIK3CA
PRRT2
RIT1
1
0.8
0.6
0.4
0.2
0
−0.2
−0.4
−0.6
−0.8
−1

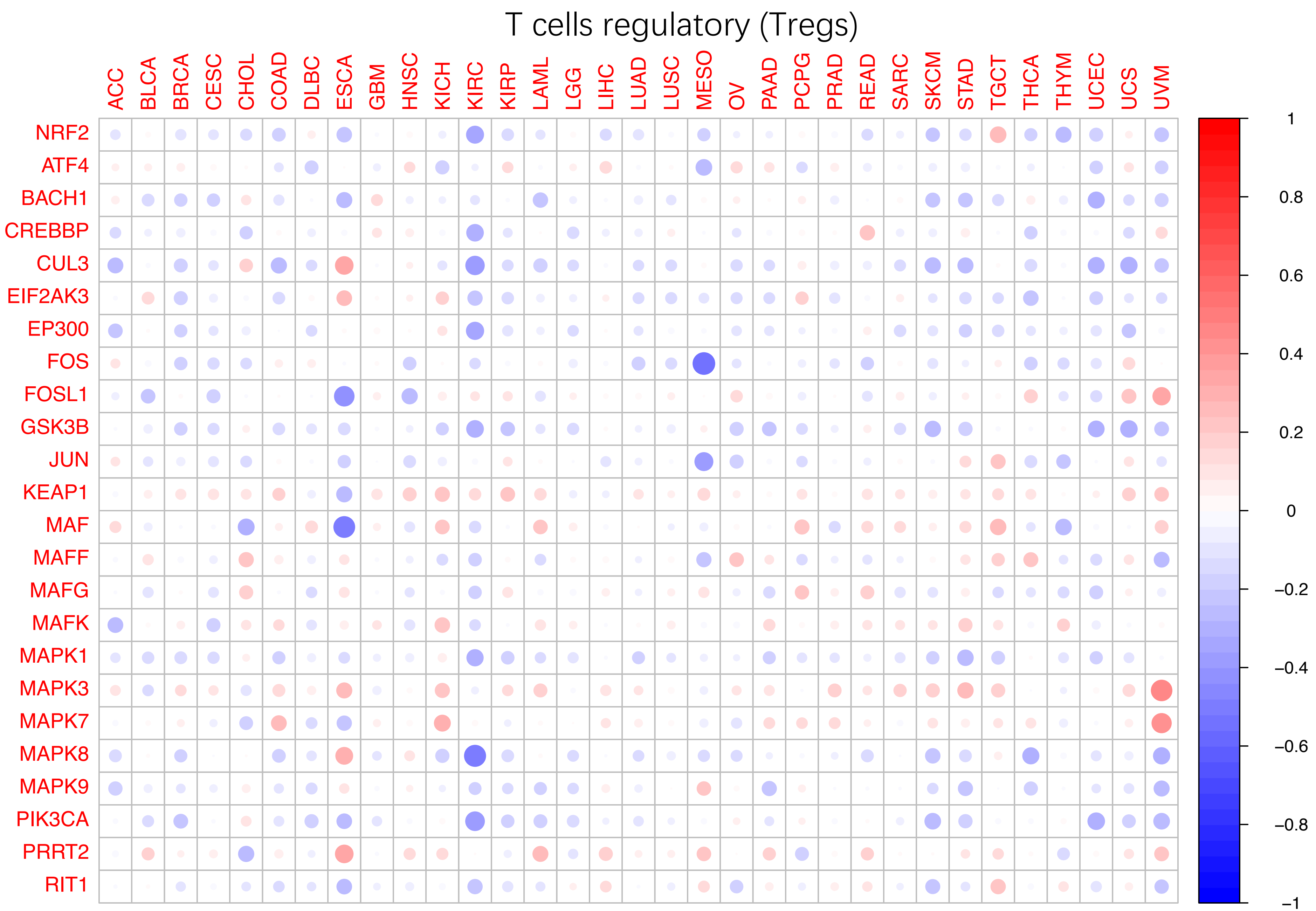
T cells regulatory (Tregs)
ACC
BLCA
BRCA
CESC
CHOL
COAD
DLBC
ESCA
GBM
HNSC
KICH
KIRC
KIRP
LAML
LGG
LIHC
LUAD
LUSC
MESO
OV
PAAD
PCPG
PRAD
READ
SARC
SKCM
STAD
TGCT
THCA
THYM
UCEC
UCS
UVM
NRF2
ATF4
BACH1
CREBBP
CUL3
EIF2AK3
EP300
FOS
FOSL1
GSK3B
JUN
KEAP1
MAF
MAFF
MAFG
MAFK
MAPK1
MAPK3
MAPK7
MAPK8
MAPK9
PIK3CA
PRRT2
RIT1
1
0.8
0.6
0.4
0.2
0
−0.2
−0.4
−0.6
−0.8
−1
